# Proceedings of the 2019 Childhood Arthritis and Rheumatology Research Alliance (CARRA) Annual Scientific Meeting: Louisville, KY, USA. 10-13 April 2019

**DOI:** 10.1186/s12969-019-0373-y

**Published:** 2019-12-03

## A1 Association of HLA-B types in juvenile inflammatory arthritis

### Stephen Wong, Landi Luo, Alice Hoftman, Miriam Parsa, J. Patrick Whelan, Deborah McCurdy, James Louie

#### University of California Los Angeles, Los Angeles, CA, USA

##### **Correspondence:** Stephen Wong

Background : Juvenile inflammatory arthritis is the most common childhood rheumatic disease. The etiology remains unclear, although both genetic and environmental factors likely play a role. Certain HLA-B alleles are associated with juvenile arthritidies including HLA-B27 in juvenile PsA (JPsA) and enthesitis-related arthritis (ERA). While current ILAR classification criteria for JIA utilizes HLA-B27 as both inclusion and exclusion criteria, the potential use of other HLA-B alleles remains unproven. With this retrospective study, we will begin to explore whether an increased expression in certain HLA-B alleles is associated with specific subtypes of juvenile arthritis.

Methods : We derived reports of patients with JIA, JPsA, and ERA who are cared for by experienced pediatric rheumatologists at a tertiary academic medical center. Patients with HLA-B locus testing by reverse sequence specific oligonucleotide hybridization were included in the study. Background demographics including age, sex, and ethnicity were collected. Non-arthritic autoimmune controls consisted of jSLE and vasculitis patients, while the US National Marrow Donor Program provided race-matched healthy controls. Exact binomial tests were performed with a significance level of 0.05. With multiple comparisons, false discovery rate (FDR) was controlled using Benjamini-Hochberg method with a preset α level of 0.20. UCLA IRB approval was obtained.

Results : In the JPsA group (n=22), HLA-B39 and B65 was significantly increased compared to national controls (Fig 1). In the JIA and ERA group (n=87), HLA-B27, B35, B39, B61, and B72 were significantly increased (Fig 2). These HLA-B types were not increased in the non-arthritic autoimmune control group (n=11). After FDR correction, HLA-B27 and B35 in the JIA group remained highly statistically significant.

Conclusions : Multiple HLA-B alleles were found to be associated with juvenile inflammatory arthritis including HLA-B27 and B35, while HLA-B39 and B65 in JPsA, and HLA-B39, B61, and B72 in JIA/ERA approached significance. With continued research, complete HLA-B typing may be of benefit in aiding in diagnosing and sub-categorizing juvenile inflammatory arthritis, and ultimately lead to improved prognostication and effective treatment protocols.

Proportions of HLA-B Types for the JPsA cohort compared to race-matched healthy controls obtained from the US National Marrow Donor Program. One-sided 95% confidence intervals for the JPsA cohort were included. Since the control proportions fell outside the confidence intervals for HLA-B39 and B65, JPsA proportions were significantly greater than the control proportions for HLA-B39 and B65 at significance level = 0.05.

Proportions of HLA-B Types for the combined JIA and ERA cohort compared to race-matched healthy controls obtained from the US National Marrow Donor Program. One-sided 95% confidence intervals for the JIA/ERA cohort were included. Since the control proportions fell outside the confidence intervals for HLA-B27, B35, B39, B61, and B72, JIA/ERA proportions were significantly greater than the control proportions for these HLA-B alleles at significance level = 0.05.

**Fig. 1 (abstract A1).**
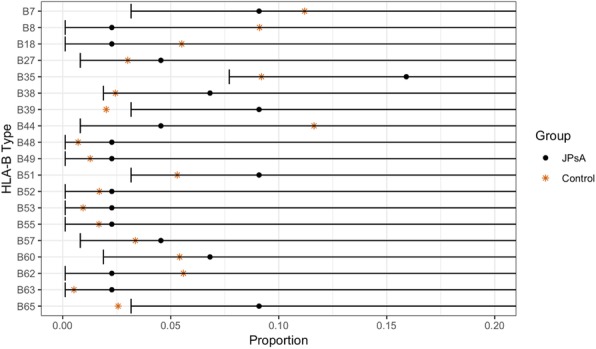
One-Sided 95% CI of HLA-B Type for JPsA vs. Control

**Fig. 2 (abstract A1).**
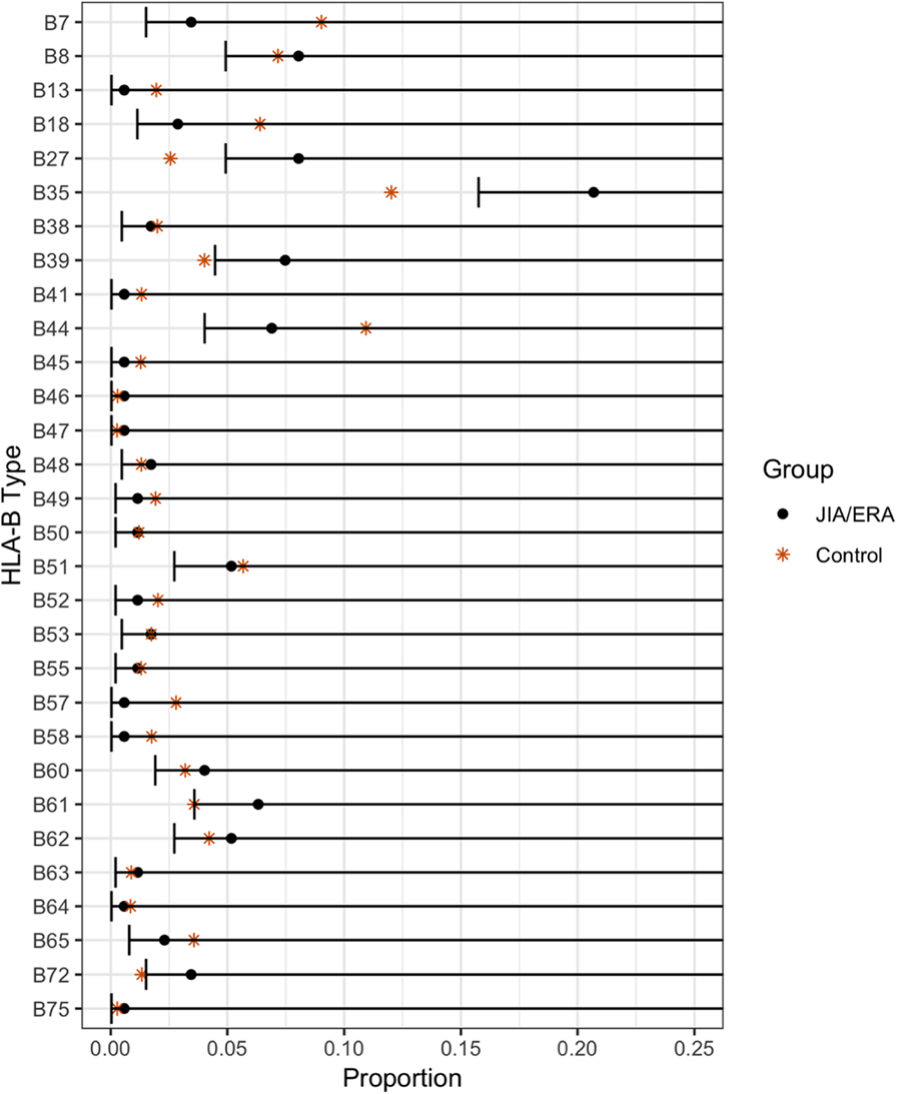
One-Sided 95% CI of HLA-B Type for JIA/ERA vs. Control

## A2 Improving Eye Screening Among Pediatric Rheumatology Patients Receiving Hydroxychloroquine: Experience of a Quaternary Care Center

### Ohoud AlAhmed^155^, Stacy Ardoin^155^, Amanda Way^156^, Darby MacDonald^155^, Kelly Wise^155^, Stephanie Lemle^155^, Shoghik Akoghlanian^155^, Fatima Barbar-Smiley^155^, Vidya Sivaraman^155^

#### ^155^Nationwide Children's Hospital, Columbus, OH, USA; ^156^Nationwide Children's Hospital/Pediatric Ophthalmology Associates, Columbus, OH, USA

##### **Correspondence:** Ohoud AlAhmed

Background : Hydroxychloroquine (HCQ) is widely used to treat autoimmune diseases. Retinopathy from HCQ has been recognized for many years with estimated overall prevalence of 7.5%. Importantly, HCQ-associated retinopathy is irreversible and may progress even after stopping the medication. In 2016, the American Academy of Ophthalmology (AAO) published revised recommendations of eye screening for patients receiving HCQ. Only 65% of patients receiving HCQ in our rheumatology practice were screened in concordance with these recommendations. We developed a quality improvement (QI) initiative which aims to increase the eye screening compliance in patients receiving HCQ and are seen in rheumatology clinic from 65% to 85% in 9 months and to sustain the improvement for 12 months.

Methods : Patients who had HCQ on their list of medications followed in rheumatology clinic at Nationwide Children's Hospital from 1/01/2017 to 8/31/2017 were included in the baseline analysis. We formed a multidisciplinary team of rheumatologists, ophthalmologists, clinical pharmacist, clinic nurses, quality data technician and administrative staff. We formulated consensus eye screening guidelines based on the AAO recommendations and developed a risk-level-based algorithm (Figure 1). A key driver diagram was used to identify barriers to compliance and determine possible interventions. Major interventions included: 1) education of providers and patients, 2) weekly pre-visit planning (PVP) to identify at risk patients and provide individualized screening recommendations and 3) collaboration with ophthalmology to facilitate same day eye screening.

Results : Baseline performance data included 328 patients. An average of 41 patients (range 30-51) were included in assessing the monthly performance (Figure 2). Although the primary aim was not met, the project has resulted in increased awareness and improved communication between rheumatology and ophthalmology providers. Barriers to compliance include: 1) provider and patient lack of knowledge, 2) obtaining accurate data reports to reflect the population of interest and 3) system barriers related to applying and tracking interventions in another specialty with different locations and EHR systems.

Conclusions : This study highlights the importance of multidisciplinary team for QI projects that involve more than one department as well as the benefits of PVP in improving eye screening of patients receiving HCQ. Next steps will be directed at fine-tuning our interventions to target non-compliant patients and sharing provider-level data monthly to improve provider engagement.

IRB-exempt.

**Fig. 1 (abstract A2).**
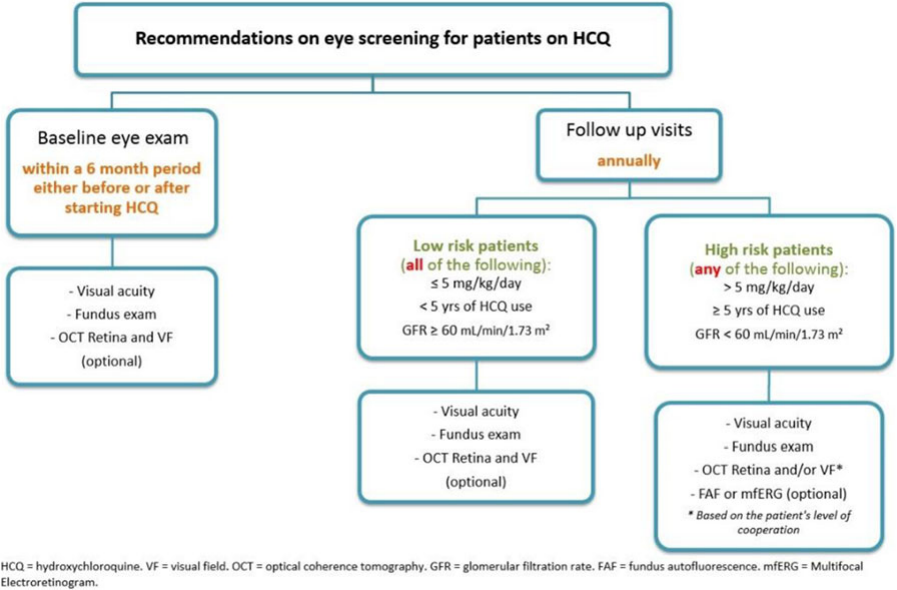
Consensus guidelines and risk-level-based algorithm based on the 2016 revision of the American Academy of Ophthalmology eye screening recommendations

**Fig. 2 (abstract A2).**
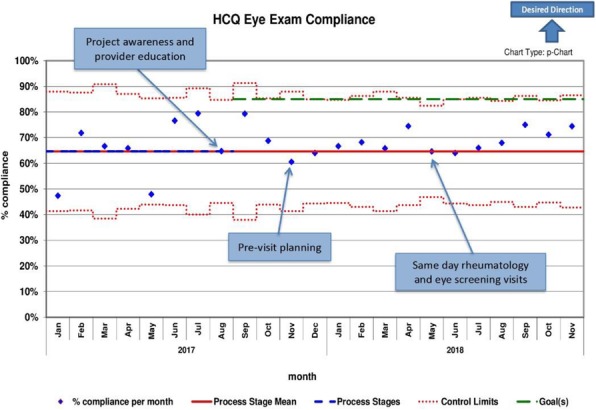
P-chart showing compliance with hydroxychloroquine eye screening guidelines before and after the introduction of this quality improvement project

## A3 Echocardiogram Screening in Pediatric Systemic Lupus Erythematosus and Mixed Connective Tissue Disease – A Single-Center Multi-disciplinary Quality Improvement Initiative to Improve Cardiovascular Health

### Ekemini Ogbu^101^, Kelly Rouster-Stevens^101^, Amit Thakral^101^, Anne Milkowski^49^, Patricia Vega-Fernandez^101^, Larry Greenbaum^101^, Sampath Prahalad^101^

#### ^101^Emory University and Children's Healthcare of Atlanta, Atlanta, GA, USA; ^49^Children's Healthcare of Atlanta, Atlanta, GA, USA

##### **Correspondence:** Ekemini Ogbu

Background : Cardiovascular disease occurs in 25 - 60% of patients with childhood-onset systemic lupus erythematosus (cSLE) and up to 30% with mixed connective tissue disease (MCTD). The echocardiogram (ECHO) has been recommended as the most useful non-invasive method to assess cardiac involvement, especially early preclinical disease. Current best practice is to obtain an ECHO at diagnosis of cSLE or MCTD and annually, particularly if the baseline ECHO is abnormal. We observed a low frequency of ECHO screening in cSLE and MCTD patients at our rheumatology center and thus initiated a quality improvement project to improve the annual rate at our center, with the goal of reaching 80% in 12 months.

Methods : We used a Plan-Do-Study-Act approach. We obtained baseline data from April to June 2017 and conducted a fishbone analysis of barriers to ECHO completion (Figure 1). Our pediatric rheumatologists, fellows and nursing team participated in this project. Our primary and process measures were tracked on individual (pediatric rheumatologists) and aggregate level. Our primary outcome measure was percentage of patients with annual ECHO within the last year of a clinic visit. Our process measures were (i) Physician ordering of ECHOs for patients without annual ECHOs per clinic visit (ii) Completion of ordered ECHO. Our balance measure was duration of clinic visit. Participant education was completed in 2/2018. We have completed three cycles (Figure 2). Our final cycle is in progress (12/2018 to 1/2019).

Results : At baseline, 62% of our patients had ECHOs within a year prior to a clinic visit. In addition, only 20% of the patients without an ECHO within the last year at a clinic visit had an ECHO ordered at that clinic visit. The percentage of patients up to date on annual ECHOs at the time of a clinic visit at the end of cycles 1, 2 and 3 was 83%, 85% and 83% respectively (Figure 2 and 3). Physician ordering of ECHOs for patients without a yearly ECHO was 72%, 52% and 71% at the end of cycles 1, 2 and 3 respectively.

Conclusions : Using a multidisciplinary team approach, we achieved and sustained our goal of increasing annual ECHO screening for our patients with cSLE and MCTD.

IRB approval was not required for this quality improvement project.

**Fig. 1 (abstract A3).**
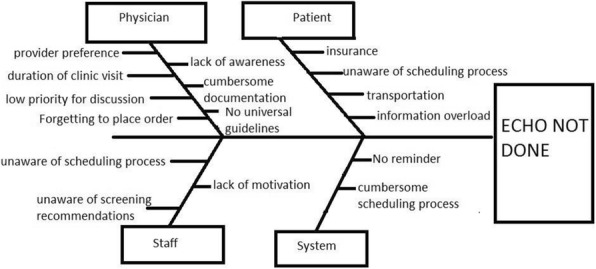
Fishbone Analysis of Barriers to Completing Annual Echocardiogram Screening

**Fig. 2 (abstract A3).**
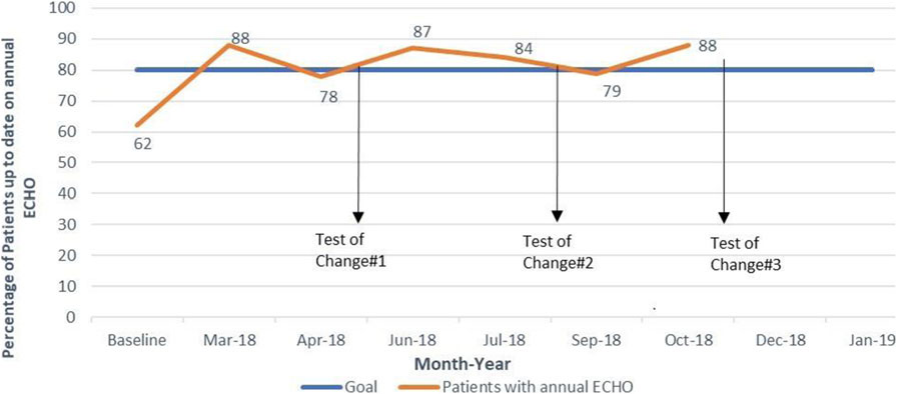
Annual Echocardiogram (ECHO) Screening in Patients with Systemic Lupus Erythematosus and Mixed Connective Tissue Disease

**Fig. 3 (abstract A3).**
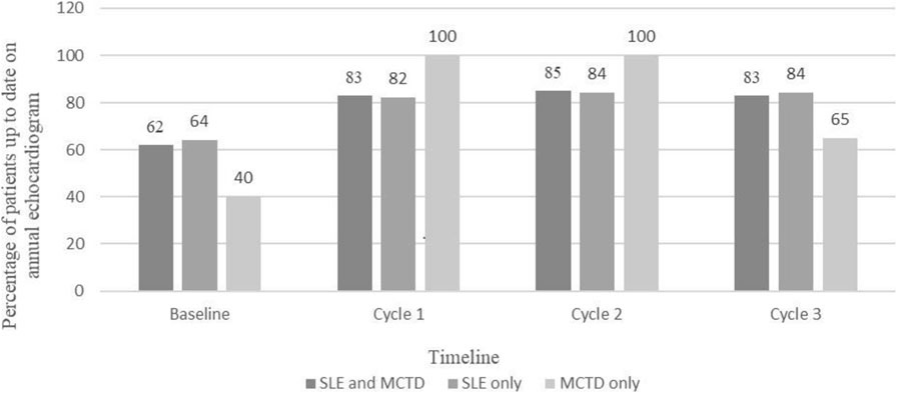
Annual Echocardiogram Screening in Pediatric Patients with Systemic Lupus Erythematosus (SLE) and Mixed Connective Tissue Disease (MCTD)

## A4 Immunogenicity of Pneumococcal Vaccination in Patients with Childhood Onset Systemic Lupus Erythematosus (cSLE): a retrospective chart review

### Anna Zemke^175^, Octavio Ramilo^155^, Stacy Ardoin^155^, Chack-Yung Yu^155^, Vidya Sivaraman^155^, Cagri Yildirim-Toruner^155^, Kelly Wise^155^, Fatima Barbar-Smiley^155^

#### ^175^Ohio State University College of Medicine, Columbus, OH, USA; ^155^Nationwide Children's Hospital, Columbus, OH, USA

##### **Correspondence:** Anna Zemke

Background : Childhood onset systemic lupus erythematosus (cSLE) is associated with significant morbidity and mortality linked to infections. The CDC recommends that immunocompromised patients receive pneumococcal conjugate (PCV13) and polysaccharide (PPSV23) vaccines. However, there is limited evidence about the effectiveness of this vaccination regimen in cSLE. Thus, we aimed to investigate pneumococcal vaccine immunogenicity in a cohort of cSLE patients following the receipt of both PCV13 and PPSV23 vaccines.

Methods : Retrospective chart review of cSLE patients followed in the rheumatology clinics of a large free-standing children’s hospital. Eligible patients are cSLE patients who have received PCV13 and PPSV23 and had post-vaccine titers measured at least 4 weeks after their last pneumococcal vaccination. Adequate immune response was defined as antibody concentration greater than 1.3 μg /ml in 70% or more of measured serotypes measured by Quantitative Multiplex Bead Assay. We reviewed patient demographics, vaccination status and dates, medication use at time of vaccination, and post-vaccination titers. Measured serotypes include: 1, 3, 4, 5, 6B, 7F, 8, 9V, 9N, 12F, 14, 18C, 19F, 23F.

Results : 15/86 cSLE patients met inclusion criteria. 12/15 (80%) were white, 3/15 (20%) African American (AA). 13/15 (87%) were female. Median age was 16.8 years (range: 9-21). 5/15 (33%) had lupus nephritis. Patients were treated with one or more of the following medications: hydroxychloroquine, mycophenolate mofetil, methotrexate, azathioprine, cyclophosphamide, prednisone and methylprednisolone. Median days from last pneumococcal vaccination to post-vaccine titer measurement was 156 days (range 49-315). 12/15 (80%) had adequate immune response in 79-100% of vaccine covered serotypes. 8/15 (53%) did not develop adequate immunity against 12F serotype that is covered by PPSV23 vaccine. The 9V serotype, which is covered with both PPSV23 and PCV13 vaccines, and 9N which is covered by PPSV23 vaccine, did not have adequate immune response in 27% (4/15) patients. 3 patients had inadequate response with seroprotection rates of 14%, 57%, and 64%. The patient with least/weakest serotype response (14%) was female, AA with lupus nephritis who received PCV13/PPSV23 vaccinations during hospital admissions for SLE flare treated with high dose pulse (30 mg/kg) IV glucocorticoids close to time of both vaccinations. This patient had protective titers against 1/14 (7%) serotype pre-vaccination which increased to 2/14 (14%) post-vaccination.

Conclusions : Initial data in this pilot study suggest adequate immune response in most cSLE patients to PCV13/PPSV23 despite treatment with glucocorticoids and immunosuppressive medications at time of vaccination. However, we noted variability with responses to different serotypes with lowest response noted with 9V, 9N and 12F. Poorer response (14% serotype immunity) in one patient may be related to high dose intravenous steroids and SLE disease flare at time of vaccination. This is an ongoing study with future directions to expand study sample size. Larger sample size will help us achieve more statistical power and ability to stratify immune response further based on patient characteristics.

Study was approved by the local institutional review board (IRB).

## A5 Immunomodulatory Medication Use for Youth with Newly-Diagnosed Systemic Lupus Erythematosus

### Andrea Knight^229^, Alaina Davis^146^, Marisa Klein-Gitelman^13^, Jennifer Faerber^117^, Hannah Katcoff^117^, Zuleyha Cidav^285^, David Mandell^285^

#### ^229^The Hospital for Sick Children, Toronto, ON, Canada; ^146^Monroe Carell Junior Children's Hospital at Vanderbilt, Nashville, TN, USA; ^13^Ann & Robert H. Lurie Children’s Hospital of Chicago, Chicago, IL, USA; ^117^Health Analytics Unit, The Children's Hospital of Philadelphia, Philadelphia, PA, USA; ^285^University of Pennsylvania, Philadelphia, PA, USA

##### **Correspondence:** Andrea Knight

Background : Youth with systemic lupus erythematosus (SLE) are at risk for organ damage due to disease activity. Early treatment after diagnosis with SLE is important to reduce disease damage. We examined immunomodulatory medication use for youth with SLE during their first year of care.

Methods : We conducted a retrospective cohort study using de-identified administrative claims for 2000 to 2013 from Optum© Clinformatics® DataMart for youth ages 10-24 years with an incident diagnosis of SLE (≥3 International Classification of Diseases, Ninth Revision codes for SLE 710.0, each >30 days apart). We determined the proportion of subjects filling a prescription for an immunomodulatory mediation, defined as hydroxychloroquine or an immunosuppressant (excluding glucocorticoids), within 3, 6, and 12 months after the first SLE diagnosis code (index date). We used a Cox proportional hazards regression model to examine associations between time to immunomodulatory prescription fill within 12 months and demographic and disease factors (age, race/ethnicity, household education level, region, history of seizures/stroke, history of nephritis).

Results : We identified 650 youth with an incident diagnosis of SLE. In the 12 months following the index date, 511 (79%) of youth had a prescription fill for an immunomodulatory medication. For those with a prescription fill for hydroxychloroquine in the first year (n=457, 70%), 374 (58%) and 407 (63%) of youth filled the medication within 3 months and 6 months from the index date, respectively (Table 1). For those with a prescription fill for an immunosuppressant (n=221, 34%) in the first year, 114 (18%) and 162 (25%) of youth filled the medication within 3 months and 6 months from the index date, respectively (Table 1). Location in the Northeast region was significantly associated with a longer time to immunomodulatory prescription fill within 12 months, compared to location in the South (HR=0.686, 95% CI 0.50-0.94). There were no statistically significant associations for the other demographic and disease factors.

Conclusions : Among youth with newly-diagnosed SLE, hydroxychloroquine use is prevalent although not universal, and immunosuppressant use is notably low during the first year of care. As poorly controlled SLE disease activity can lead to organ damage, further work is needed to identify potential factors contributing to suboptimal immunomodulatory medication use in this population. The study did not meet criteria for human subjects research, and was exempted from review by the Institutional Review Board at The Children's Hospital of Philadelphia.

Acknowledgements: The study was funded by the CARRA-Arthritis Foundation Large Grant Award and Publication Grant Award, and supported by the Rheumatology Research Foundations and National Institute of Arthritis and Musculoskeletal and Skin Diseases. The authors wish to acknowledge the ongoing Arthritis Foundation financial support of CARRA.

**Table 1 (abstract A5).** Immunomodulatory Medication Use in Youth with Newly-Diagnosed SLE, N=650

Proportion with prescription fills after first SLE diagnosis code, n (%)	Within 3 months	Within 6 months	Within 1 year
Immunomodulatory medication (hydroxychloroquine or immunosuppressant)	428 (66)	460 (71)	511 (78)
Hydroxychloroquine	374 (58)	407 (63)	457 (70)
Immunosuppressant	114 (18)	162 (25)	221 (34)

Immunosuppressant medications include: mycophenolate mofetil, azathioprine, leflunomide, methotrexate, tacrolimus, and oral cyclophosphamide

## A6 Development of Novel Urinary Biomarkers for Lupus Nephritis

### Natalie Rosenwasser^120^, Seunghee Hong^315^, Lynnette Walters^220^, Phuong Nguyen^27^, Marina Ohouo^315^, Cindy Wang^120^, Yuanyuan Wang^27^, Cynthia Smitherman^27^, Xuan Wang^27^, Jeanine Baisch^315^, Simone Caielli^315^, Tracey Wright^305^, Karen Onel^120^, Virginia Pascual^315^

#### ^120^Hospital for Special Surgery, New York, NY, USA; ^315^Weill Cornell Medicine, New York, NY, USA; ^220^Texas Scottish Rite Hospital, Dallas, TX, USA; ^27^Baylor Scott and White, Houston, TX, USA; ^305^UT Southwestern Medical Center, Dallas, TX, USA

##### **Correspondence:** Natalie Rosenwasser

Background : Lupus Nephritis is a source of considerable morbidity and mortality in children with Systemic Lupus Erythematosus (cSLE). Kidney biopsies are widely used to accurately diagnose nephritis class and evaluate responsiveness to treatments, but they are invasive. Renal function tests provide non-specific information, and there are no additional biomarkers to replace kidney biopsies. Blood transcriptional profiling has contributed to improve our understanding of disease pathogenesis and may be a source of non-invasive biomarkers. Whether urinary cells may provide insight into cSLE disease pathogenesis, however, has not been explored before.

Methods : In this study, we quantified 255 cell-associated urinary transcripts from 82 cSLE, including 43 with different classes of LN, using the nanoString nCounter Human Inflammation v2 assay.

Results : Six healthy control samples were obtained but did not provide sufficient RNA for reliable interpretation and were not included in the analysis. Normalization and unsupervised clustering analysis of the patient’s samples revealed higher transcriptional activity correlating with disease activity (as evidenced by the SLE Disease Activity Index scoring or SLEDAI). In particular, samples from mixed (class III/IV + V) nephritis class, which displayed the highest SLEDAI scores, showed strong interferon and inflammasome-related signatures compared to other nephritis classes. Interestingly, samples from cSLE patients with no known nephritis also displayed an interferon signature. Samples from patients with class II (mesangial) nephritis uniquely downregulated C3 (complement 3) transcripts. Among the observed signatures, therapeutically targetable pathways were identified in a number of patients (especially in the mixed class).

Conclusions : These studies are being currently extended using an independent group of samples from patients with and without nephritis to confirm the value of urinary transcriptional profiling as a source of biomarkers to follow disease activity and identify potential therapeutic targets in cSLE.

Acknowledgements: This project is funded by a CARRA-Arthritis Foundation grant. The authors wish to acknowledge the ongoing Arthritis Foundation financial support of CARRA.

## A7 Reproductive Health Awareness and Needs: Assessment of Parents, Female Adolescents, and Young Adults with Pediatric Rheumatic Diseases

### Veronica Mruk^153^, Kristine Carandang^264^, Stacy Ardoin^155^, Elise Berlan^153^, Kathy Vannatta^231^, Rebecca Furru^153^, Megan Clowse^96^, Cuoghi Edens^273^

#### ^153^Nationwide Children's Hospital / The Ohio State University, Columbus, OH, USA; ^264^University of California / Child and Adolescent Services Research Center, San Diego, CA, USA; ^155^Nationwide Children's Hospital, Columbus, OH, USA; ^231^The Ohio State University, Columbus, OH, USA; ^96^Duke University Medical Center, Durham, NC, USA; ^273^University of Chicago, Chicago, IL, USA

##### **Correspondence:** Veronica Mruk

Background : A diagnosis of a pediatric rheumatic disease comes with worries for both parents and patient; one that may be overlooked is the impact on long- and short- term reproductive health, including the impact of the disease and treatment on puberty, sexuality, fertility, pregnancy, and contraception. Little exploration has been done to assess the reproductive health needs and concerns of parents and patients with pediatric rheumatic diseases. We sought to identify areas of reproductive health that most concern girls and young women with pediatric rheumatic diseases and their parents as well as obstacles to reproductive health knowledge in this population.

Methods : Females 15-20 years old diagnosed with any rheumatic condition and their parents were recruited from the Nationwide Children’s Hospital Pediatric Rheumatology Clinic. Participants completed a survey collecting demographics, diagnosis, rheumatic medications, sexual activity, and contraception use and engaged in one of three focus groups: two age-specific patient groups and one parent group. A focus group discussion guide was used, with a goal of identifying areas of concern regarding reproductive health, topics in which pediatric patients/parents need more information, barriers to current knowledge, current sources of knowledge, and suggestions for ways to distribute information. Each audio-recorded focus group was led by a pediatric psychology researcher and an internal medicine-pediatric rheumatologist; it was professionally transcribed and will be coded using constant comparative analysis.

Results : A total of 9 patients and 7 parents participated. The majority of patients were Caucasian (n=7; 78%) and carried the diagnosis of JIA (n=6; 67%), with a small representation of systemic lupus (n=2; 22%) and vasculitis (n=1; 11%). In the parent group, over 50% of parents had some level of college education and had household incomes over the median national average. Close to two-thirds of participants were on teratogenic medications. 11% of patients were sexually active with 55% of total subjects were using contraception. When discussing teratogenic medications, all focus groups expressed concerns about the interaction of rheumatic medications with contraception and fertility. In addition, participants expressed worry regarding potential future pregnancy complications, including how their rheumatic disease would affect the health of themselves and their offspring. Participants reported varied levels of reproductive health education, ranging from none to in-depth discussions with fertility specialists, with a general agreement of dissatisfaction with available information.

Conclusions : Reproductive health is an important topic to female adolescents and young adults with rheumatic diseases, and their parents, even if not sexually active. The most prominent concern for all groups was medications and their impact on many aspects of reproductive health. Our future steps include understanding the scope of information that is needed, barriers to this information, and developing tools to enable pediatric rheumatologists, families, and patients to address these concerns accurately and easily.

The study was approved by Nationwide Children’s Hospital Institutional Review Board (IRB), number IRB18-00589. Informed consent to publish has been obtained from the participants.

## A8 Long-Term Safety of Subcutaneous Tocilizumab Administration in Systemic and Polyarticular Juvenile Idiopathic Arthritis

### Hermine Brunner^193^, Nicola Ruperto^194^, Alberto Martini^178^, Athimalaipet Ramanan^37^, Ruben Cuttica^124^, Jennifer Weiss^115^, Michael Henrickson^193^, Heinrike Schmeling^11^, Jordi Anton^185^, Kirsten Minden^46^, Gerd Horneff^20^, Maria Luz Gámir Gámir^125^, Markus Hufnagel^257^, Wendy Douglass^200^, Chris Wells^200^, Navita L. Mallalieu^199^, Daniel Lovell^193^, Fabrizio De Benedetti^132^

#### ^193^PRCSG, Cincinnati Children’s Hospital Medical Center, Cincinnati, OH, USA; ^194^PRINTO, Instituto Gaslini, Genova, Italy; ^178^Paediatric Rheumatology International Trial Organisation (PRINTO) Coordinating Centre, Genova, Italy; ^37^Bristol Royal Hospital for Children, Bristol, England; ^124^Hospital General de Niños Pedro de Elizalde, Buenos Aires, Argentina; ^115^Hackensack University Medical Center, Hackensack, NJ, USA; ^11^Alberta Children’s Hospital/University of Calgary, Calgary, AB, Canada; ^185^Pediatric Rheumatology, Hospital Sant Joan de Déu, Universitat de Barcelona, Barcelona, Spain; ^46^Charité–Universitätsmedizin Berlin, Berlin, Germany; ^20^Asklepios Clinic Sankt Augustin, Sankt Augustin, and University Hospital of Cologne, Sankt Augustin, Germany; ^125^Hospital Ramon y Cajal Unidad de Reumatologia Pediatrica, Madrid, Spain; ^257^University Medical Center, Medical Faculty, University of Freiburg, Freiburg im Breisgau, Germany; ^200^Roche Products Ltd., Welwyn Garden City, UK; ^199^Roche Innovation Center, New York, NY, USA; ^132^IRCCS Ospedale Pediatrico Bambino Gesù, Rome, Italy

##### **Correspondence:** Hermine Brunner

Background : Tocilizumab (TCZ) administered intravenously (IV) was shown to be effective in polyarticular and systemic juvenile idiopathic arthritis (pJIA and sJIA). An ongoing 5-year, long-term extension (LTE) of two 52-week, open-label studies in patients (pts) with pJIA and sJIA is evaluating long-term safety and efficacy of subcutaneous (SC) TCZ; safety results at the clinical cutoffs December 1, 2017 (pJIA), and February 28, 2018 (sJIA), are reported.

Methods : Pts aged 1-17 years received body weight (BW)–based TCZ SC dosing regimens: pJIA pts received TCZ 162 mg every 3 weeks for BW <30 kg or every 2 weeks for BW ≥30 kg; sJIA pts received TCZ 162 mg every 10 days for BW <30 kg or weekly for BW ≥30 kg. pJIA pts had failed MTX treatment or could not tolerate MTX; sJIA pts had inadequate response to NSAIDs and glucocorticoids. All pts had to discontinue biologic DMARDs. Approximately 50% of the patients switched to SC from IV TCZ. After 52 weeks, pts continued TCZ treatment according to BW in the LTE; we report safety data for pJIA and sJIA as adverse events (AEs), serious AEs (SAEs), and AEs of special interest (AESIs). The safety populations included all pts who received ≥1 dose of TCZ SC and had ≥1 postdose safety assessment.

Results : Of pJIA pts (n = 44), 72.7% were female and 88.6% were white. Of sJIA pts (n = 38), 55.3% were female and 84.2% were white. Median (range) age in both groups was 9.0 (2-18) years. AE rates (Table 1) were similar regardless of BW. Most AEs were grade 1 or 2; grade ≥3 AEs were reported by 10 of 44 (20.8%) pJIA pts and 4 of 38 (10.5%) sJIA pts. The most common AE was nasopharyngitis in both pJIA (17/44 [38.6%]) and sJIA (11/38 [28.9%]) pts. Other AEs reported in ≥15% of pts were arthralgia, gastroenteritis, cough, vomiting, diarrhea, pyrexia, headache, and oropharyngeal pain in pJIA and upper respiratory tract infection, cough, pyrexia, arthralgia, and rash in sJIA. No opportunistic infections developed. Neutropenia AEs were reported by 6 (13.6%) pJIA pts and 7 (18.4%) sJIA pts. Of pJIA pts, 5 of 44 (11.4%) experienced SAEs (furuncle, appendicitis, pneumonia, eye pain/headache, infectious mononucleosis); only pneumonia was considered treatment related. Of sJIA pts, 2 of 38 (5.3%) experienced SAEs (pneumonia, craniocerebral injury from a fall); neither was considered treatment related. Neutralizing anti-TCZ antibodies developed in 2 (4.7%) pJIA pts and 0 sJIA pts. No deaths were reported in the LTE as of the data cutoff.

Conclusions : In this LTE study in children with pJIA or sJIA, SC TCZ continues to have an acceptable tolerability profile with no new safety concerns.

**Table 1 (abstract A8).** Safety Summary

	pJIA		sJIA			
	TCZ SC Q3W (<30 kg) n = 24	TCZ SC Q2W (≥30 kg) n = 20	All TCZ SC N = 44	TCZ SC Q10D or Q2W (<30 kg) n = 19	TCZ SC QW (≥30 kg) n = 19	All TCZ SC N = 38
Duration in study, PY	52.3	48.8	101.1	23.4	44.0	67.3
AEs						
Pts, n (%)	24 (100)	20 (100)	44 (100)	17 (89.5)	17 (89.5)	34 (89.5)
n	269	249	518	136	222	358
rate/100 PY (95% CI)	514.0 (454.5, 579.3)	510.7 (449.2, 578.2)	512.4 (469.2, 558.5)	582.2 (488.5, 688.7)	504.8 (440.6, 575.7)	531.6 (478.0, 589.7)
SAEs						
Pts, n (%)	2 (8.3)	3 (15.0)	5 (11.4)	1 (5.3)	1 (5.3)	2 (5.3)
n	2	4	6	1	1	2
rate/100 PY (95% CI)	3.8 (0.5, 13.8)	8.2 (2.2, 21.0)	5.9 (2.2, 12.9)	4.3 (0.1, 23.9)	2.3 (0.1, 12.7)	3.0 (0.4, 10.7)
AEs leading to withdrawal						
Pts, n (%)	2 (8.3)	1 (5.0)	3 (6.8)	0	0	0
n	2	1	3	0	0	0
rate/100 PY (95% CI)	3.8 (0.5, 13.8)	2.1 (0.1, 11.4)	3.0 (0.6, 8.7)			
AEs leading to dose interruption						
Pts, n (%)	11 (45.8)	2 (10.0)	13 (29.5)	4 (21.1)	6 (31.6)	10 (26.3)
n	21	3	24	7	14	21
rate/100 PY (95% CI)	40.1 (24.8, 61.3)	6.2 (1.3, 18.0)	23.7 (15.2, 35.3)	30.0 (12.0, 61.7)	31.8 (17.4, 53.4)	31.2 (19.3, 47.7)

AEs, adverse events; pJIA, polyarticular juvenile idiopathic arthritis; Pts, patients; PY, patient-years; Q2W, every 2 weeks; Q3W, every 3 weeks; Q10D, every 10 days; QW, weekly; SAEs, serious adverse events; SC, subcutaneous; sJIA, systemic juvenile idiopathic arthritis; TCZ, tocilizumab

## A9 Assessment of the TMJ in Juvenile Idiopathic Arthritis Using Acoustic Emissions Generated from Jaw Movements in Two Planes

### Daniel Whittingslow^110^, Lara Orlandic^110^, Talia Gergely^49^, Lori Ponder^102^, Sampath Prahalad^102^, Omer Inan^110^, Shelly Abramowicz^102^

#### ^110^Georgia Institute of Technology, Atlanta, GA, USA; ^49^Children's Healthcare of Atlanta, Atlanta, GA, USA; ^102^Emory University School of Medicine, Children's Healthcare of Atlanta, Atlanta, GA, USA

##### **Correspondence:** Daniel Whittingslow

Background : Juvenile idiopathic arthritis (JIA) is the most common chronic rheumatic condition of childhood. Up to 75% of JIA cases have temporomandibular joint (TMJ) inflammation. Untreated TMJ disease can cause pain and skeletal deformities. Early detection of TMJ involvement in JIA is difficult due to the variable symptomatology and low sensitivity of conventional imaging studies and physical exam. At present, magnetic resonance imaging (MRI) with contrast is the most sensitive diagnostic modality. However, it is expensive and has several potential complications. Thus, there is a need for a quantitative, objective metric that could screen patients with JIA for TMJ involvement. Acoustic emissions (AEs) are sounds produced during joint articulation. They are noninvasively measured. The purpose of this study was to classify TMJ involvement in patients with JIA using AEs.

Methods : This study included 15 patients (30 TMJs). Of them, 7 patients had JIA with TMJ involvement, and 8 had JIA without TMJ involvement. We used our custom-built headset to record AEs generated by the TMJs of each patient while performing open/close (OC) and medial/lateral (ML) movements jaws (Fig. 1 A). The b-values, a measure of the amplitude distribution of AEs, were calculated and compared to determine if they could be used to determine TMJ involvement.

Results : The qualitative time-domain analysis of the signals shows a more chaotic signal in affected TMJs. TMJs of patients with JIA without TMJ involvement produced a comparatively smoother signal (Fig. 1 B). The b-value was shown to be statistically different between the groups (ML exercise p=0.0059, OC exercise p=0.0009, Fig. 1C).

Conclusions : In this group of patients, AEs were different in children with JIA with TMJ involvement and without TMJ involvement. With further recruitment of subjects and refinement of this technique, assessment of the joint sounds may one day serve as a viable screening tool for TMJ involvement in JIA.

All research presented herein was approved by the Institutional Review Boards of Children’s Healthcare of Atlanta, Emory University, and the Georgia Institute of Technology.

**Fig. 1 (abstract A9).**
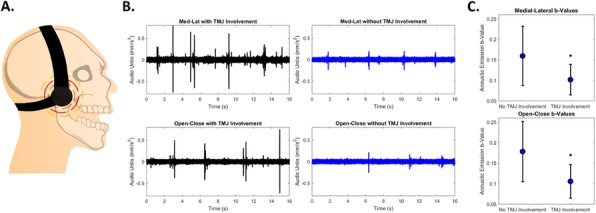
TMJ Sound recording from involved and non-involved jaws of patients with JIA. A. Diagram of recording setup. B. Time domain signal of TMJ sounds during ML movements (top) and OC movements (bottom). C. Comparison of b-values from the exercises

## A10 Working For Better Care And Treatment Of Paediatric-Onset Rheumatic And Musculoskeletal Diseases: Introducing The European Network For Children With Arthritis

### Simon Stones^101,102, 103, 104,^ , Anita van de Louw^102,112^, Mirjam Kepic^102,106^, Saskya Angevare^102,107,108^, Sammy Ainsworth^102,103,109^, Wendy Costello^102,110^, Anton Gruss^102,111^

#### ^101^ Fibromyalgia Action UK, Paisley, United Kingdom; ^102^European Network for Children with Arthritis, Geneva, Switzerland; ^103^RAiISE, Clitheroe, United Kingdom; ^104^EULAR Young PARE, Kilchberg, Switzerland; ^105^University of Leeds, School of Healthcare, Leeds, United Kingdom; ^106^Društvo za pomoč otrokom z imunskimi boleznimi, Ljubljana, Slovenia; ^107^KAISZ, Utrecht, Netherlands; ^108^Autoinflammatory Alliance, San Francisco, CA, USA; ^109^Alder Hey Children’s NHS Foundation Trust, Liverpool, United Kingdom; ^110^Irish Children’s Arthritis Network, Tipperary, Ireland; ^111^KOURIR, Paris, France; ^112^Jeugdreuma Vereniging Nederland, Apeldoorn, Netherlands

##### **Correspondence:** Simon Stones

Background : The European Network for Children with Arthritis (ENCA) is a pan-European network led for and by parents and young people with rheumatic and musculoskeletal diseases (RMDs). The activities of ENCA are delivered on a voluntary basis by parents and young people passionate about improving the care and treatment of children and young people (CYP) with RMDs. This is done through education, networking and empowerment in association with the Paediatric Rheumatology European Society (PReS), which represents paediatric rheumatology physicians, healthcare professionals and researchers. ENCA aims to facilitate the exchange and dissemination of information, knowledge and best practice with regards to paediatric RMDs, working in partnership with national patient organisations for CYP and families living with RMDs across Europe, and further afield. ENCA also aims to provide international awareness, engagement and research opportunities.

Methods : ENCA is managed by a board of seven elected members, all of whom are volunteers. ENCA hosts an annual conference in conjunction with the PReS conference, inviting attendance from national patient organisations. Aside from a newly refreshed website, ENCA hosts a closed Facebook and WhatsApp group for national patient organisation representatives, enabling real-time communication and the instant sharing of knowledge and best practice. ENCA hosts an annual ‘Fun with JIA challenge’, aimed at empowering CYP with RMDs to share their story; and has been involved in establishing a new international awareness campaign for 2019, titled ‘World young Rheumatic Disease (WORD) day’. In addition, ENCA cooperates with a number of international organisations with shared goals in rheumatology, representing the paediatric voice, including CARRA.

Results : Since its inception in 2002, ENCA has made advances in enhancing communication and shared learning between national patient organisations, by embedding the voice of CYP and families into research and advocacy activities. The ‘Fun with JIA challenge’ delivered since 2017 has provided CYP with RMDs across Europe with the opportunity to share their stories through video. In addition, ENCAs' cooperation with PReS has facilitated the planning and imminent launch of WORD day, taking place on March 18th, 2019. The need for a specific paediatric RMD awareness day was identified as a priority in light of the relatively limited attention that paediatric RMDs receive. It is anticipated that WORD day will raise parental and professional awareness about paediatric RMDs.

Conclusions : As parents and young people living with RMDs, ENCA has enabled individuals and national patient organisations to develop their knowledge in relation to paediatric RMDs. ENCA provides several opportunities for networking and the sharing of best practices between national patient organisations and professionals, through informal and formal activities focused on paediatric RMDs. International networking helps individuals and communities to navigate through challenges in a collective fashion to advance research and clinical care.

## A11 Trisomy 21 and Lung Disease in Systemic Onset Juvenile Idiopathic Arthritis

### Vivian Saper^214^, Guangbo Chen^214^, Gail Deustch^206^, R. Paul Guillerman^219^, Ann N.C. Leung^214^, Elizabeth Mellins^214^, 52 Case Reporters^3^, for the CARRA Registry investigators

#### ^214^Stanford University School of Medicine, Stanford, CA, USA; ^206^Seattle Children's Hospital, Seattle, WA, USA; ^219^Texas Children's Hospital, Houston, TX, USA; ^3^42 Contributing Institutions

##### **Correspondence:** Vivian Saper

Background : We collected cases with unusual lung disease occurring while on treatment with cytokine inhibitors of IL-1 or IL-6. While pleuritis is a known manifestation of lung disease in systemic onset juvenile idiopathic arthritis, parenchymal or pulmonary vascular disease is not. Coincident with the advent of these new medications, increasing reports of pulmonary involvement in sJIA appeared. Our initial study collected clinical, radiographic and pathologic data in association with the development of this incompletely characterized lung disease. Among the 61 included cases, 46 occurred during treatment with these cytokine inhibitors. Since Trisomy 21 occurs in sJIA in proportion to its occurrence in the general population^1^, confirmed by CARRA new registry data and Pharma CHILD registry, we were surprised to find six cases of children with Trisomy 21 among the 46 cases developing lung disease during treatment with these medications (‘pre-exposed’).

Methods : We conducted a retrospective observational case series including 61 children with sJIA and sJIA-like illness who developed lung disease between 2002 and 2017. To be included, cases had to have evidence substantiating the primary diagnosis, information at the time of noticing lung disease and either or both of a chest CT or lung biopsy or autopsy specimen available for independent expert review. Data was collected utilizing a RedCap database securely housed at Stanford University.

Results : A total of 72 cases were considered for inclusion. 61 fulfilled inclusion criteria and were included in the analytic set of 45 cases of sJIA and 16 cases of sJIA-like illness. Six cases were Trisomy 21 (2 sJIA-like and 4 sJIA) with five from the US and one from Europe. Features exhibited in the Trisomy 21 cases matched the other pre-exposed cases including the pathology in the spectrum of pulmonary alveolar proteinosis to endogenous lipoid pneumonia (PAP/ELP). Five of the cases had evidence of pulmonary vascular disease and/or clinical pulmonary hypertension. Clinical summary is shown in the table:

Conclusions : Treatment associated lung disease is found to occur in children with sJIA and sJIA-like illness with excessive frequency in children with sJIA and Trisomy 21 (OR > 50 compared to sJIA without Trisomy 21). After data close, two further cases of sJIA in Trisomy 21 have been brought to our attention; one is deceased. Features found and the consequences match the rest of those pre-exposed to cytokine inhibitor treatment including diffuse lung disease on CT, odd rash, acute erythematous digital clubbing, prodromal lymphopenia, pronounced eosinophilia and significant tocilizumab reaction. However, in the Trisomy 21 cases, hypoxia and abnormal chest auscultation at lung disease diagnosis were more common and MAS first occurred during lung disease. Caution in use of these medications in children with sJIA and Trisomy 21 is advised.

^1^ Alexander M, Petri H, Ding Y, Wandel C, Khwaja O, Foskett N. Morbidity and medication in a large population of individuals with Down syndrome compared to the general population. Dev Med Child Neurol 2016;58:246-54.

Acknowledgments: This work could not have been accomplished without the aid of the following organizations: The NIH’s National Institute of Arthritis and Musculoskeletal and Skin Diseases (NIAMS) & the Arthritis Foundation. We would also like to thank all participants and hospital sites that recruited patients for the CARRA Registry. IRB consent was obtained at each institution consistent with local requirements.

**Table 1 (abstract A11).** See text for description

Summary of clinical features associated with lung disease in Trisomy 21-sJIA cases					
	Trisomy 21			Pre-Exposed	
	sJIA-like	sJIA	PAP/ELP+^1^	Trisomy 21	Not Trisomy 21
Number of cases	2/6	4/6	5/5	6/6	40/55
Age at sJIA onset (years)	0.6, 2.5	0.5, 1.6, 3.2, 9.9	0.5, 0.6, 2.5, 3.2, 9.9	0.5 to 9.9	Median 2.3 (IQR:1.2-5.1)
Prior to lung disease					
MAS≧1 episode	0/2	0/4	0/5	0/6	29/40 (73%)
Period of disease quiescence on treatment^2^	0/2	1/4	0/5	1/6	18/40 (45%)
Atypical clinical findings occurring between 6 and 1 month prior to lung disease					
Atypical rash	2/2	2/4	3/5	4/6	23/40 (58%)
Acute clubbing	1/2	3/4	3/5	4/6	31/40(78%)
Lymphopenia <60% lower limit of normal^3^	1/1	3/4	3/4	4/5	17/36(47%)
Tocilizumab reaction	1/2	0/4	1/4	1/5	13/28(46%)
Peripheral eosinophilia w/in prior year^4^	1/2	2/4	2/5	3/6	17/39(44%)
Clinical features at lung disease					
Unexpected CT pattern(s) of diffuse lung disease^5^	0/1	4/4	4/5	4/5	35/39 (90%)
Hyper-enhancing lymph nodes on CT^6^	0/1	1/2	1/3	1/3	11/24 (46%)
Ferritin >1000ng/ml	1/1	2/4	2/4	3/5	14/27 (52%)
Overt MAS within prior 6 months	0/1	0/4	0/4	0/5	11/40 (28%)
Hypoxia	2/2	3/4	4/5	5/6^7^	14/40 (35%)^7^
Abnormal chest auscultation	2/2	3/4	4/5	5/6^7^	5/40 (13%)^7^
Viral infection	1/2	4/4	4/5	5/6^7^	11/40 (28%)^7^
Pulmonary hypertension or vascular disease^8^	1/2	4/4	4/5	5/6	17/40(43%)
Features during lung disease					
MAS	1/2	2/4	2/5	3/6	16/40(40%)
Alive at data close	1/2	2/4	2/5	3/6	28/40(70%)

^1^Pathology on lung tissue evaluation showed pulmonary alveolar proteinosis/endogenous lipoid pneumonia

^2^At least one period of ≧6 weeks with ferritin normal for age, no MAS, no pulse steroids and oral steroids zero to <0.15m/k/d. Arthritis allowed

^3^Van Gent et al Refined characterization and reference values of the pediatric T- and B-cell compartments. Clin Immunol 2009;133:95-107

^4^Peripheral eosinophilia in Trisomy 21 cases occurred within 4 months prior to lung disease

^5^Omits patterns of crazy paving only or predominantly ground glass opacities (often seen with PAP/ELP pathology)

^6^Evaluable on contrast enhanced CT

^7^Significant difference by odds ratio (95% CI): Hypoxia 9.3(1,87), Abnormal chest auscultation 35(3.4,364), Viral infection 13.2(1.4,126)

^8^Pulmonary vascular disease on lung tissue and/or clinical pulmonary hypertension

## A12 Changes in Total Body Fat and Body Mass Index among Children with Juvenile Dermatomyositis

### Amer Khojah^16^, Victoria Liu^316^, Gabrielle Morgan^16^, Chiang-Ching Huang^135^, Kaveh Ardalan^16^, Richard Shore^16^, Jackie Bellm^16^, Lauren M. Pachman^171^

#### ^16^Ann and Robert H. Lurie Children’s Hospital, Chicago, IL, USA; ^316^Wellesley College, Wellesley, MA, USA; ^135^Joseph J. Zilber School of Public Health, Milwaukee, WI, USA; ^171^Northwestern’s Feinberg School of Medicine, Chicago, IL, USA

##### **Correspondence:** Amer Khojah

Background : Despite the recent advances in the treatment of JDM, high dose glucocorticoids (GC) remain the main therapy to induce remission. Weight gain and cushingoid features are among the most common side effects of GC treatment. Excessive weight gain has a negative impact on children physical and psychological well-being. Studies of the natural history of GC associated weight gain in children are very limited, especially in JDM. Beside GC therapy, there are other possible mechanisms of obesity in JDM subjects such as: the lack of physical activity due to muscle weakness and metabolic changes from chronic inflammation. However, previous studies showed that untreated JDM subjects have lower weight and height than controls. The aim of this study is to measure the changes in BMI in a cohort of JDM subjects over 60 months and to examine the changes in body composition (fat vs lean body mass) by Dual-energy X-ray absorptiometry (DXA) scan.

Methods : An IRB approved chart review study (IRB# 2012-14858) was conducted at Lurie Children’s Hospital. We included all subjects between 2000 and 2017 with JDM who had at least 5 years of follow up data and DXA scan done during the study period. DXA was performed using GE-LUNAR iDXA bone densitometer. BMI and TBF (total body fat) percentile were calculated based on the CDC published BMI and TBF percentile charts. To study the natural history of weight gain and body fat changes we divided the data to 4 groups based on the duration between the date of first medication use and date of assessment (V0 = baseline, V1 > 1.5 years, V3= 1.51-3.49 years, V5 = 3.5-5 years).

Results : 96 subjects (78% female, 70% white, 44% P155/140 antibody, 33% MSA negative) were included. The mean duration of treatment was 0.95 +/- 0.4, 2.59+/- 0.4 and 4.42 +/- 0.5 years for V1, V3, and V5 respectively. Paired t-test showed a significant increase in the mean BMI percentile by 17.5 points (P 0.004) after the initiation of medical treatment followed by a gradual decrease over the study period (Fig 1A). TBF percentile did not change over the study period (Fig 1B). TBF in the last visit had a strong correlation with the V1 BMI, and V1 TBF percentile (correlation coefficients 0.63, 0.56 P <0.0001, 0.002 respectively). Interestingly, linear regression analysis showed a significant association (R2= 0.15, P 0.002, correlation coefficients 0.39) between the TBF percentile and muscle DAS (disease activity score) but not skin DAS.

Conclusions: After initiation of medical therapy, JDM subjects had higher BMI and TBF in comparison to historical healthy children, presumably from GC treatment. Although BMI percentile decreased over the duration of the study, the TBF percentile remained high through 60 months. This finding raises the concern that reduction in the BMI could reflect a drop in the lean body mass from muscle wasting rather than true fat loss. Furthermore, there was a significant correlation between initial muscle DAS and TBF percentile.

**Fig. 1 (abstract A12).**
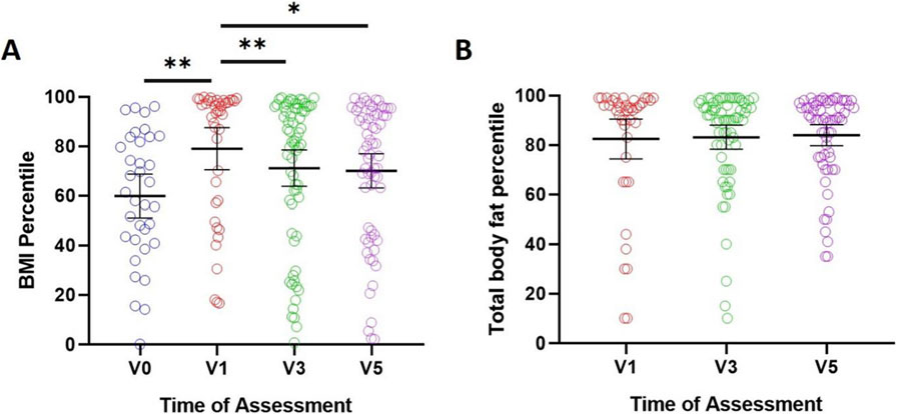
The changes in body mass index (BMI) and total body fat (TBF) percentile over the study duration. Mean BMI percentile increased significantly after the initiation of medical treatment followed by a gradual decrease over the study period. Total body fat percentile remained unchanged over the study period

## A13 Hormone and Adipokine Effects on cSLE Disease Activity During Puberty

### Kathleen O'Neil, Amy Rakestraw

#### Indiana University School of Medicine; Riley Hospital for Children at Indiana University Health, Indianapolis, IN, USA

##### **Correspondence:** Kathleen O'Neil

Background : A fundamental mystery of childhood systemic lupus erythematosus (cSLE) is why girls get the disease more often than boys and why cSLE seems more difficult to control during puberty. Several adipokines (fat cell-derived hormones) and sex-steroids follow the dichotomous incidence rates by sex seen in SLE during mid-childhood and puberty, so are of interest in identifying new targets for adjunctive approaches to modify disease activity.

Methods : We utilized a database and serum bank from a prospective observational study of children with pre-pubertal onset cSLE to examine the role of individual hormones and adipokines that change during puberty to determine if these changes are associated with variations in disease activity. Estradiol, leptin, adiponectin, prolactin, resistin, interferon-alpha, IP10 and MCP1 were measured in serum using commercial Bioplex assays. Serum visfatin (V) and testosterone (T) were measured by enzyme immunoassay. Time points of interest in pubertal progress were based on Tanner stage changes +/- one 3-monthly visit. cSLE disease activity was measured by SLEDAI (SLE Disease Activity Index) 2K. Flares used SELENA-SLEDAI definitions. Standard comparative statistics were used for analysis of differences across pubertal time periods. When measurement of all samples is complete, GEE will assess the contribution of each factor to flares/improvements over time.

Results : We measured 8 hormones, adipokines, and biomarkers of IFN activity in 53 serum samples of pubertal children with early onset cSLE enrolled in the SLE-Puberty cohort and examined the correlation between disease activity (SLEDAI-2K scores and flare rates) with Tanner stages of puberty. SLEDAI was higher during Tanner stages 2 and 3, when flare rates are also higher (p<0.008). Of 424 evaluable visits 118 (27%) were at Tanner transitions. Flare rates at transition visits did not differ from non-transition visits. Of the 115 flares, 38 (33%) occurred at puberty transitions. Of those, 34 (89.5%) were either at the initiation of puberty - the Tanner 1-2 transition, or mid-pubertal, the Tanner 2-3 transition (p=0.038). Only 4 were after Tanner 3. Measurement of estradiol, prolactin, adiponectin, resistin, leptin, IFN-alpha, IP10 and MCP1 in all samples is complete. T and V measurement is in progress in the last 200 samples. Biostatistical analysis to determine the relative contribution of each hormone/adipokine to SLEDAI and flare rates using generalized equalizing equations is pending the completion of analyte measurement this month.

Conclusions : The study confirms that puberty is a turbulent period for children with pre-pubertal onset SLE. The most critical phase begins with the earliest physical signs of puberty and returns to baseline after transition to Tanner 3, when menarche is imminent in girls and boys reach near-adult genital development and reproductive potential. The cohort is one of the largest of children with pre-pubertal onset SLE, and the largest with prospective longitudinal data. Early results in the first 36 children studied (2016) suggested adipokine production is related to disease activity; completion of this analysis will help define which mediators are most important and at which times during puberty.

The study used existing data and samples from a previous study. The Indiana University IRB determined the study was exempt from full board review. The original data and sample collection was reviewed and approved by the IRBs/REBs of the ten participating institutions.

Acknowledgments: The authors wish to acknowledge the ongoing Arthritis Foundation financial support of CARRA.

NIAMS R03AR52453, NIAID R56AI085258, the LFA Michael Jon Barlin Pediatric Lupus Grant,

CARRA-Arthritis Foundation 2018 Large Grant

## A14 Current Practices in the Management of TNFi failure in Juvenile Spondyloarthritis

### Melissa Oliver^198^, Pamela Weiss^56^, Robert Colbert^165^, Hemalatha Srinivasalu,^48^ for the CARRA juvenile spondyloarthropathy workgroup

#### ^198^Riley Hospital for Children, IU School of Medicine, Indianapolis, IN, USA; ^56^Children's Hospital of Philadelphia, Philadelphia, PA, USA; ^165^NIH/NIAMS, Bethesda, MD, USA; ^48^Children’s National Medical Center, Washington, DC, USA

##### **Correspondence:** Melissa Oliver

Background : Tumor necrosis factor inhibitors (TNFi) are effective and generally represent the first-line for biologic therapy in children with juvenile spondyloarthritis (JSpA). However, not all JSpA patients may respond well to TNFi initially (primary non-response) or respond well but may lose efficacy over time (secondary non-response). Children who do not respond to initial TNF inhibition are left with limited options. We aimed to assess the management of patients with JSpA who fail TNFi therapy.

Methods : An online survey was distributed to Childhood Arthritis and Rheumatology Research Alliance (CARRA) members of the JIA workgroup. Survey collected data on the number of JSpA patients who have failed TNFi therapy in practice, reasons for discontinuing anti-TNF therapy and other medications used afterward for the management of JSpA. The JSpA population included the following subtypes: enthesitis-related arthritis, psoriatic arthritis, undifferentiated spondyloarthritis, juvenile ankylosing spondylitis and reactive arthritis. Descriptive statistics were used to summarize the findings.

Results : The survey response rate was 36% (N= 169). As shown in Table 1, the majority of participants were pediatric rheumatologists (93%) from academic centers (91%). Many physicians have JSpA patients who failed TNFi therapy (63%). Sacroiliitis was the most important factor considered when assessing response to a TNFi. The most common reason for changing anti-TNF therapy was secondary non-response (72%). The most common reason for primary non-response was active sacroiliitis (45%). When assessing TNFi failure for sacroiliitis, many (65%) felt imaging of the sacroiliac joints was the most important aspect in their decision making. A majority try a second TNFi after initial TNFi failure (87%) and switch to another medication class after 2 TNFi agents have failed (62%). Other agents used after TNFi failure were abatacept, tocilizumab, ustekinumab, and secukinumab.

Conclusions : More than half of pediatric rheumatologists have at least one JSpA patient who has failed TNFi therapy. The majority have failed because of secondary non-response. Sacroiliitis is an important but challenging aspect to manage for patients with JSpA.

Acknowledgements: The authors wish to acknowledge the ongoing Arthritis Foundation financial support of CARRA. This project is funded by a CARRA-Arthritis Foundation grant.

**Table 1 (abstract A14).** Characteristics of Physician Management of JSpA Patients (n=60)

	N (%)
Practice Location	
Academic	55 (91)
Private	4 (7)
Years in practice	
<5 years	18 (30)
5-10 years	17 (28)
11-15 years	9 (15)
> 15 years	16 (27)
Training	
Pediatric Rheumatologist	56 (93)
Med/Peds Rheumatologist	3 (5)
Other	1 (2)
Number of JSpA patients seen annually	
< 5 patients	5 (8)
5-10 patients	16 (27)
>10 patients	39 (65)
Duration before deeming treatment failure	
2 months	4 (7)
3 months	36 (60)
6 months	14 (23)
Have JSpA patients who failed TNFi therapy in last 2 yrs	38 (63)
Have switched back to same TNFi after failing	10 (17)
Reasons for change anti-TNF therapy:	
Secondary non-response	43 (72)
Primary non-response	35 (58)
Intolerance	29 (48)
Concomitant disease	28 (47)
Chronic pain	15 (25)
Reasons for primary non-response	
Arthritis (Sacroiliac joints)	27 (45)
Arthritis (peripheral)	16 (27)
Enthesitis	16 (27)
Pain	2 (1)
Number of anti-TNF agents (total) tried before switching to another class	
1	1 (1.5)
2	37 (62)
3	21(35)
>3	1 (1.5)
Other medications used after TNFi failure	
Abatacept	24 (40)
Tocilizumab	21 (35)
Ustekinumab	17 (28)
Secukinumab	16 (27)
Tofacitinib	8 (13)
Rituximab	2 (3)
Other: Apremilast (1), Methotrexate (1), Steroids (1)	

## A15 Differences in Chromatin Architecture in Treatment Naïve Pediatric Lupus Patients

### Joyce Hui-Yuen^68^, Frank Jenkins^104^, Kaiyu Jiang^217^, James Jarvis^217^

#### ^68^Cohen Children's Medical Center, Queens, NY, USA; ^104^Feinstein Institute for Medical Research, Manhasset, NY, USA; ^217^State University of New York at Buffalo, Buffalo, NY, USA

##### **Correspondence:** Joyce Hui-Yuen

Background : Systemic lupus erythematosus (SLE) is a complex disease possibly triggered by gene-environment interactions. Over 20% of SLE patients have symptoms before 18 years, making SLE the 2nd most common pediatric rheumatic disease. We have shown that most of the SLE-associated haplotypes encompass genomic regions enriched for epigenetic marks associated with enhancer function in neutrophils, and T/B cells, suggesting that genetic risk is exerted through altered gene regulation. Data remain scarce on how epigenetic variance contributes to disease risk in pediatric SLE. We sought to identify differences in chromatin architecture in treatment-naïve pediatric lupus (pSLE) patients compared to healthy children.

Methods : We used the assay for transposase-accessible chromatin-sequencing (ATACseq) to survey open chromatin in 8 treatment-naïve pSLE patients and 5 healthy patients (HP). We investigated whether regions of open chromatin unique to pSLE patients might demonstrate enrichment for specific transcriptional regulators, using standard computational approaches to identify unique peaks and a false discovery rate of <0.05.

Results : The mean age of onset was 13.75 (range 7-17) years in pSLE patients; 3 out of 8 patients were male. pSLE patients were 50% African American, 38% Caucasian, and 12% Asian, with all but 1 patient identifying Hispanic. All patients had a lupus disease activity score of at least 4. Differential peak summit analysis identified 38 uniquely accessible sites in pSLE patients. Further analyses of these open regions revealed that 46-60% of the peaks seen only in pSLE patients are located more than 100kb from the nearest transcription start site, implying that many transcription factors (TFs) may be acting on distal enhancers to regulate transcription. In addition, gene set and TF enrichment analyses identified 35 genes and 331 TFs that may be accessible in pSLE patients but not HP. Subsequent motif enrichment showed differential accessibility of the TFs STAT2 and BCL11A in the 38 uniquely accessible regions in pSLE patients. Interestingly, STAT2 expression is known to be upregulated after binding of interferon-alpha to its receptor on the cell surface.

Conclusions : Patterns of chromatin accessibility suggest important roles for chromatin regulators (e.g., STAT2 and BCL11A) in treatment-naïve pSLE.

Acknowledgements: We wish to acknowledge the ongoing Arthritis Foundation financial support of CARRA. This project is supported by a CARRA – Arthritis Foundation grant.

## A16 Differences in Clinical Manifestations in Pre-pubertal onset SLE across different Racial/Ethnic Groups

### Martha Rodriguez, Kathleen O'Neil

#### Indiana University School of Medicine, Riley Hospital for Children, Indianapolis, IN, USA

##### **Correspondence:** Martha Rodriguez

Background : Systemic lupus erythematosus (SLE) is a remarkably complex and heterogeneous disease. Age at onset, sex, and race/ethnicity are important factors that impact disease outcome (1). Preliminary analysis from the Lupus-Puberty cohort, which aims to examine the impact of puberty-associated hormone and adipokine changes on SLE, suggests disease activity in juvenile lupus (jSLE) rises during puberty in children with pre-pubertal onset lupus (2). This abstract examines how race/ethnicity impacts clinical manifestations, disease activity and flare rates in pre-pubertal onset jSLE followed through puberty.

Methods : In a prospective multicenter (10 CARRA sites) longitudinal observational study we collected data from 63 subjects (51 Females, 12 Males) with jSLE prior to the onset of puberty. Comprehensive medical history, physical examination findings, Tanner stage, laboratory assessments, SLEDAI-2K activity scores and MD-global assessments were collected every 3 months. Flares were determined by SELENA-SLEDAI Flare Index. Standard descriptive statistics were applied; differences reported if p<0.05

Results : The average duration of study involvement was 21 months. The cohort included 32% Caucasian, 24% African American (AA), 20% Asian, 19% Hispanic and 0.05% Native American children. Among ethnic groups, renal disease was more frequent in Hispanics 58%, followed by AA 53%. Class IV SLE nephritis was the most common pathology in Hispanics 33% followed by African Americans 27% and Caucasians 15%. Class V was more common in Asians seen in 23%. Arthritis and discoid lupus were more frequent in African Americans (67% and 27% respectively). The prevalence of CNS disease was highest in AA (20%). Lupus headache was the most common CNS manifestation (50%) followed by cognitive dysfunction and seizures (38% each). Cardiopulmonary manifestations were most frequent in Hispanics (33%). Pleuritis accounted for 13% and pericarditis 11%. Cytopenias were more common in Caucasian subjects (65%). SSA/SSB were more common in Asians (69%); antiphospholipid antibodies were more frequent in Hispanics (75%). SLEDAI median scores were highest in AA (11+/-4.8) followed by Hispanics (7.5+/-6.2, Caucasians (6+/-6.07), and lowest in Asians (4+/-3.9) and Native Americans (4+/-5.7; n=3). Flares of any kind were more common in AA 29% (113 visits) and Caucasians 29% (121 visits) followed by Hispanics 28 % (108 visits) and Asians 28% (65 visits). Native Americans had the lowest incidence of flare at 11% (27 visits). Mild-Moderate flares (MMF) were most frequent in Caucasians (24%), followed by Hispanics (22%) and AA (18%). Severe flare (SF) incidence was highest in AA (11%) followed by Hispanics 6% and Caucasians 5%. No SF were identified in Asians or Native Americans.

Conclusions : AA and Hispanic children have higher SLEDAI and more severe flares than Caucasian and Asian children with jSLE. Disease onset, puberty and race/ethnicity are determining factors in prognosis in SLE. Treatment algorithms based on these factors may help to improve long-term outcomes in childhood SLE.

Acknowledgments: This project is supported by a CARRA – Arthritis Foundation grant. The authors wish to acknowledge the ongoing Arthritis Foundation financial support of CARRA.

## A17 Renal Outcomes in Pediatric Anti-Neutrophil Cytoplasmic Antibody Associated Vasculitis in the First 24-Months

### Kimberly Morishita^261^, Audrea Chen^261^, Cherry Mammen^261^, Damien Noone^292^, Rae S.M. Yeung^292^, Susanne Benseler^263^, Merav Heshin-Bekenstein^267^, Roberta Berard^317^, Sirirat Charuvanij^140^, Melissa Elder^212^, Adam Huber^77^, Karen James^297^, Marisa Klein-Gitelman^13^, Mikhail Kostik^204^, Raju Khubchandani^134^, Tzielan Lee^214^, Susan Nielsen^196^, Kathleen O'Neil^129^, Kathryn Phillippi^7^, Ricardo Russo^177^, Susan Shenoi^208^, Vidya Sivaraman^155^, Maria Yee^225^, David Cabral^261^

#### ^261^University of British Columbia and BC Children's Hospital, Vancouver, BC, Canada; ^292^University of Toronto and the Hospital for Sick Children, Toronto, ON, Canada; ^263^University of Calgary and Alberta Children's Hospital, Calgary, AB, Canada; ^267^University of California San Francisco, San Francisco, CA, USA; ^317^Western University and Children's Hospital London Health Sciences Centre, London, ON, Canada; ^140^Mahidol University and Siriraj Hospital, Bangkok, Thailand; ^212^Shands Children's Hospital, College of Medicine, University of Florida, Gainesville, FL, USA; ^77^Dalhousie University and IWK Health Centre, Halifax, NS, Canada; ^297^University of Utah, Salt Lake City, UT, USA; ^13^Ann & Robert H. Lurie Children’s Hospital of Chicago, Chicago, IL, USA; ^204^Saint Petersburg State Pediatric Medical University, Saint Petersburg, Russian Federation; ^134^Jaslok Hospital and Research Centre, Mumbai, India; ^214^Stanford University School of Medicine, Stanford, CA, USA; ^196^Rigshospitalet, Copenhagen, Denmark; ^129^Indiana University School of Medicine, Riley Hospital for Children, Indianapolis, IN, USA; ^7^Akron Children’s Hospital, Akron, OH, USA; ^177^Paediatric Hospital Dr. Juan P. Garrahan, Buenos Aires, Argentina; ^208^Seattle Children's Hospital, Seattle, WA, USA; ^155^Nationwide Children's Hospital, Columbus, OH, USA; ^225^The Children’s Hospital at Montefiore and Albert Einstein College of Medicine, Bronx, NY, USA

##### **Correspondence:** Kimberly Morishita

Background : Renal disease is the most common manifestation of pediatric anti-neutrophil cytoplasmic antibody (ANCA) associated vasculitis (AAV). Renal outcomes in pediatric AAV have not been well studied. The aim of this project was to describe renal outcomes in the first 24-months of disease.

Methods : PedVas is a multi-center, CARRA-endorsed international study that is currently collecting clinical and biological data from children with chronic vasculitis. Patients from PedVas included in this study had: granulomatosis with polyangiitis (GPA) or microscopic polyangiitis (MPA) PLUS renal disease; were < 18 years of age at time of diagnosis (TOD) and had at least 12 month follow up data. Renal disease was defined as biopsy confirmed pauci-immune glomerulonephritis OR dialysis dependence at TOD. GFR was estimated using the bedside Schwartz formula. GFR based outcomes were reported as proportion of children with normal GFR (>90 ml/min/1.73m2), mildly reduced (MildR) (60-89 ml/min/1.73m2), moderately reduced (ModR) (30-59 ml/min/1.73m2), severely reduced (SevR) (15-29 ml/min/1.73m2), and renal failure (RF) (<15 ml/min/1.73m2, or requirement of dialysis). Damage was assessed using the pediatric vasculitis damage index (pVDI).

Results : 124 patients (69% female and 79% with GPA) met inclusion criteria. Median age at TOD was 13.8 years (range 3.3 – 17.8 years). For available ethnicity data, 56% were Caucasian, 11% Hispanic, 8% East Indian/South Asian, and 25% other (Asian, Black, Aboriginal, Mixed, or Other). Cyclophosphamide (CYC) (85%) and/or rituximab (RTX) (18%) were used as primary induction treatments in 94% of patients (9% received both CYC and RTX). 38 patients (31%) received plasmapheresis in combination with CYC and/or RTX. At TOD, 35% of patients had a normal GFR. The remaining patients had GFRs as follows: 8% MildR, 20% ModR, 12% SevR, and 25% RF. At 12-months, 34% of patients had a normal GFR, 30% MildR, 14% ModR, 6% SevR, and 17% RF with 4 patients receiving renal transplants. Of 48 patients with complete 24-month visit data, 40% had normal GFR, 33% MildR, 10% ModR, 4% SevR, 13% RF or were transplanted (2 additional patients). At 12-months, 71% of patients had 1 or more damage item scored on the pVDI. 50% had scores of 2 or more. At 24-months, 56% had scores of 1 or more, 41% had scores of 2 or more. The most common renal damage item scored was proteinuria (37% and 21% at 12- and 24- months respectively). Of the 31 patients that had RF at TOD, 17 (55%) remained in RF or were transplanted and only 1 patient (3%) had a normal GFR at last follow up. No patients died.

Conclusions : More than half of children with AAV associated renal disease have moderately reduced renal function or worse at diagnosis. At 12- and 24-month follow up, two-thirds of patients continue to have reduced renal function despite 94% of patients receiving CYC and/or RTX. Patients who present with RF are unlikely to recover normal renal function. More than half of patients with renal disease will have evidence of damage by 12 months. Further study of predictors of renal outcomes is needed to inform management strategies at diagnosis and ultimately improve outcomes.

This study received IRB approval at all sites.

Acknowledgement: This study was jointly supported by a CARRA - Arthritis Foundation Grant and the CIHR-funded Pediatric Vasculitis (PedVas) Initiative. The authors wish to acknowledge the ongoing Arthritis Foundation financial support of CARRA.

## A18 Immunogenicity of Pneumococcal Vaccination and Impact on Nasopharyngeal Pneumococcus Colonization in patients with Childhood Onset Systemic Lupus Erythematosus (cSLE)

### Fatima Barbar-Smiley, Stacy Ardoin, Chack-Yung Yu, Vidya Sivaraman, Cagri Yildirim-Toruner, Octavio Ramilo

#### Nationwide Children's Hospital, Columbus, OH, USA

##### **Correspondence:** Fatima Barbar-Smiley

Background : Streptococcus pneumoniae (S. pneumoniae) may lead to severe life-threatening infections in both the general and immunocompromised population. Patients diagnosed with Systemic Lupus Erythematosus (SLE) are five times more likely to acquire invasive pneumococcal disease compared to healthy controls. There is a known association between nasopharyngeal (NP) pneumococcal colonization (NPcol) and increased risk of infection with S. pneumoniae. Therefore, CDC recommends that patients with childhood onset SLE (cSLE) should receive both PCV13 and PPSV23 pneumococcal vaccines. Vaccine effect on pneumococcal NP colonization (VE-col) is suggested as a surrogate marker for protective vaccine effect against invasive pneumococcal infections, especially in young children. To date, no studies have examined the rate of NPcol in cSLE and how it correlates with vaccine immunity in this population. In our pilot study, we are assessing the rate and density of pneumococcal nasopharyngeal colonization following receipt of recommended pneumococcal vaccination in cSLE patients compared with age-matched healthy controls.

Methods : Prospective, observational, case-control study of cSLE patients who have received their recommended pneumococcal vaccines and healthy age matched controls. Study was approved by local IRB. Participants were consented, study questionnaire provided, then NP swab samples collected and analyzed for quantitative pneumococcal PCR. The positive samples are saved for future study of specific serotypes. Rates of positive NP swabs are compared using chi-square or Fisher’s exact tests. A multivariable logistic regression model will be constructed to determine whether cSLE status is associated with higher likelihood of positive NP swabs while adjusting for possible confounders.

Results : This study is still ongoing in recruitment stage, with a goal to recruit total of 40 cSLE patients and 40 controls. Currently recruited 16 cSLE patients of median age 17 yrs (9-23), 13 (81%) females, 8 (50.0%) White, 7 (44 %) African American, and 1 (6 %) Asian, 8 (50 %) cSLE patients had Lupus Nephritis. 7 controls have median age 16 yrs (9-24), 6 (86 %) females, 6 (86 %) white, 1(14 %) Asian. NP PCR results: 1/16 (6%) positive swab in cSLE and 0/7 positive control NP swab.

Conclusions : Preliminary finding of only one positive NP swab sample is surprising as we have anticipated that patients with cSLE will have higher positive rates of NP swabs compared to healthy controls. Study is still ongoing and final results may vary. In the future, we plan to examine pneumococcal serotypes in positive samples to determine if the colonization is related to vaccine-related subtypes. Results of this study will help us better understand indirect markers for immune response to pneumococcal vaccination and to explore factors that may impact the response to pneumococcal vaccine in childhood lupus.

Study was approved by the local institutional review board (IRB).

Acknowledgement: This research study is supported by Childhood Arthritis and Rheumatology research Alliance- Arthritis Foundation small grant award. The authors wish to acknowledge the ongoing Arthritis Foundation financial support of CARRA. 

## A19 LN-Autoantibodies: a Midwest Pediatric Nephrology Consortium Study of Pediatric-onset Lupus Nephritis

### Scott Wenderfer^23^, Jessica Greco^152^, Thandiwe Jere^23^, Sherene Mason^242^, Larry Greenbaum^101^, Mahmoud Kallash^152^

#### ^152^Nationwide Children's Hospital, Columbus, OH, USA; ^23^Baylor College of Medicine, Houston, TX, USA; ^242^UConn School of Medicine, Connecticut Children's Medical Center, Hartford, CT, USA; ^101^Emory University and Children's Healthcare of Atlanta, Atlanta, GA, USA

##### **Correspondence:** Scott Wenderfer

Background : There remains a need for non-invasive biomarkers of nephritis in childhood-onset systemic lupus erythematosus (cSLE), both to predict nephritis and to monitor disease activity. Abnormal proteinuria and hematuria are non-specific and unable to distinguish between active disease and renal scarring. Anti-double stranded DNA antibodies (dsDNA Ab) and complement components C3 and C4 are neither sufficiently sensitive nor specific. Glomerular filtration rate (GFR) falls only after the kidney is significantly damaged, so detectable changes are identified too late to successfully intervene. Repeat biopsies can be helpful but are too invasive for routine monitoring.

Methods : Blood, urine, and saliva samples were collected from 36 cSLE patients at onset of lupus nephritis (L+N+) before initiation of immunosuppression (corticosteroids allowed). Samples were also collected from three control groups: children with active SLE without active nephritis (L+N-), children with inactive SLE (L-N-), and healthy children without lupus and without nephritis (HC). A second set of bio-samples were collected 6 months into induction therapy for 81% of the L+N+ cohort in order to test for treatment effects. GFR was either measured by 24-hour urine or estimated using Schwartz formula. Data was available for 41 renal biopsies paired with bio-samples from 33 cSLE patients. Enrollment in the LN-Autoantibodies study is ongoing and open to all interested CARRA investigators.

Results : The ages, male:female ratios, and ethnic/racial distributions were similar between the L+N+ cohort and controls (Table 1). Mean SLEDAI scores in non-renal SLE patients enrolled at disease onset or flare (L+N-) indicated less active lupus than L+N+ subjects. Repeat samples in L+N+ cohort after 6 months showed an overall good response to induction therapy. Three L+N+ patients were lost to follow-up and one developed end stage kidney disease requiring chronic dialysis. Anti-dsDNA Ab titer correlated with SLEDAI score (r=0.46, p<0.001) and inversely with serum C3 levels (r=-0.45, p<0.001), but failed to distinguish between L+N+ and L+N- cohorts. C3 levels and GFR similarly failed to discriminate. Renal SLEDAI score was not always zero in L+N- or L-N-. On baseline kidney biopsy, 4 L+N+ subjects had class I or II, 15 had III or IV, 7 had pure V, and 7 had mixed class. Biopsies had a median activity index (AI) of 2.5 (range 0-17) and median chronicity index (CI) of 0 (range 0-8). There is a positive correlation between AI and Anti-dsDNA ab titers (r=0.64, p<0.001).

Conclusions : There remains a need for new biomarkers for diagnosis and management of nephritis in cSLE. Enrollment of non-renal cSLE patients with high disease activity has been a challenge. Collaboration with CARRA to obtain bio-samples for cSLE patients enrolled in the CARRA registry would help generate a more robust cohort for future biomarker discovery/validation.

The study was performed with IRB approval centrally at BCM (H-35050) and at all participating sites.

**Table 1 (abstract A19).** Demographics and Lab Results by Group

	L+N+		L+N-	L-N-	HC
	baseline	6 month	baseline	baseline	Baseline
Age	14.5 ± 2.7	15.2 ± 2.9	14.4 ± 3.0	15.8 ± 2.8	15.2 ± 1.9
Gender (F : M Ratio)	4.1 : 1	4.5 : 1	5.8 : 1	4.7 : 1	30 : 0
Ethnicity	**N (%)**				
Hispanic	18 (50)	16 (61)	14 (52)	17 (50)	14 (47)
Black	14 (39)	7 (27)	9 (33)	10 (29)	4 (13)
Caucasian	1 (3)	1 (4)	1 (4)	5 (15)	11 (37)
Biracial	2 (6)	2 (8)	0	0	0
Asian	1 (3)	0	3 (11)	2 (6)	1 (3)
SLEDAI	20.3 ± 7.7	6.7 ± 5.6	7.4 ± 4.7	2.4 ± 2.8	0
Renal SLEDAI	10.7 ± 5.1	4.0 ± 4.3	0.4 ± 1.3	0.8 ± 2.2	0
GFR (mL/min/1.73m^2^)	96 ± 42	111 ± 39	116 ± 26	107 ± 18	
Low C3 (+)	28 (78%)	6 (27%)	16 (59%)	2 (6%)	
C3 (mg/dL)	53 ± 36	100 ± 40	75 ± 36	112 ± 22	
Anti-dsDNA (+)	25 (69%)	13 (50%)	18 (67%)	13 (38%)	
dsDNA titer (median)	1:1280	0	1:80	0	
dsDNA titer (IQR)	3 – 1:1280	0 – 1:40	0 – 1:1280	0 - 1:40	

## A20 Understanding Langerhans Cell ADAM17 Levels in Systemic Lupus Erythematosus: Potential Contributor to Photosensitivity

### Keila Veiga^120^, Noa Shwartz^120^, Thomas Li^120^, William Shipman^120^, Dragos Dasoveanu^120^, Yong Liu^314^, Carl Blobel^120^, Niroshana Ananadasapathy^314^, Henry Lee^120^, Theresa Lu^120^

#### ^120^Hospital for Special Surgery, New York, NY, USA; ^314^Weill Cornell Graduate School of Medical Sciences, New York, NY, USA

##### **Correspondence:** Keila Veiga

Background : Photosensitivity resulting in inflammatory skin lesions is a hallmark of cutaneous lupus and is highly prevalent in systemic lupus erythematosus (SLE). These lesions can be disfiguring and have a negative impact on patients’ quality of life. Improved understanding of the basis of photosensitivity is critical in order to develop better treatment. Prior research by our lab has shown that ADAM17, a metalloprotease found on Langerhans cells (LCs), is activated by ultraviolet radiation (UVR) and is critical for limiting UVR-induced keratinocyte apoptosis and skin inflammation through cleavage and activation of epidermal growth factor receptor (EGFR) ligands. Two photosensitive lupus models showed reduced Langerhans cell ADAM17 expression, suggesting that photosensitivity is at least in part due to dysfunctional LCs that are unable to protect the skin. In this prior study, EGFR phosphorylation and activation were also found to be reduced in non-lesional, non-sun exposed skin samples collected from patients with lupus nephritis, suggesting that LCs are dysfunctional in human SLE as well. Here, we propose a cross-sectional study to evaluate the hypothesis that Langerhans cell ADAM17 expression and activity are reduced in the epidermis of patients with SLE, which contributes to photosensitivity. Our secondary hypothesis will address whether reduced ADAM17 expression and increased photosensitivity is associated with lupus disease activity and severity. As part of our secondary hypothesis, we are also looking to correlate reduced ADAM 17 levels with increased type I interferon in SLE.

Methods : We will collect punch biopsies of non-lesional, non-sun exposed skin from 10 patients who meet ACR/SLICC criteria for SLE as well as 5 healthy controls, at a single study visit. We will perform flow cytometry on Langerhans cells isolated from these biopsies to assess ADAM17 expression and activity. We will also collect the SLE Disease Activity Index (SLEDAI) score and we will evaluate the correlation between ADAM17 activity with these standard measures of disease activity. Lastly, we will perform RNA sequencing to evaluate the interferon signature in these samples and correlate interferon expression with ADAM17 levels.

Results : This study is currently ongoing, and preliminary results are expected within the next several months.

Conclusions : If ADAM17 expression is reduced in the skin of SLE nephritis patients in comparison to controls, this may indicate a role of ADAM17 in SLE photosensitivity. These findings could lead to improved treatments for the photosensitive rash seen in patients with lupus.

This study was approved by the Hospital for Special Surgery IRB.

## A21 Validation of the 1997 American College of Rheumatology (ACR) classification criteria, 2012 Systemic Lupus International Collaborating Clinics (SLICC) criteria, and the 2017 Weighted Criteria for pediatric Systemic Lupus Erythematosus (SLE)

### Meiqian Ma, Joyce Hui-Yuen, Barbara Anne Eberhard, Jane Cerise

#### Cohen Children's Medical Center/Northwell Health, Queens, NY, USA

##### **Correspondence:** Meiqian Ma

Background : Different classification criteria for systemic lupus erythematosus (SLE) have been proposed over many years. The most widely used and accepted criteria have been the 1997 ACR criteria. In 2012, the SLICC criteria were published in an attempt to improve clinical relevance of SLE criteria and address concerns with the 1997 ACR criteria. In 2017, a new candidate weighted criteria were proposed at the ACR meeting. Our aim was to validate the 1997 ACR, 2012 SLICC, and 2017 new weighted criteria for pediatric-onset SLE.

Methods : Retrospective chart review of patients with a clinical diagnosis of SLE diagnosed before the age of 19 years at our tertiary care center between 2002 and 2017. The ACR, SLICC, and new weighted classification criteria were applied to these patients. We excluded patients diagnosed at another center and those for whom no records were available for review. All criteria sets were compared against a gold standard of physician diagnosis. Autoimmune controls were defined as patients who were referred for serologies positive for ANA but who did not fulfill criteria for diagnosis of SLE at the initial visit, or were diagnosed with another autoimmune disease. Our collaborating sites, Children’s Hospital at Montefiore, Children’s Hospital at NYU Langone, and Columbia University Medical Center are in the process of collecting and entering data according to our inclusion/exclusion criteria.

Results : There were 156 patients (81% female) who were diagnosed with SLE over the past 15 years. The mean age at diagnosis was 13±2.7 years, with 24% Asian, 28% Black, 26% Caucasian, and 22% Other; 24% also identified as Hispanic. The sensitivity for the 1997 ACR criteria was 90.3 (CI: 0.84-0.95) and specificity was 95.9 (CI: 0.90-0.99). The sensitivity for the 2012 SLICC criteria was 99.3 (CI: 0.96-0.99) and specificity was 95.7 (CI: 0.90-0.99). The sensitivity and specificity for the 2017 weighted criteria are currently under analysis.

Conclusions : The 2012 SLICC criteria were more sensitive than, but with similar specificity as, the 1997 ACR criteria. This finding is comparable with the observed statistics seen in the adult population. We do not currently have the sensitivity and specificity for the new weighted criteria, but will have results by the time of the meeting.

This study was approved by the Northwell Health Internal Review Board.

Acknowledgements: This work is funded in part by a Fellow Grant from the Childhood Arthritis and Rheumatology Research Alliance – Arthritis Foundation awarded to Dr. Meiqian Ma. The authors wish to acknowledge CARRA, and the ongoing Arthritis Foundation financial support of CARRA.

## A22 Transition of Pediatric Lupus Patients to Adult Care: A Three-year Follow-Up; Outcomes from a Single Center

### Pauline Yi^268^, Hana Conlon^73^, Mridula Watt^268^, Imundo Lisa^73^

#### ^268^University of California, Los Angeles, Los Angeles, CA, USA; ^73^Columbia University, New York, NY, USA

##### **Correspondence:** Pauline Yi

Background : Loss of continuity when transitioning from pediatric to adult care for patients with childhood onset lupus (cSLE) results in poor outcomes. In this retrospective study, we evaluated the three-year post transition outcomes of 50 cSLE patients (onset ≤ 18 years), of low socioeconomic background in a single urban academic center, who transferred from pediatric to adult care with and without a structured protocol.

Methods : The transition group consisted of 30 cSLE patients who transitioned through a structured protocol (Hui-Yuen JS, et.al . Transitioning Lupus Patients from Pediatric to Adult Rheumatology #1399; Arth and Rheum 68:S10 Oct. 2016) into the adult lupus clinic, including 12 patients from the original abstract. We identified 20 cSLE patients as the traditional group who transferred without a structured protocol after 2010. Data including socio-demographics, SLE manifestations, SLEDAI and SLICC scores, and non-adherence was collected from the last pediatric and adult visits (January 2018).

Results : There were no significant differences in demographic and SLE characteristics between the groups. The age at transition in the protocol was 20.07 vs 20.75 years (p <0.0001). Although not statistically significant, the protocol group transferred with higher disease activity scores (5.3 vs 4.3, SD =5.1 p=0.4499) (Table 1). The most common organ involvement was the kidney. In the protocol cohort, 84% were in complete renal remission with 11% on HD compared to 57% and 29% in the non-protocol population. Medication non-adherence was defined by physician documentation or self-reported patient history. Follow up visit non-adherence was recorded as ≥ 2 missed consecutive appointments. Both measures were found to be lower in the non-protocol group (40% vs 55%, 60 vs 20%).

Conclusions: The structured transition protocol was associated with decreased time to first appointment with the adult provider and better attendance of follow-up visits. Our study also found better medication adherence in the structured transition group. Although counter to current beliefs, it is possible that higher disease activity at time of transition also contributed to improved appointment and medication adherence. However, there was no evidence of improved medical outcomes between two cohorts. This retrospective study is limited by the small sample size and the many variables affecting outcome. A long term prospective study is warranted to evaluate the impact of structured transition on cSLE outcomes.

This Study was approved by the Columbia University Internal Review Board.

**Table 1 (abstract A22).**
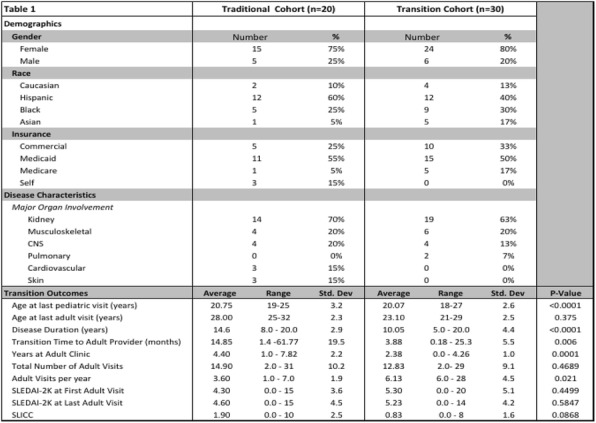
Demographics and Transition Outcomes

Demographics and transition outcomes of both groups - protocol and non-protocol

## A23 Differential Expression of Bone Morphogenetic Protein Antagonists and Chondrocyte Maturation Markers Provide Insight into the Role that Fibroblast-like Synoviocytes from Patients with Juvenile Idiopathic Arthritis Play in Growth Disturbances within the Joint

### Anne Marie Brescia^157^, Megan Simonds^157^, Amanda Schlefman^157^, Kathleen Sullivan^55^, Carlos Rose^157^

#### ^157^Nemours/ Alfred I. duPont Hospital for Children, Wilmington, DE, USA; ^55^Children's Hospital of Philadelphia, Philadelphia, PA, USA

##### **Correspondence:** Anne Marie Brescia

Background : Our purpose was to examine the role fibroblast-like synoviocytes (FLS) play in the bony overgrowth seen in Juvenile Idiopathic Arthritis (JIA) patients. FLS may play a direct role in endochondral bone formation (EBF) or influence growth plate chondrocytes (Ch) in joints. BMP antagonists regulate signaling in both FLS and Ch by binding to BMP ligands, but their effects on cell differentiation in JIA is currently unknown. To examine autocrine signaling of FLS, we treated cells from patients with JIA (JFLS) with BMP4 and measured protein expression of BMP antagonists gremlin (GREM), chordin (CHRD), noggin (NOG), and follistatin (FST), as well as Ch differentiation markers collagen II (COL2) and collagen X (COLX). Paracrine signaling was studied by culturing Ch in conditioned media from JFLS and measuring protein levels of the above proteins.

Methods : Cell media supernatants were collected at intervals over 24h from three JFLS treated with recombinant BMP4 and three JFLS in normal media. Media supernatants were also collected at intervals from Ch cells grown in conditioned media from JFLS (Ch-JFLS) and Ch cells grown in normal media. ELISAs were performed and expression ratios, standard errors, and p-values were calculated.

Results : Exogenous BMP4 causes decreases in NOG and FST in JFLS culture when compared to untreated (Fig 1A). JFLS conditioned media caused an increase of CHRD and decrease of NOG in Ch cultures when compared to untreated (Fig 1B). BMP4 addition caused COL2, a chondrocyte proliferation marker, to decrease and COLX, a chondrocyte hypertrophy marker, to increase in JFLS compared to untreated (Fig 1C). Interestingly, COLX decreases when Ch are cultured in JFLS conditioned media while there was no significant change in COL2 (Fig 1D).

Conclusions : Exogenous BMP4 promotes hypertrophy in JFLS but factors in FLS-conditioned media delay maturation of Ch. Differential expression of BMP antagonists support this conclusion. Despite the high affinity that FST and NOG have for BMP4, exogenous presence of the ligand promotes hypertrophy in BMP4 treated JFLS while Ch cultured in JFLS conditioned media favor CHRD. CHRD is an effective repressor of Ch hypertrophy, thus its’ increase in Ch-JFLS supports the decrease in COLX. Based on our culture models, JFLS undergo hypertrophy and may play a direct role in EBF in affected joints of patients through autocrine signaling.

The study was approved by Nemours Institution's Internal Review Board, approval numbers 82053 and 84709.

**Fig. 1 (abstract A23).**
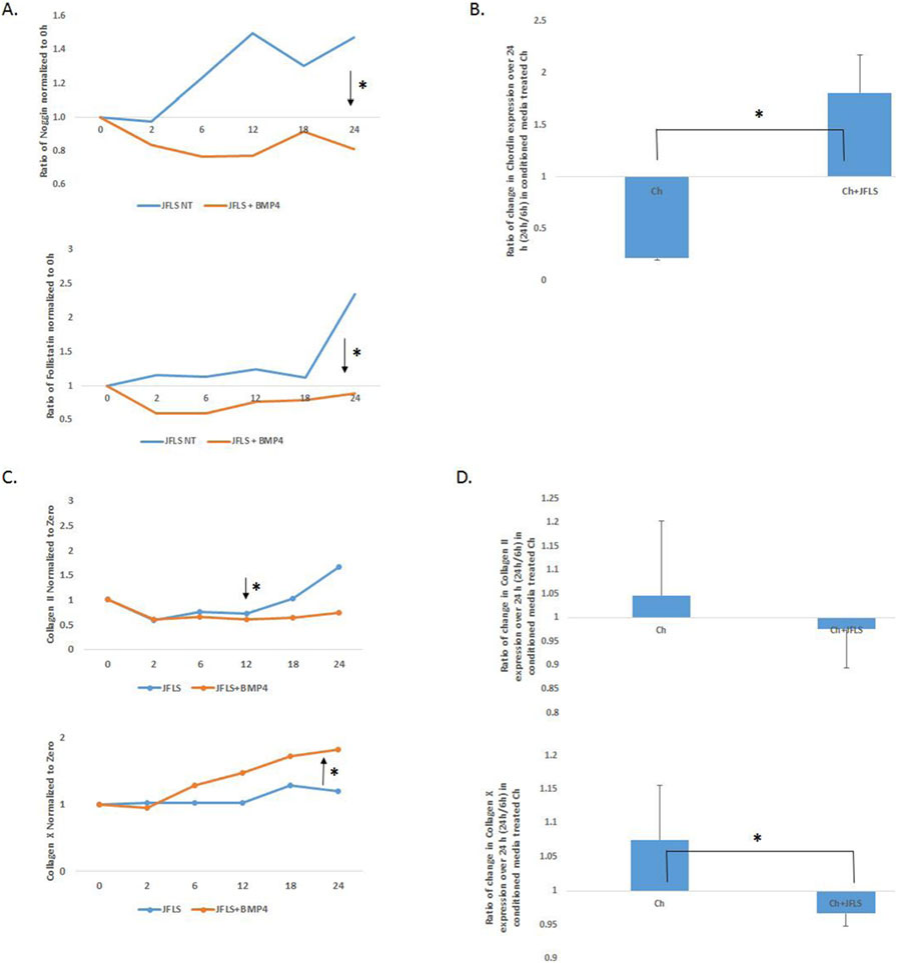
Response of fibroblast-like synoviocytes to BMP4 and response of chondrocytes to FLS-conditioned media. Exogenous BMP4 causes a significant decrease in NOG and FST expression in JFLS (A). Ch cultured in JFLS conditioned media have a significant increase in CHRD expression (B). COL2 decreases while COLX increases in BMP4 treated JFLS (C). COLX significantly decreases in Ch cultured in JFLS conditioned media (D)

## A24 Health-Related Quality of Life and Functioning in Children with JIA, JIA-Associated Uveitis, and Other Uveitis Types

### Joseph McDonald, Virginia Miraldi Utz, Theresa Hennard, Najima Mwase, Amy Cassedy, Sheila Angeles-Han

#### Cincinnati Children's Hospital Medical Center, Cincinnati, OH, USA

##### **Correspondence:** Joseph McDonald

Background : Pediatric uveitis can lead to ocular complications and blindness. The ophthalmic exam is used to assess outcomes. However, it likely underestimates the true impact of disease, since it does not account for the effect of vision impairment and the burden of chronic disease management on quality of life. Our goal is to examine health-related quality of life (HRQOL), mental health, physical disability, visual function, and vision-related QOL in children with JIA-associated uveitis (JIA-U).

Methods : We reviewed records of 62 children (22 JIA alone, 22 JIA-U, 18 other uveitis types). Parents and patients completed questionnaires on general QOL (Pediatric Quality of Life Inventory- PedsQL), depression and anxiety (Revised Children’s Anxiety and Depression Scale- RCADs), physical functioning (Childhood Health Assessment Questionnaire- CHAQ), and visual function and vision-related QOL (Effect of Youngsters’ Eyesight on QOL-EYE-Q). We used analysis of variance to compare disease groups: 1) children with JIA vs. JIA-U, and 2) children with JIA vs. other uveitis types.

Results : Of the 62 children most were non-Hispanic (98%), Caucasian (86%), and female (77%). Among uveitis patients, most had bilateral (73%), anterior disease (83%), with minimal disease activity (93% with inactive disease) and normal vision (100% visual acuity 20/40 or better) (Table 1). Children with JIA-U had worse EYE-Q vision-related QOL scores compared to those with JIA alone for Parent (1.72 vs. 1.93, p=0.03) and Child reports (1.76 vs. 1.96, p=0.05) (Table 2). There were no differences in EYE-Q total scores or EYE-Q visual function scores for Parent or Child reports. Children with other uveitis types had even worse EYE-Q vision-related QOL scores compared to those with JIA alone for Parent (1.62 vs. 1.93, p=0.01) and Child reports (1.73 vs. 1.96, p=0.03) (Table 2). Children with JIA, JIA-U, and other uveitis types had similar Parent and Child reports of PedsQL, RCADS, and CHAQ.

Conclusions : We describe HRQOL, mental health, physical function, visual function, and vision-related QOL of children with JIA-U. Children with uveitis had worse vision-related QOL compared to children with JIA alone. All children had similar visual function secondary to all groups having normal vision and mostly inactive uveitis. Our findings highlight the importance of including uveitis-specific disease measures in the assessment of the impact of uveitis. Uveitis is a complex chronic disease in childhood and even patients who have a normal eye exam and minimal disease activity can have disease specific burdens that may only be identified using an integrated approach.

This study received IRB approval #2016-8189 through continuing review on 10/29/18.

Acknowledgements: Dr. Angeles-Han support: CARRA-Arthritis Foundation Small Grant award. We thank the subjects for their participation.

**Table 1 (abstract A24).** Characteristics of Children with Juvenile Idiopathic Arthritis and Uveitis

N (%), unless indicated	Overall	JIA^a^ alone (n = 22)	JIA-U^b^ (n = 22)	Other U^c^ (n = 18)
Characteristics				
Demographics				
Age, Mean (SD)^d^	13.0 (3.3)	12.9 (2.8)	12.6 (3.4)	13.5 (3.9)
Gender, Male	14 (22.6)	2 (9.1)	4 (18.2)	8 (44.4)
Race				
White	53 (85.5)	21 (95.5)	20 (90.9)	12 (66.7)
African American	5 (8.1)	1 (4.5)	1 (4.5)	3 (16.7)
American Indian/Alaskan Native	0 (0.0)	0 (0.0)	0 (0.0)	0 (0.0)
Asian	1 (1.6)	0 (0.0)	1 (4.5)	0 (0.0)
Multi-racial	1 (1.6)	0 (0.0)	0 (0.0)	1 (5.6)
Unknown/Declined	2 (3.2)	0 (0.0)	0 (0.0)	2 (11.1)
Ethnicity, Hispanic	1 (1.6)	0 (0.0)	1 (4.7)	0 (0.0)
JIA Characteristics				
Age at Diagnosis, Mean (SD)	6.3 (4.6)	7.7 (4.3)	4.9 (4.2)	
Duration of Disease, years, Mean (SD)	6.5 (4.3)	5.2 (3.7)	7.7 (4.7)	
JIA Subtype				
Oligoarticular Persistent	14 (31.8)	10 (45.5)	4 (18.2)	
Oligoarticular Extended	9 (20.5)	3 (13.6)	6 (27.3)	
Polyarticular RF(-)	17 (38.6)	9 (40.9)	8 (36.4)	
Uveitis Characteristics				
Age at Diagnosis, Mean (SD)	8.4 (4.5)		6.1 (4.0)	11.4 (3.0)
Duration of Disease, years, Mean (SD)	4.8 (4.3)		6.4 (4.7)	2.8 (2.8)
Bilateral Disease			17 (77.2)	12 (66.7)
Anterior Chamber Cells >0.5+			1 (4.6)	2 (11.1)
Visual Acuity 20/50 or worse			0 (0.0)	0 (0.0)
Location				
Anterior			22 (100.0)	13 (72.2)
Intermediate			1 (4.5)	3 (1.7)
Posterior			0 (0.0)	1 (5.5)
Panuveitis			0 (0.0)	2 (11.1)
Ocular Complications, ever				
Glaucoma or Glaucoma Suspect	21 (33.9)		11 (50.0)	10 (55.6)
Cataracts	16 (25.8)		10 (45.5)	6 (33.3)
Synechiae	11 (17.7)		8 (36.4)	3 (16.7)
Band Keratopathy	4 (6.5)		1 (4.5)	3 (16.7)
Amblyopia	3 (4.8)		2 (9.1)	1 (5.6)
Cystoid Macular Edema	1 (1.6)		1 (4.5)	0 (0.0)
Other^e^	1 (1.6)		0 (0.0)	1 (5.6)
Treatment, at time of visit				
Steroid drops	24 (38.7)		11 (50.0)	13 (72.2)
Dilating drops	2 (3.2)		2 (9.1)	0 (0.0)
IOP-lowering drops^f^	5 (8.1)		1 (4.5)	4 (22.2)
Methotrexate oral	12 (19.4)	5 (22.7)	5 (22.7)	2 (11.1)
Methotrexate SQ	10 (16.1)	3 (13.6)	4 (18.2)	3 (16.7)
Mycophenolate	2 (3.2)	0 (0.0)	1 (4.5)	1 (5.6)
Etanercept	4 (6.5)	4 (18.2)	0 (0.0)	0 (0.0)
Infliximab	12 (19.4)	1 (4.5)	6 (27.3)	5 (27.8)
Adalimumab	9 (14.5)	2 (9.1)	6 (27.3)	1 (5.6)
Abatacept	1 (1.6)	0 (0.0)	1 (4.5)	0 (0.0)
Tocilizumab	7 (11.3)	3 (13.6)	4 (18.2)	0 (0.0)

^a^JIA: juvenile idiopathic arthritis; ^b^JIA-U: JIA-associated uveitis; ^c^Other U: all other uveitis not associated with JIA; ^d^ SD: standard deviation; ^e^Other complications include: vitreous hemorrhage, optic disc edema, aphakia, choroidal neovascular membranes, choriorential scare, retinal neovascularization, retinal detachment, keratic precipitates, peri retinal fibrosis, floaters, blindness, ocular hypertension; ^f^IOP: Intraocular pressure

**Table 2 (abstract A24).** Patient Reported Outcome Measures in Children with Juvenile Idiopathic Arthritis, Juvenile Idiopathic Arthritis Associated Uveitis, and Other Uveitis

Measure, Mean (SD)^a^	JIA	JIA-U	Other Uveitis	p-value 1^b^ (JIA vs. JIA-U)	p-value 2^c^ (JIA vs. Other Uveitis)
EYE-Q^d^ Parent Total	1.93 (0.11)	1.82 (0.26)	1.80 (0.16)	0.07	0.04*
Visual Function	1.93 (0.13)	1.86 (0.26)	1.88 (0.14)	0.30	0.46
Vision-related QOL	1.93 (0.09)	1.72 (0.31)	1.62 (0.27)	0.03*	< 0.01*
EYE-Q^d^ Child Total	1.85 (0.14)	1.78 (0.29)	1.74 (0.24)	0.27	0.12
Visual Function	1.83 (0.17)	1.78 (0.29)	1.75 (0.31)	0.53	0.31
Vision-related QOL	1.96 (0.08)	1.76 (0.35)	1.73 (0.25)	<0.05*	0.03*
RCADS^e^ Parent Total	36.7 (7.9)	35.2 (7.2)	33.6 (5.2)	0.26	0.83
RCADS^e^ Child Total	41.2 (5.6)	43.5 (7.0)	41.6 (6.3)	0.48	0.19
PedsQL^f^ Parent Total	89.4 (12.1)	83.3 (14.8)	84.2 (15.3)	0.18	0.28
PedsQL^f^ Child Total	85.8 (9.3)	83.9 (15.4)	86.3 (10.6)	0.61	0.89
CHAQ^g^ Parent Total	0.75 (1.48)	0.8 (1.77)	0.5 (0.73)	0.91	0.61
CHAQ^g^ Child Total	1.36 (2.48)	1.5 (2.74)	0.35 (0.61)	0.84	0.17

*p<0.05; ^a^ SD: Standard Deviation; ^b^p-value 1: comparison of JIA and JIA-U; ^c^p-value 2: comparison of JIA and other uveitis; ^d^EYE-Q: Effects of Youngsters’ Eyesight on Quality of Life, scores range from 0-2, lower scores indicate worse visual function and vision-related QOL; ^e^RCADS: Revised Children’s Anxiety and Depression Scale, scores ≥ 70 indicate clinically significant anxiety and depression; ^f^PedsQL: Pediatric Quality of Life Inventory, scores range from 0-100, higher scores indicate better QOL; ^g^CHAQ: Childhood Health Assessment Questionnaire, scores range from (0-3), higher scores indicate higher difficulty with activities of daily living

## A25 A Patient-Engaged Approach to Refining a Psychological Therapy for Individuals with Childhood-Onset Lupus

### Natoshia Cunningham^67^, Lauren Fussner^320^, Ashley Danguecan^229^, Christine Le^67^, Angela Chapson^147^, Emily Nguyen^147^, Janel Thompson^147^, Allie Thompson^147^, Miranda Moyer^147^, Susmita Kashikar-Zuck^67^, Hermine Brunner^193^, Andrea Knight^229^

#### ^67^Cincinnati Children's Hospital Medical Center, Cincinnati, OH, USA; ^320^Yale Child Study Center, New Haven, CT, USA; ^229^The Hospital for Sick Children, Toronto, ON, Canada; ^147^ Patient; ^193^PRCSG, Cincinnati Children’s Hospital Medical Center, Cincinnati, OH, USA

##### **Correspondence:** Natoshia Cunningham

Background : Childhood-onset Systemic Lupus Erythematosus (cSLE) is a chronic, multisystem, autoimmune disease associated with significant disease burden. Youth with cSLE commonly experience symptoms of fatigue, depression, and pain, which can complicate disease management. The PI and study team previously developed and pilot tested the first tailored cognitive behavioral therapy (CBT) for youth with cSLE, the Treatment and Education Approach for Childhood-Onset Lupus (TEACH), a 6-session in-person treatment targeting fatigue, depressive symptoms, and pain (Table 1). Significant reductions in fatigue and depressive symptoms were reported among those who received the treatment. However, qualitative interviews with participants revealed several barriers to in-person treatment (e.g., distance to medical center, patient burden), indicating a need to modify the intervention to increase access to care and better engage patients and families. Thus, the goal of the current project was to partner with patients and families as co-investigators to modify TEACH.

Methods : The researchers partnered with an advisory co-investigative team of 5 cSLE patients and caregivers (3 patients, 2 caregivers) to refine the intervention. Co-investigator feedback on the TEACH protocol was gathered using qualitative methods. Feedback on intervention format and content was obtained during a semi-structured group phone conference.

Results : Overall, a telehealth adaptation was viewed as favorable, with a preference for core content delivered in a live/video format and supplemental content delivered via web platforms (Table 2). Based on this feedback, TEACH will be refined to be delivered in a telehealth format for future trials.

Conclusions : The refined TEACH protocol has the potential to increase access to care for those unable to complete traditional in-person treatment.

This project is part of an IRB-Approved research study at Cincinnati Children’s Hospital.

All participants provided consent and reviewed the abstract.

**Table 1 (abstract A25).** TEACH Core Content

Session	Attendee(s)	Adolescent Content	Young Adult Content
1	Participant, caregiver	Psychoeducation, caregiver guidelines	Psychoeducation, caregiver guidelines
2	Participant	Activity pacing, deep breathing, sleep hygiene	Activity pacing, pleasant activities, *partner/caregiver communication*
3	Participant (and caregiver if <18 years)	Pleasant activities, muscle relaxation, *parent-child communication*	Medication adherence strategies for young adults, sleep hygiene, relaxation strategies
4	Participant (and caregiver if ≥18 years)	Mindfulness, identifying automatic thoughts	Mindfulness, identifying automatic thoughts
5	Participant	Challenging automatic thoughts, problem solving	Challenging automatic thoughts, problem solving
6	Participant, caregiver	Self-advocacy, maintenance	Self-advocacy, maintenance

*Note.* Caregiver can be parent/partner/optional for participants ≥18 years. Content originally delivered via 6 1-hour in-person sessions. The current study will use remote delivery, including live telehealth sessions with supplemental materials (e.g., handouts) available via the web. *Italics* content is optional

**Table 2 (abstract A25).** Patient & Caregiver Co-Investigator Advisory Team Feedback on TEACH Protocol

Overall Themes	Core Content	Supplemental Content	Additional Feedback
+Telehealth adaptation viewed as favorable +Core content via real time/live video delivery +Supplemental content via web-based delivery	+In-person sessions adapted to live video +Opt out for less relevant material +Address memory issues	+Well formatted, helpful +Use of web delivery preferred +Interactive forms (e.g., symptom tracking) preferred	+Interventionist access between sessions +Consider including a social support element

## A26 Patterns of Ambulatory Health Care Utilization among Transition-Age Youth with Systemic Lupus Erythematosus

### Joyce Chang^55^, Andrea Knight^229^, Erica Lawson^267^

#### ^55^Children's Hospital of Philadelphia, Philadelphia, PA, USA; ^229^The Hospital for Sick Children, Toronto, ON, Canada; ^267^University of California San Francisco, San Francisco, CA, USA

##### **Correspondence:** Joyce Chang

Background : The period when patients with child-onset systemic lupus erythematosus (SLE) transition from pediatric to adult health care systems during early adulthood may be particularly high risk, with data suggesting higher rates of hospital admissions and age-adjusted mortality compared to children or older adults with SLE. We leveraged U.S. private insurance claims to describe patterns of ambulatory subspecialty care utilization in transition-age youth with SLE.

Methods : Using Clinformatics DataMart® (OptumInsight, Eden Prairie, MN) de-identified US administrative claims (2000-2016), we identified youth ages 15-25 years with at least 3 ICD9 or ICD10-CM diagnosis codes for SLE, each at least 30 days apart. Youth were categorized by pattern of outpatient rheumatology and nephrology care into those that 1) transferred from a pediatric to adult provider within 12 months, 2) were lost to follow-up (> 12 months between last pediatric visit and first adult visit or end of enrollment), 3) remained in pediatric care throughout enrollment, or 4) received adult care only. For those that transferred or were lost to follow-up, the last pediatric visit was considered the index date, and ≥ 6 months of eligibility before and after were required for inclusion. Time between the first adult and last pediatric visit was assessed in youth with overlapping pediatric and adult care.

Results : A total of 2,386 youth with SLE met inclusion criteria, of which 135 successfully transferred from a pediatric to adult provider within 12 months, 49 were lost to follow-up, 226 remained in pediatric care until the end of enrollment, and 1,976 received only adult care throughout the eligibility period (Figure 1). Of those that did transfer to an adult rheumatology/nephrology provider, 77 (57%) had unilateral transfers, while 58 (43%) had periods of overlapping pediatric and adult visits over a median of 12 months (IQR 4 – 27). The mean age at transfer was 18.4 (SD 2.1) (Table 1).

Conclusions : Patterns of pediatric and adult lupus subspecialty care utilization vary considerably among privately insured transition-age youth with SLE. Prolonged periods of overlapping pediatric and adult visits were common, though timing of transfer may differ between rheumatology and nephrology providers. Next steps include assessing longitudinal health outcomes in this transition-age cohort, including acute care utilization and medication adherence, as well as identifying patient characteristics associated with worse outcomes.

An exemption was approved for this study by the Institutional Review Boards of the Children's Hospital of Philadelphia and the University of California San Francisco (18-25472).

Acknowledgements: This project was funded a CARRA-Arthritis Foundation grant. The authors wish to acknowledge CARRA, and the ongoing Arthritis Foundation financial support of CARRA.

**Fig. 1 (abstract A26).**
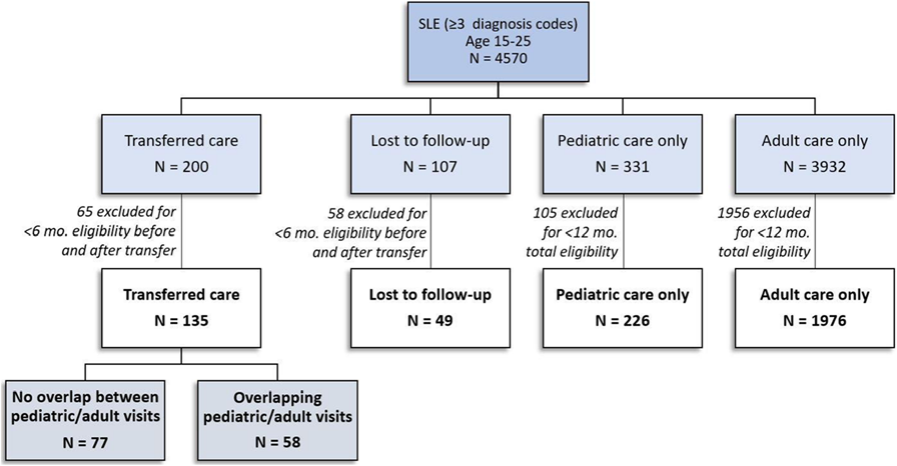
Ambulatory rheumatology/nephrology health care utilization patterns in transition-age youth with SLE. Categorization of health care utilization patterns based on outpatient rheumatology/nephrology visits. Transfer was defined as < 12 months between the last pediatric visit and the first adult visit during the eligibility period

**Table 1 (abstract A26).** Demographic characteristics by pattern of ambulatory lupus care in transition-age youth

	Transferred	Lost to follow-up	Pediatric only	Adult only
	N = 135	N = 49	N = 226	N = 1976
Age at last pediatric visit,	18.4	19.0	17.8	-
Mean (±SD)	(±2.1)	(±2.3)	(±2.6)	
Age at 1^st^ SLE code,	17.1	17.2	16.5	22.0
Mean (±SD)	(±4.2)	(±3.9)	(±2.7)	(±2.9)
Female, n (%)	116 (86%)	43 (88%)	181 (80%)	1798 (91%)

## A27 Partnering with Patients and Families to Coproduce Better Care and Outcomes

### Tzielan Lee^214^, Alysha Taxter^310^, Doreen Tabussi^115^, Jennifer Danielson^214^, Robert Shelton^310^, Cathy Kunchen^115^, Vincent Del Gaizo^41^, Lisa Johnson^78^, Jacob Casale^78^, Yukiko Kimura^137^, Suzanne Schrandt^19^

#### ^214^Stanford University School of Medicine, Stanford, CA, USA; ^310^Wake Forest University School of Medicine, Winston-Salem, NC, USA; ^115^Hackensack University Medical Center, Hackensack, NJ, USA; ^41^CARRA Inc., Milwaukee, WI, USA; ^78^Darmouth College, Hanover, NH, USA; ^137^ Joseph M. Sanzari Children's Hospital, Hackensack University Medical Center, Hackensack, NJ, USA; ^19^Arthritis Foundation, Atlanta, GA, USA

##### **Correspondence:** Tzielan Lee

Background : Coproduction of health care involves patients and families partnering with their physicians and care teams to engage in meaningful conversations to jointly assess how the patient is doing and to collaborate and decide next steps in the care and treatment plans. We believe these partnerships and conversations could be enhanced by utilizing an innovative information sharing platform, or dashboard tool, that incorporates patient-reported outcomes, key clinical data, and treatments/medications that are graphically displayed over time. This Arthritis Foundation-sponsored initiative is focused on building a Rheumatology Learning Health System, a framework that leverages data generated at point of care for improvement and research. This innovative dashboard tool will support coproduction of better health and outcomes for patients with JIA.

Methods : Multi-disciplinary clinical care teams and parent partners from Hackensack, Stanford and Wake Forest Universities met bi-weekly from April to December 2018 to engage in a human-centered co-design process. Teams mapped their current care processes, created end user personas, developed dashboard sketches, and participated in a nominal group technique to identify and cull data elements to populate an initial prototype of the dashboard. Teams contacted 36 parents to invite their participation in using a paper prototype dashboard during their child’s upcoming clinic visit to assess its usefulness in supporting coproduction of care.

Results : Eighteen team members (3 physicians, 9 care team members, 5 parents, and 1 patient) participated in 4 rounds of voting to narrow the initial set of coproduction dashboard data elements from 25 to 11 discreet elements (Table 1). Paper-based testing of the dashboard was conducted with 36 JIA patients (67% female and 33% male, ranging in age from 3 to 20 years) and 87% of parents and 80% of providers responded positively about its usefulness during discussions (Figure 1). It also uncovered important themes about the value of the dashboard, such as the ability to share questions and concerns in advance of the visit, enhance pre-visit planning, and visualize data over time to help make decisions.

Conclusions : Using a human-centered design process with 3 pediatric rheumatology pilot site teams yielded a blueprint for a dashboard tool and promising early results for its use at point of care to foster meaningful conversations and shared decision making about care and treatment plans between patients and families and their physicians and care teams. Creation of an electronic dashboard is planned for launch in November 2019.

This study received appropriate approval from the Dartmouth College Committee for the Protection of Human Subjects.

**Table 1 (abstract A27).**
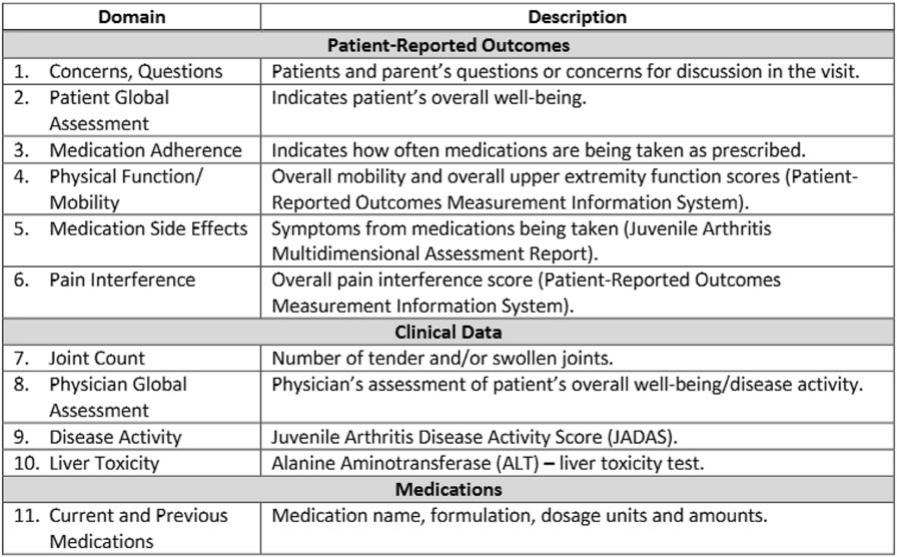
Dashboard Data Elements

**Fig. 1 (abstract A27).**
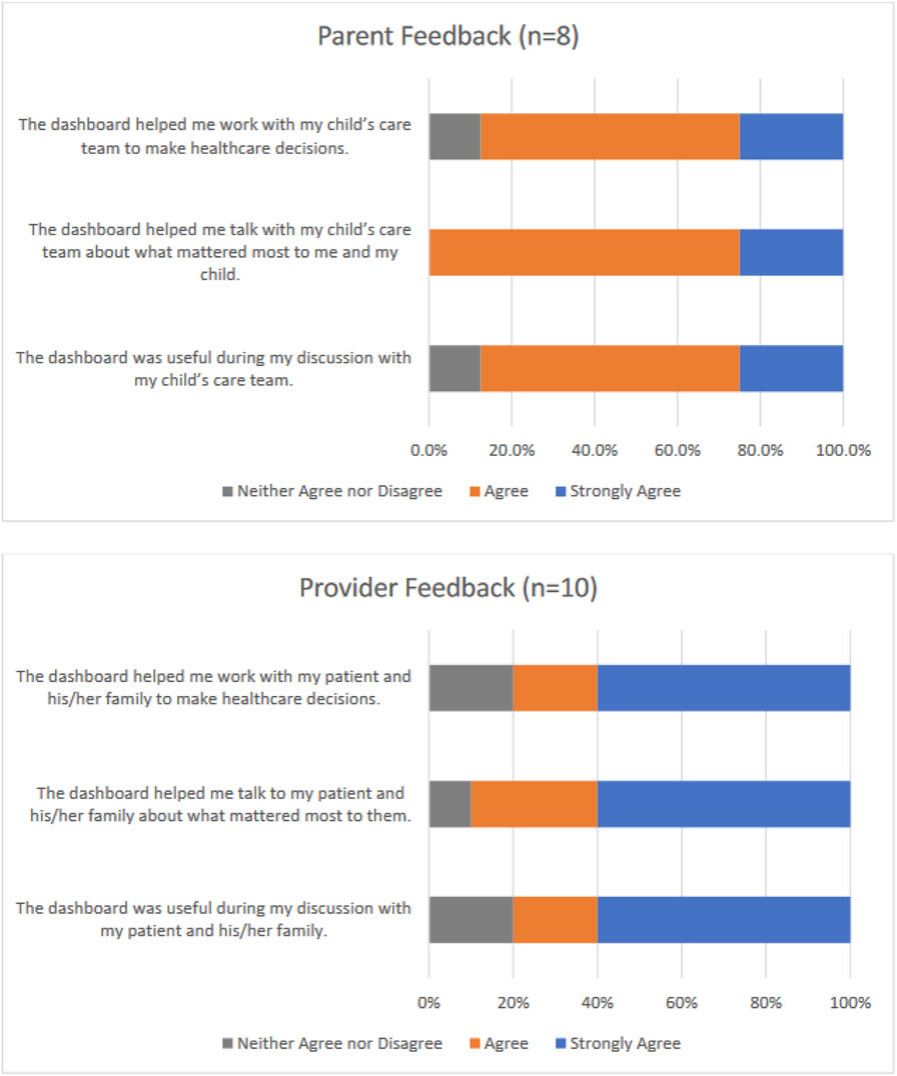
Parent and Provider Results from Dashboard Paper-Based Testing

## A28 Patients with Juvenile Idiopathic Arthritis in the Childhood Arthritis and Rheumatology Research Alliance (CARRA) Registry: Clinical Characteristics and Inception Cohorts

### Timothy Beukelman^258^, Yukiko Kimura^137^, Anne Dennos^95^, Kristin Siebenaler^95^, Marc Natter^116^, Kelly Mieszkalski^41^, Laura Schanberg^95^, for the CARRA Registry Investigators

#### ^258^University of Alabama at Birmingham, Birmingham, AL, USA; ^137^Joseph M. Sanzari Children's Hospital, Hackensack University Medical Center, Hackensack, NJ, USA; ^95^Duke University, Durham, NC, USA; ^116^Harvard University, Cambridge, MA, USA; ^41^CARRA Inc., Milwaukee, WI, USA

##### **Correspondence:** Timothy Beukelman

Background : In July 2015, the CARRA Registry was re-established as a multi-center observational study that collects essential data from persons with childhood-onset rheumatic diseases. The primary objective is to evaluate the safety of therapeutic agents. Key secondary objectives are to document the clinical courses and drug treatment patterns of patients and to evaluate clinical outcomes and their determinants, including treatment and other factors. This abstract describes the clinical characteristics of the patients with juvenile idiopathic arthritis (JIA) in the CARRA Registry at the time of enrollment and quantifies the number of patients in inception cohorts of new diagnosis and new medication use.

Methods : The CARRA Registry enrolled patients with JIA from CARRA pediatric rheumatology care centers throughout the United States and Canada. Initially, there was preferential enrollment of patients who were newly diagnosed, newly starting medications, had a polyarthritis course, or had systemic arthritis. In July 2017, enrollment was opened to all patients diagnosed with JIA. We present characteristics at enrollment for patients with JIA enrolled prior to 02 January 2019. To facilitate retrospective cohort studies within the Registry, we determined the number of patients who were newly diagnosed at enrollment or who newly started selected medications at or any time after enrollment. Missing values were ignored.

Results : As of 02 January 2019, there were 6,707 patients with JIA enrolled in the CARRA Registry from 63 different CARRA centers; 19 centers had enrolled at least 100 patients with JIA. The clinical characteristics of the patients at enrollment are shown in Table 1. With respect to inception cohorts, 1,854 (28%) patients were enrolled within 6 months of their diagnosis of JIA. The number of patients who newly started selected medications at or after enrollment is shown in Table 2.

Conclusions : The CARRA Registry has successfully enrolled over 6,700 patients with JIA in the 3 years since its inception. More than one-quarter of the patients were enrolled within 6 months of diagnosis. There are sufficient numbers of new medication initiations among patients enrolled in the Registry to begin to rigorously assess medication effectiveness and safety.

Acknowledgements: This work could not have been accomplished without the aid of the following organizations: The NIH’s National Institute of Arthritis and Musculoskeletal and Skin Diseases (NIAMS) & the Arthritis Foundation. We would also like to thank all participants and hospital sites that recruited patients for the CARRA Registry.

The study was approved by the Duke University Institutional Review Board.

**Table 1 (abstract A28).** See text for description

Characteristic	Frequency (%) or Median (25-75%)
Age at enrollment (years)	12.0 (7.0 – 15.0)
Female	4,760 (71%)
White Race	5,432 (81%)
Private health insurance	4,515 (67%)
Disease duration (years)	2.5 (0.4 – 6.1)
ILAR category:	
Oligoarthritis, persistent	1,634 (24%)
Oligoarthritis, extended	519 (8%)
Polyarthritis, RF-	2,184 (33%)
Polyarthritis, RF+	472 (7%)
Psoriatic arthritis	490 (7%)
Enthesitis related arthritis	687 (10%)
Systemic arthritis	571 (9%)
Undifferentiated arthritis	147 (2%)
ANA+	3,004 (45%)
RF+	556 (8%)
CCP+	447 (7%)
HLA-B27+	545 (8%)
Polyarthritis course	4,269 (64%)
Uveitis, ever	721 (11%)
Number of active joints	1 (0 – 3)
Physician global assessment	1 (0 – 3)
Active enthesitis	469 (7%)
Clinically active sacroiliitis	237 (4%)

Clinical characteristics at enrollment (N=6,707)

**Table 2 (abstract A28).** See text for description

Medication	Number of patients newly starting at or after enrollment
Methotrexate	898
Leflunomide	168
Sulfasalazine	95
Adalimumab	711
Etanercept	668
Infliximab	160
Abatacept	129
Tocilizumab	174
Anakinra	53
Canakinumab	81

Number of patients who newly started selected medications at or after enrollment (N=6,707)

## A29 Characteristics of Patients Enrolled in the FiRst Line Options for Systemic JIA Treatment (FROST) Consensus Treatment Plan Study

### Timothy Beukelman^258^, Peter Nigrovic^116^, George Tomlinson^291^, Vincent Del Gaizo^41^, Anne Dennos^95^, Mary Ellen Riordan^137^, Laura Schanberg^95^, Yukiko Kimura^137^, for the FROST CARRA Registry Investigators

#### ^258^University of Alabama at Birmingham, Birmingham, AL, USA; ^116^Harvard University, Cambridge, MA, USA; ^291^University of Toronto, Toronto, ON, Canada; ^41^CARRA Inc., Milwaukee, WI, USA; ^95^Duke University, Durham, NC, USA; ^137^Joseph M. Sanzari Children's Hospital, Hackensack University Medical Center, Hackensack, NJ, USA

##### **Correspondence:** Timothy Beukelman

Background : The optimal initial treatment for systemic juvenile idiopathic arthritis (sJIA) is unclear. Uncontrolled reports suggest that early treatment with biologic agents likely produces superior short-term clinical outcomes, but many patients may respond well to non-biologic therapies. To further study the initial treatment of sJIA, the Childhood Arthritis and Rheumatology Research Alliance (CARRA) developed Consensus Treatment Plans (CTPs) to formalize and standardize current treatment practices into 4 CTPs: initial systemic glucocorticoid (GC); initial methotrexate (MTX) +/- GC; initial IL-1 inhibition (IL-1i) +/- GC; and initial IL-6 inhibition (IL-6i) +/- GC. The FiRst Line Options for Systemic JIA Treatment (FROST) is an observational study designed to assess the effectiveness and safety of each of the 4 CTPs and to the compare the CTPs containing initial biologic therapy (IL-1i and IL-6i) to those that do not contain initial biologic therapy (GC and MTX).

Methods : Patients with recent onset sJIA who are initiating therapy are considered for enrollment in FROST. In order to be eligible for FROST, participants must have fever for ≥2 weeks, arthritis for ≥ 10 days, and at least 1 of the following: evanescent rash, generalized lymphadenopathy, hepatomegaly, splenomegaly, or serositis. Treatment assignment is at the discretion of the treating physician and family. All data are collected in the CARRA Registry. Patient reported outcomes (PRO) of presence of fever and rash and pain scores are collected at home using mobile devices every 2 days during the first 4 weeks of the study. The primary study outcome is clinical inactive disease (Wallace ACR provisional definition) and cessation of GC therapy at 9 months. We report the baseline characteristics and selected PRO results in this abstract.

Results : Enrollment in the FROST study began in November 2016. As of 20 December 2018, 40 patients have been enrolled at 22 sites, and their baseline characteristics are shown in the Table. Overall, 53% of participants completed every requested PRO during the first 2 weeks of the study, and 63% have completed ≥50% of the requested home PRO during the first 4 weeks. All participants reported resolution of fever within 2 weeks of study entry, although several patients experienced subsequent recurrence of fever. Mean pain scores were reduced from 1.8 on Day 2 to 1.3 on Day 12. Study follow-up continues to accrue. Seventeen patients (43%) have completed the 9-month study visit when the primary study outcome is assessed.

Conclusions : Enrollment in the FROST study continues. Participants enrolled thus far appear to be representative of the general population of patients with sJIA. Home PRO collection is feasible through the CARRA Registry. Additional time is needed to enroll and observe a sufficient number of patients to assess the comparative effectiveness of initial treatments for sJIA.

Acknowledgements: This work could not have been accomplished without the aid of the following organizations: The NIH’s National Institute of Arthritis and Musculoskeletal and Skin Diseases (NIAMS) & the Arthritis Foundation. We would also like to thank all participants and hospital sites that recruited patients for the CARRA Registry. . Funding for this project is provided to CARRA, Inc. in part by Genentech, a member of the Roche Group; Genentech the sponsor participated in the design of the study and the planned data analysis, but did not participate in writing of this report or decision to submit the report for publication.

The study was approved by the Duke University Institutional Review Board.

**Table 1 (abstract A29).** See text for description

Characteristic	Proportion or Summary Statistic
Age (mean (sd))	7.0 (4.5)
Female (%)	40%
White Race (%)	68%
Black Race (%)	11%
Other Race (%)	22%
Elapsed Days Since Onset of Symptoms (mean (sd))	40 (46)
Elapsed Days Since Diagnosis (mean (sd))	6 (8)
Physician Global Assessment (mean (sd))	5.9 (2.2)
Parent Global Assessment (mean (sd))	5.1 (3.3)
Number of Active Joints (mean (sd))	6.4 (8.0)
sJIA rash (%)	85%
Generalized lymphadenopathy (%)	25%
Hepatomegaly (%)	15%
Splenomegaly (%)	15%
Serositis (%)	13%
Morning Stiffness >15 minutes (%)	37%
ESR (median (IQR))	71 (44-100)
C-RP (median (IQR))	12.3 (6.2-53.3)
Ferritin (median (IQR))	700 (236-1266)
Hemoglobin (median (IQR))	10.1 (9.1-11.1)
WBC (median (IQR))	14.7 (9.8-20.2)
Platelets (median (IQR))	453 (335-564)
AST (median (IQR))	34 (23-46)
ALT (median (IQR))	33 (16-51)
CHAQ (mean (sd))	1.1 (0.9)
Pain VAS (mean (sd))	2.9 (2.7)

Characteristics of Participants in FROST at study enrollment (N=40)

## A30 Formal Depression Screening in Pediatric Rheumatology Patients with Systemic Lupus Erythematosus: A Quality Improvement Process Review

### Rebecca Furru, Evan Mulvihill, Alana Goldstein-Leever, Kyla Driest, Vidya Sivaraman

#### Nationwide Children's Hospital, Columbus, OH, USA

##### **Correspondence:** Rebecca Furru

Background : Childhood onset-systemic lupus erythematosus (c-SLE) is a chronic, multisystem autoimmune disease with high rates of morbidity, including neuropsychiatric effects. Youth with c-SLE face the burden of chronic disease and treatment demands, along with elevated risk for depression. Depression has been associated with poor disease control in c-SLE and impaired psychosocial and functional outcomes. Using quality improvement methodology, annual depression screening was implemented and sustained as a standard of clinical care within a pediatric rheumatology clinic setting, with the aims of increasing detection of depressive symptoms and linkage to appropriate mental health resources.

Methods : The primary goal was to increase the percentage of c-SLE patients screened from 0 to 80% in 1 year. (Figure 1). Patients were deemed eligible for screening based on c-SLE diagnosis, age >12 years, and need for annual screening. If due for screening, patients were provided with the Patient Health Questionnaire 9 (PHQ-9), and determined to be at “no/minimal risk,” “mild risk,” “moderate risk,” or “high/severe risk” for depression (Figure 2). Plan, Do, Study, Act cycles were used to determine feasibility and address identified barriers to routine screening.

Results : The project aim was met and has been sustained since July 2018 (Figure 3). Since project initiation, 71 of 86 eligible patients (82.5%) have completed annual screening. 52% (n=37) of screened patients endorsed no/minimal depressive symptoms, while 48% (n=34) endorsed depressive symptoms ranging from mild (n=16) to moderate (n=13), or high/severe (n=5) risk. Of 34 patients with depressive symptoms, 65% (n=22) were linked with mental health services.

Conclusions : Our results support the feasibility of annual depression screening within a rheumatology clinic setting and prevalence of depression among patients with c-SLE, with nearly half of patients endorsing mild to severe depression risk. 35% of patients with depression symptoms were without established treatment and provided with education, normalizing discussion, and mental health referrals as indicated, including those with moderate to severe symptoms; thus, supporting the importance of implementing screening as a standard of care. Future steps include expanding screening to include those with other forms of rheumatologic disease.3

No IRB review required for completion of Quality Improvement project

**Fig. 1 (abstract A30).**
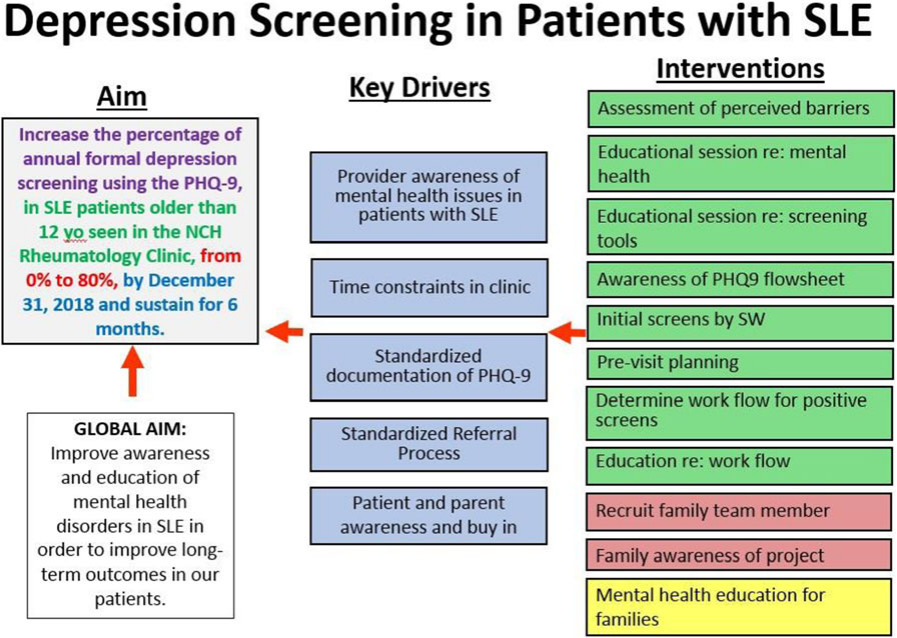
See text for description. Decision tree utilized to determine risk stratification based on PHQ-9 score and standardized intervention response

**Fig. 2 (abstract A30).**
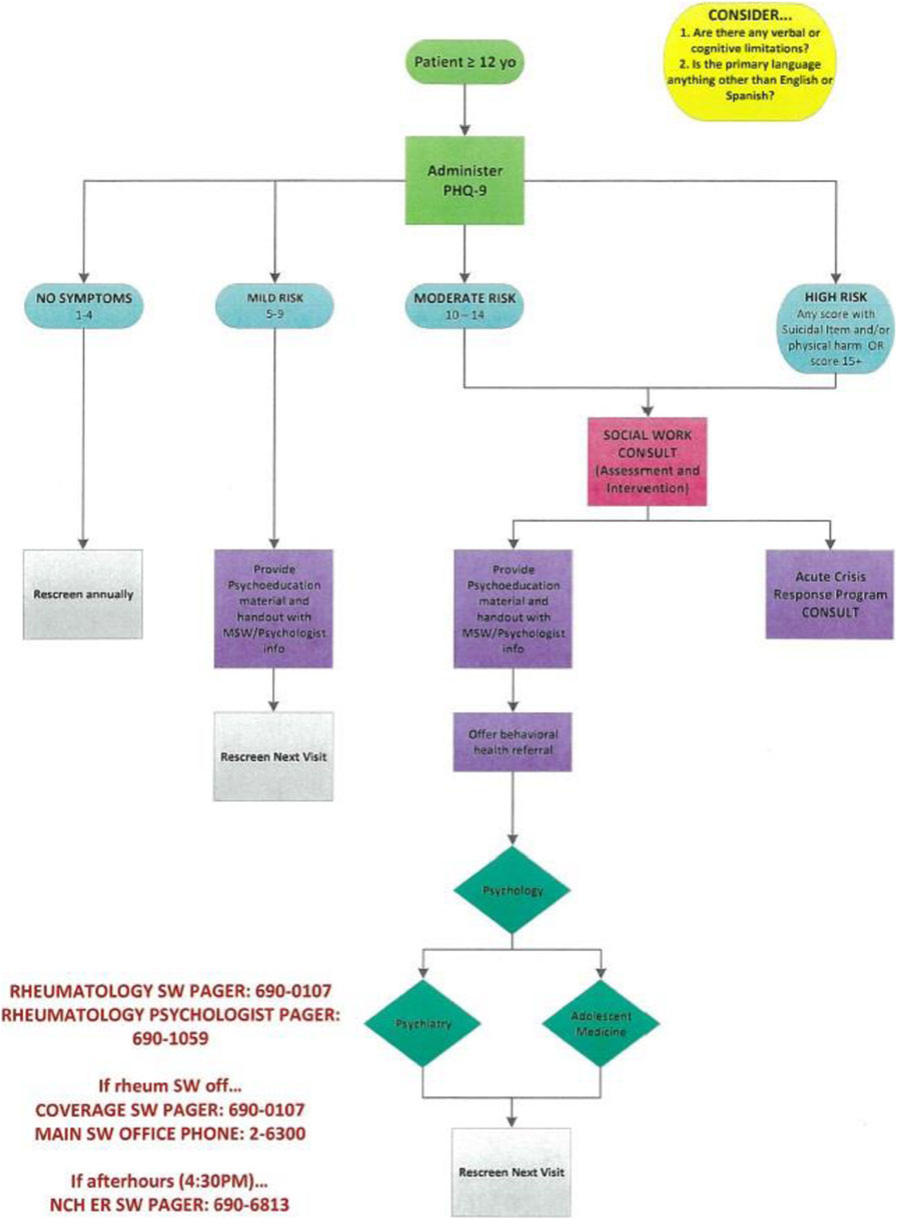
See text for description

**Fig. 3 (abstract A30).**
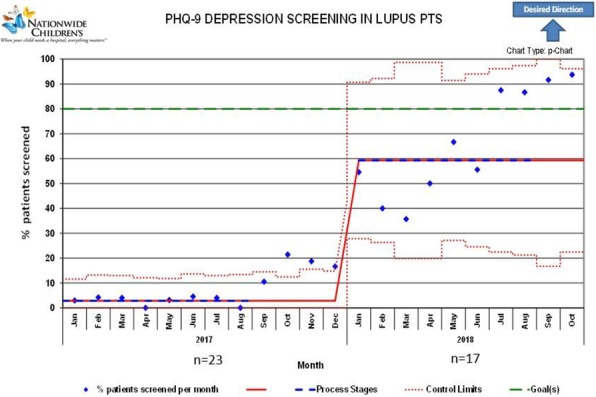
See text for description. Graph demonstrating number of completed PHQ-9 screenings per month since initiating QI process

## A31 Disability and Health-Related Quality of Life Outcomes in Patients With Systemic or Polyarticular Juvenile Idiopathic Arthritis Treated With Tocilizumab in Randomized Controlled Phase 3 Trials

### Hermine Brunner^193^, Chen Chen^193^, Alberto Martini^178^, Graciela Espada^178^, Rik Joos^178^, Jonathan Akikusa^178^, Jeffrey Chaitow^178^, Maria Luz Gámir Gámir^125^, Yukiko Kimura^137^, Christoph Rietschel^178^, Daniel Siri^178^, Elzbieta Smolewska^178^, Heinrike Schmeling^11^, Diane Brown^193^, Fabrizio De Benedetti^132^, Daniel Lovell^193^, Bin Huang^193^, Nicola Ruperto^178^

#### ^193^PRCSG, Cincinnati Children’s Hospital Medical Center, Cincinnati, OH, USA; ^178^Paediatric Rheumatology International Trial Organisation (PRINTO) Coordinating Centre, Genova, Italy; ^125^Hospital Ramon y Cajal Unidad de Reumatologia Pediatrica, Madrid, Spain; ^137^Joseph M. Sanzari Children's Hospital, Hackensack University Medical Center, Hackensack, NJ, USA; ^11^Alberta Children’s Hospital/University of Calgary, Calgary, AB, Canada; ^132^IRCCS Ospedale Pediatrico Bambino Gesù, Rome, Italy

##### **Correspondence:** Hermine Brunner

Background : Tocilizumab (TCZ) was approved for the treatment of systemic juvenile idiopathic arthritis (sJIA) and polyarticular juvenile idiopathic arthritis (pJIA) based on the results of 2 large phase 3 clinical trials. Physical function, measured by the Childhood Health Assessment Questionnaire–Disability Index (CHAQ-DI), and health-related quality of life (HRQOL), measured by the Child Health Questionnaire (CHQ), were evaluated in both trials. The objective of this post hoc analysis of data from both phase 3 trials of TCZ was to examine measures of disability and HRQOL in patients with sJIA or pJIA treated with TCZ for up to 2 years.

Methods : For sJIA patients, changes within 3 months of treatment initiation with TCZ (baseline) were compared between TCZ- and placebo (PBO)–treated patients using CHAQ-DI, pain-global assessment, physician (MD) global assessment, and patient global assessment using analysis of variance adjusted for treatment group. Changes in CHAQ-DI overall and domain scores from baseline to 2 years were compared for sJIA and pJIA patients treated with TCZ using the unpaired t test; similarly, for sJIA patients treated with TCZ, changes in CHQ domain and summary scores between baseline and 2 years were assessed.

Results : sJIA patients experienced clinically relevant improvement of physical function (CHAQ-DI) and reduction in pain (pain-global). sJIA patients treated with TCZ had significantly improved socialization, behavior, mental health, and CHQ-psychosocial summary scores after 3 months compared with those receiving PBO (Table 1). Marked improvement in all CHAQ-DI domains over 2 years was observed with TCZ treatment in both sJIA and pJIA patients (Table 2); improvement rates in patient well-being (patient-global) were 87.7% in sJIA patients and 83.4% in pJIA patients. There was also significant improvement (p < 0.05) in most domains of HRQOL (CHQ-domain scores) in patients with sJIA; of note, sJIA patients experienced marked improvement in mean scores from baseline to week 104 for pain/discomfort (31.7 to 75.3), self-esteem (61.0 to 76.0), mental health (62.1 to 76.8), and social limitation-emotional (52.6 to 86.2).

Conclusions : TCZ treatment resulted in statistically significant and clinically relevant improvements in function and HRQOL in patients with sJIA or pJIA over 2 years.

The protocols for the original trials were approved by the Institutional Review Boards and Ethics Committees of all participating institutions.

**Table 1 (abstract A31).** Change in HRQOL and Disability Within 3 Months of Starting Therapy in sJIA Patients*

Variable	Placebo n = 37		Tocilizumab n = 75		Difference	
	Baseline	Week 12	Baseline	Week 12	At Week 12	In Change From Baseline to Week 12^‡^
CHAQ-DI	1.66 (0.82)	1.17 (0.99)	1.74 (0.79)	0.94 (0.77)	–0.23	–0.31 (–0.62, 0.00)
Activity	1.86 (1.00)	1.30 (1.18)	1.93 (0.99)	1.19 (1.11)	–0.11	–0.18 (–0.61, 0.25)
Rising	1.51 (0.96)	1.00 (1.08)	1.55 (0.93)	0.79 (0.92)	–0.21	–0.25 (–0.67, 0.18)
Dressing and grooming	1.84 (1.09)	1.27 (1.19)	1.91 (1.02)	1.18 (1.00)	–0.09	–0.16 (–0.54, 0.22)
Eating	1.22 (1.18)	0.89 (1.02)	1.49 (1.06)	0.64 (0.76)	–0.25	–0.53 (–0.96, –0.09)
Grip	1.65 (1.03)	1.11 (1.10)	1.81 (0.91)	0.97 (1.00)	–0.13	–0.3 (–0.73, 0.13)
Hygiene	1.69 (1.04)	1.19 (1.17)	1.75 (1.00)	0.96 (1.08)	–0.23	–0.29 (–0.71, 0.14)
Reach	1.89 (1.05)	1.46 (1.14)	1.95 (0.96)	1.04 (0.94)	–0.42^†^	–0.47 (–0.86, –0.08)
Walking	1.62 (0.92)	1.11 (1.07)	1.52 (0.98)	0.76 (0.96)	–0.35	–0.25 (–0.62, 0.12)
VAS						
Pain-global	53.51 (22.35)	28.19 (27.17)	61.4 (23.98)	22.33 (24.21)	–5.86	–13.74 (–25.92, –1.56)
Physician-global	61.35 (21.12)	25.49 (24.01)	69.63 (15.65)	21.75 (19.20)	–3.74	–12.02 (–21.52, –2.51)
Patient-global	56.27 (21.2)	29.38 (25.93)	60.28 (23.78)	21.29 (21.88)	–8.09	–12.09 (–23.36, –0.83)
CHQ						
Global health	37.43 (28.06)	51.56 (21.98)	30.54 (25.66)	59.51 (26.57)	7.95	13.79 (0.34, 27.23)
Physical function	34.98 (25.56)	56.16 (34.18)	35.04 (27.78)	69.02 (29.56)	12.86	16.75 (4.16, 29.34)
Role/social limitation-physical	42.13 (30.21)	66.16 (35.47)	41.11 (33.37)	80.32 (26.59)	14.16^†^	16.2 (0.23, 32.18)
Role/social limitation-emotional	51.54 (34.24)	69.7 (34.5)	53.04 (34.20)	82.72 (25.49)	13.02	15.43 (–1.04, 31.91)
Pain/discomfort	34.72 (19.93)	54.85 (28.63)	30.27 (21.12)	65.56 (26.10)	10.71	15.94 (4.41, 27.46)
Behavior	64.00 (14.74)	61.36 (15.98)	63.72 (12.23)	69.12 (12.12)	7.76^†^	7.20 (1.75, 12.64)
Mental health	59.97 (15.65)	66.36 (17.91)	63.07 (19.12)	75.14 (17.58)	8.78^†^	5.64 (–1.62, 12.9)
Self-esteem	61.76 (20.91)	69.72 (23.7)	60.56 (20.92)	74.98 (18.81)	5.25	8.16 (–0.29, 16.61)
General health	41.76 (13.85)	45.08 (13.48)	42.20 (14.43)	39.96 (14.97)	–5.11	–4.59 (–10.19, 1.01)
Parental emotional	38.43 (20.44)	52.78 (26.24)	39.44 (25.24)	62.38 (25.64)	9.61	10.16 (–0.76, 21.07)
Parental time	57.72 (26.54)	69.02 (23.2)	60.59 (28.73)	72.99 (29.12)	3.97	2.08 (–10.83, 15)
Family activity	50.67 (22.26)	65.03 (23.87)	59.72 (23.92)	75.02 (25.51)	10	3.60 (–6.21, 13.41)
Family cohesion	75.97 (19.92)	69.24 (26.70)	70.33 (23.81)	75.56 (22.21)	6.31	10.66 (2.38, 18.94)
CHQ-PhS	19.47 (12.85)	31.85 (17.02)	18.68 (13.84)	36.97 (13.99)	5.12	7.31 (1.13, 13.49)
CHQ-PsS	40.64 (8.61)	43.57 (11.33)	41.38 (11.45)	48.44 (9.29)	4.87^†^	4.85 (0.42, 9.27)

*Data are mean (SD) unless otherwise specified. †ANOVA, p < 0.05. ‡Median (min, max). ANOVA, analysis of variance; CHAQ-DI, Childhood Health Assessment Questionnaire–Disability Index; CHQ, Child Health Questionnaire; HRQOL, health-related quality of life; PhS, physical summary; PsS, psychosocial summary; sJIA, systemic juvenile idiopathic arthritis; VAS visual analog scale

**Table 2 (abstract A31).** Change in HRQOL and Disability From Baseline to Week 104 in sJIA and pJIA Patients*

Variable	pJIA			sJIA		
	Baseline n = 188	Week 104 n = 155	*p*^†^	Baseline n = 112	Week 104 n = 95	*p*^†^
CHAQ-DI	1.39 (0.74)	0.28 (0.45)	<0.001	1.71 (0.8)	0.58 (0.72)	<0.001
Activity	1.56 (0.97)	0.29 (0.58)	<0.001	1.91 (0.99)	0.80 (1.06)	<0.001
Rising	1.32 (0.88)	0.19 (0.48)	<0.001	1.54 (0.94)	0.39 (0.73)	<0.001
Dressing and grooming	1.60 (1.01)	0.30 (0.66)	<0.001	1.88 (1.04)	0.73 (1.07)	<0.001
Eating	1.16 (1.01)	0.25 (0.57)	<0.001	1.40 (1.10)	0.38 (0.70)	<0.001
Grip	1.53 (0.94)	0.43 (0.76)	<0.001	1.76 (0.95)	0.56 (0.87)	<0.001
Hygiene	1.30 (1.03)	0.26 (0.60)	<0.001	1.73 (1.01)	0.57 (0.88)	<0.001
Reach	1.57 (0.90)	0.35 (0.60)	<0.001	1.93 (0.98)	0.76 (1.01)	<0.001
Walking	1.11 (0.89)	0.15 (0.46)	<0.001	1.55 (0.96)	0.43 (0.78)	<0.001
Parent global pain assessment	52.32 (26.94)	9.79 (18.21)	<0.001	58.79 (23.65)	7.28 (11.76)	<0.001
Physician global assessment	61.36 (20.74)	5.88 (11.45)	<0.001	66.89 (17.98)	7.40 (10.36)	<0.001
Patient global assessment	52.91 (25.04)	8.77 (16.73)	<0.001	58.96 (22.94)	7.25 (10.86)	<0.001

*Data are mean (SD). †Paired t test. CHAQ-DI, Childhood Health Assessment Questionnaire–Disability Index; HRQOL, health-related quality of life; pJIA, polyarticular juvenile idiopathic arthritis; sJIA, systemic juvenile idiopathic arthritis

## A32 Establishing the juvenile arthritis in Minnesota (JaMINN) study: collecting environmental and genetic data

### Colleen Correll, Michelle Roesler, Sara Kramer, Colleen Carter, Logan Spector

#### University of Minnesota, Minneapolis, MN, USA

##### **Correspondence:** Colleen Correll

Background : We have established the JaMINN Study (Juvenile Arthritis in Minnesota), a statewide population-based cohort of patients with JIA who live in Minnesota to identify genetic and environmental factors associated with disease onset and progression. Enrollment began in April 2018. We are collecting environmental exposure data through questionnaires and Minnesota birth records to identify exposures associated with JIA disease onset and flare. We are also collecting and storing DNA from patients and their parents for future analysis. Here we present preliminary data from the study.

Methods : Patients treated at the University of Minnesota (UMN) who are eligible for the study are approached for enrollment at the time of their clinic visit. For patients, blood is collected with their routine lab draw and is biobanked for future genetic testing. After patient enrollment, an oral DNA collection kit is mailed to the parents or given to them at the next follow-up visit. We are using the Dillman Total Design Method to identify, contact and enroll patients treated outside of UMN. For these participants, DNA is collected from patients and parents via mailed oral DNA collection kits. The environmental risk questionnaires are sent electronically by email and birth records are obtained from the Minnesota Department of Health.

Results : To date, we have 237 participants enrolled of the estimated 800 patients with JIA living in Minnesota (30%). Basic demographics are seen in Table 1. Number of genome trios and dyads collected to date can be seen in Table 2. Environmental risk questionnaire and birth record data will be available at the meeting in April.

Conclusions : At the current enrollment rate, we expect to enroll >90% of patients with JIA to this study which will be close to a perfectly population-based study. Environmental exposure risks based upon self-report questionnaires and birth records will be analyzed. We plan to conduct future genetic susceptibility and gene-environment interaction studies.

This study was approved by the UMN institutional review board (IRB # 0001169).

**Table 1 (abstract A32).** Characteristics of Participants enrolled in JaMINN Study

Female N (%)	174 (73)
Age, mean years (SD)	10.61 (3.97)
JIA classification by ILAR criteria N (%)	
Oligoarticular (extended or persistent)	92 (39)
Rheumatoid factor (RF)-negative polyarticular	69 (29)
RF-positive polyarticular	12 (5)
Enthesitis-related	24 (10)
Psoriatic	11 (5)
Systemic	21 (9)
Undifferentiated	7 (3)
Missing	1 (0.4)
Total	237 (100)

**Table 2 (abstract A32).** Number of genome trios or dyads available for analysis

Genome trios/dyads	
Complete case-parent trio	31
Mother-sibling-case trio	0
Father-sibling-case trio	0
Mother-case dyad	2
Father-case dyad	0
Total	33

## A33 Role of Ultrasound-guided Intra-articular Corticosteroid Injections in Juvenile Idiopathic Arthritis (JIA): A Single Center Retrospective Study

### Taha Moussa^272^, Deirdre De Ranieri^169^

#### ^272^University of Chicago Medicine, Chicago, IL, USA; ^169^Northwestern University Feinberg School of Medicine, Chicago, IL, USA

##### **Correspondence:** Taha Moussa

Background : The use of ultrasound (US) guided intra-articular corticosteroid injections (IACIs) is a common therapeutic option in the management of different types of JIA; however there are no standardized guidelines in pediatrics regarding their use in the treatment of arthritis. Optimal dosing and type of corticosteroid have not been established. Indications for use have not been adequately assessed, and providers vary in their use of IACI in their practice, from not using IACI at all to using IACI as mono-therapy. We hope to investigate how IACI are incorporated into patient care. IACI can be used as mono therapy in the treatment of arthritis in JIA, or in conjunction with DMARDS and/or NSAIDs for intermittent flares without changing systemic medications, or finally, to expeditiously treat synovitis while transitioning systemic therapy. Our retrospective chart review will be useful tool to understand and identify how IACI are being used to treat JIA in a busy urban academic setting. With this information, we will be able to evaluate not only which patients received IACI but also the outcomes associated with each case. Evaluating the use of IACI in clinical practice is important as there are currently no guidelines in place regarding optimal usage of this procedure. We hypothesize that outcomes will vary according to clinical practice.

Methods : Single center retrospective analysis of JIA patients who had US-guided IACI’s over the last 5 years (January 2013 to January 2018). This study includes children aged 0-16 who have been diagnosed with Juvenile Idiopathic Arthritis and received US-guided IACI’s. The evaluation of remission will be based on physician documentation in their clinic notes. Medication changes, if made after IACI will be noted. Other data will include which joints were injected, which medication was used and at what dose, and for repeat injections, the same information will be gathered.

Results : 90 children with different subtypes of JIA had at least one episode of US guided IACI between January 2013 to Jan 2018. IACI were used in different ways: as monotherapy (with or without NSAIDs), in conjunction with immunomodulatory therapy (IMT) without a change in IMT, or as a bridging therapy while transitioning between different IMTs. Outcome measures include remission as defined by physician documentation, physical disabilities such as leg length discrepancies and flexion contractures, as well as adverse medication side effects (experienced with IACI or IMT). In our evaluation, the outcome was different based on the JIA subtype and the indication of use. Better outcome was noticed when IACS injection was used as bridging therapy during transition of systemic IMT.

Conclusions : IACI is a valuable tool whose use has not been standardized in the treatment of JIA, resulting in significant variability in treatment across providers. In our experience, the optimal use of IACI is in conjunction with IMT (and often while transitioning between IMTs) in order to reduce the risk for long term complication of arthritis. Further prospective studies are needed to establish guidelines for the use of IACI in JIA.

This study was approved by the University of Chicago Medicine institutional review board (IRB18-0146).

## A34 HLA Findings in Youth with Pediatric Acute-onset Neuropsychiatric Syndrome (PANS)

### Avis Chan^213^, Jennifer Frankovich^213^, Jill Hollenbach^267^, Gonzalo Montero-Martin^213^, Margo Thienemann^213^, Bahare Farhadian^213^, Theresa Willett^213^, David Lewis^213^, Elizabeth Mellins^213^, Tanya Murphy^289^, Marcelo Fernandez-Vina^213^

#### ^213^Stanford University, Stanford, CA, USA; ^267^University of California San Francisco, San Francisco, CA, USA; ^289^University of South Florida, Tampa, FL, USA

##### **Correspondence:** Avis Chan

Background : Studies over the past decade have shown great overlap between mental health impairments and rheumatologic disorders. Pediatric Acute-onset Neuropsychiatric Syndrome (PANS) is characterized by abrupt (overnight) onset of obsessive compulsive disorder (OCD) and/or food restriction with two other specified neuropsychiatric symptoms. PANS patients have also been observed to have co-existing arthritis and autoimmune diseases, and/or first degree family members with autoimmune diseases.

Methods : High resolution HLA typing was performed on 74 consecutive Caucasian patients from the Stanford PANS Clinic who met PANS criteria, and 39 Caucasian patients with PANS/ PANDAS from the University of South Florida. We compared the samples to NIH INDIGO Healthy Control Cohort which consists of 1,000 samples collected from healthy European Americans (European ancestry, Caucasian ethnicity) across the US (n=1000). We applied two different next-generation sequencing (NGS) methodologies (in order to increase the throughput, accuracy, and resolution of genetic analysis by several orders of magnitude compared to the gold-standard Sanger sequencing). The healthy control cohort had HLA NGS sequencing as part of another study led by co-investigators of this study (Fernandez-Vina and Hollenbach). All patients and controls were typed for HLA-A, -B, -C, -DRB1, -DRB3/4/5, -DQA1, -DQB1, -DPA1 and -DPB1 loci.

Results : Significant HLA-B associations include: HLA-B38 (OR 2.05, 95% CI 0.95-4, p=0.030), HLA-B52 (OR 4.02, 95% CI 1.7-8.2, p=0.002), and HLA B27 (OR 2.01, 95% CI 1.1-3.6, p=0.008). Further investigation into HLA-B27 reveals that the B27:02 allele is strongly associated (OR 5.8, 95% CI 1.8-14.7, p=0.004). As these associated alleles share the HLA-Bw4 epitope, amino acid level analysis was performed and reveals significant amino acid level associations at positions 80-83 (the Bw4 epitope) (Table 1). There is no association found with the HLA Class II genes.

Conclusions : HLA associations that are reproducible and robust argue for immune system involvement in disease. We aim to analyze a second PANS cohort to determine if the above findings are reproducible and robust. If confirmed, this finding will support and add further evidence suggesting that PANS is an inflammatory disorder.

The study was approved by the Stanford University’s Internal Review Board, protocol 26922. This institution is in compliance with requirements for protection of human subjects, including 45 CFR 46, 21 CFR 50 and 56, and 38 CFR 16.

**Table 1 (abstract A34).** Amino acid level analysis revealed the association of HLA-BW4 epitope (defined by positions 80-83) in a 113 Caucasian patients with PANS compared to the NIH INDIGO Caucasian Donor Cohort

Position	Residue	OR (CI)	p-value
Position.80	I	2 (1.42-2.80)	<0.001
Position.81	L	0.65 (0.48-0.89)	0.004
Position.81	A	1.54 (1.13-2.08)	0.004
Position.82	R	0.62 (0.46-0.84)	0.001
Position.82	L	1.62 (1.19-2.19)	0.001
Position.83	G	0.62 (0.46-0.84)	0.001
Position.83	R	1.62 (1.19-2.19)	0.001

## A35 Dermal Histopathological Correlation with Genetic Signatures of Pediatric Localized Scleroderma

### Christina Schutt, Emily Mirizio, Claudia Salgado, Miguel Reyes-Mugica, Kaila Schollaert-Fitch, Kathryn Torok

#### University of Pittsburgh Medical Center, Pittsburgh, PA

##### **Correspondence:** Christina Schutt

Background : Localized scleroderma (LS) is a progressive disease of the skin and underlying tissue that causes significant functional disability and disfigurement, especially in developing children. Histopathology of LS skin biopsies typically displays a stronger inflammatory or fibrotic pattern depending on disease stage. To determine the transcriptome within inflammatory vs. fibrotic tissue, and to identify potential molecular targets, RNA sequencing (RNAseq) was compared to histology scoring of inflammatory infiltrate vs collagen deposition/ fibrosis. Differentially expressed genes (DEGs) in LS patients were compared to skin histopathological features to determine correlation.

Methods : RNAseq was performed on paraffin-embedded skin (n=15 LS, n=2 pediatric healthy) using the Illumina HTS using TrueSeq Access library preparation. Paired-end RNAseq data was aligned using CLC Genomics software and analyzed for DEGs. Genes were analyzed using DEG cutoffs of log fold change > ±2.0, adjusted p<0.05, and a false discovery rate (FDR) cutoff of <0.05 for Ingenuity Pathway Analysis (IPA). Statistical analysis was performed using Spearman’s correlation tests in GraphPad Prism. Skin biopsies were reviewed by 2 pathologists who determined 3 areas of inflammatory infiltrate per skin layer (papillary dermis, upper and lower reticular dermis) and counted total inflammatory cells (lymphocytes and plasma cells) per infiltrate, which determined a categorical inflammation score.

Results : CLC Genomics was used to determine significant DEGs between inflammation scores. IPA of DEGs revealed 44 highly significant canonical pathways (p<0.05). Humoral immune response, inflammatory response, and immune cell trafficking were among the most significant pathways. Within these there is distinct gene expression involving B lymphocyte (p<0.001, z= 1.23), mononuclear leukocyte (p<0.001, z= 1.712), and neutrophil (p<0.01, z= 0.926) activation. Genes of interest were correlated (Spearman’s r) to total inflammatory cell count for each sample. Significant moderate correlations (rs> ±0.5, p<0.01) with the number of inflammatory cells were found with: CXCL9, CXCL10, CXCL11, AGER, ELANE, IGHG1, IGHG3, IGHM, KLRC4, and TNC (Table 1).

Conclusions : The identified pathways and genes corresponding with the inflammatory infiltrate of LS samples indicate a unique genetic signature that is present during active disease with moderate-severe lymphoplasmacytic infiltrate. Further investigation into the relationship and functions of these genes will aid in advancing treatment options for LS patients. Additional study is underway investigating degree of fibrosis and gene correlates and their interaction with inflammatory signature.

Samples and clinical data were collected in accordance with the Declaration of Helsinki under University of Pittsburgh IRB# PRO11060222, PRO12070091, and PRO14080297, the National Registry for Childhood Onset Scleroderma. Informed consent was obtained from patients prior to participation.

**Table 1 (abstract A35).** See text for description

Gene	Spearman’s rho r_s_	P-value (2-tailed)
*CXCL9*	0.62	0.0085
*CXCL10*	0.68	0.0026
*CXCL11*	0.83	<0.0001
*IGHG1*	0.74	0.0007
*IGHG3*	0.75	0.0005
*IGHM*	0.65	0.0046
*AGER*	0.61	0.0095
*KLRC4-KLRK1*	0.76	0.0004
*TNC*	0.69	0.0021
*ELANE*	-0.65	0.0049

Correlation Analysis Between Total Inflammatory Cells vs Transcripts Per Kilobase Million (TPM) values of DEGs

## A36 Effector T cells: An Assessment of Activity in Polyarticular Juvenile Idiopathic Arthritis

### Anna Patrick^307^, Brent Graham^307^, Susan Thompson^64^, Thomas Aune^307^

#### ^307^Vanderbilt University Medical Center, Nashville, TN, USA; ^64^Cincinnati Children’s Hospital Medical Center, Cincinnati, OH, USA

##### **Correspondence:** Anna Patrick

Background : T cells play a critical role in the body’s response to pathogens. Naïve T helper (Th0) cells proliferate in response to antigen encounter but are not differentiated. Specific cytokines signal naive Th cells to express transcription factors causing differentiation to Th cell lineages, including Th1, Th2, and Th17 lineages. Re-activation causes a differentiated effector Th cell to secrete characteristic cytokines, which are IFNγ, IL-5 and IL-13, and IL-17 for Th1, Th2, and Th17 cells respectively. In juvenile idiopathic arthritis (JIA), specific Th cell lineage differentiation and cytokine secretion have not been studied. An ex vivo assay was developed using human peripheral blood mononuclear cells (PBMCs) wherein PBMC differentiate, re-activate, and secrete cytokines. In this study, we aimed to test effector Th cell differentiation and cytokine secretion in PBMCs from prepubescent children with JIA and healthy age-matched controls.

Methods : PBMCs collected at Vanderbilt Children’s Hospital or at Cincinnati Children’s Hospital are utilized in these studies. Following cytokine stimulation, PBMCs undergo ex vivo differentiation to Th0, Th1, Th2, and Th17 cells. After restimulation, effector cytokine secretion is measured by enzyme-linked immunosorbent assay. Intracellular protein expression is assessed by Western blot analysis. Additional experiments are performed based on the quantity of available PBMCs. 1) PBMCs are stimulated and proliferation measured with a fluorescent dye and flow cytometry. 2) Expression of specific cell surface markers is measured by flow cytometry. 3) Assessment of PBMCs for naïve or memory T cell phenotypes is quantified by the cell surface markers CD3, CD4, CD8, CD197, and CD45RA and flow cytometry. 4) All remaining PBMCs are subjected to ex vivo differentiation to Th0, Th1, and Th2 cells and collected for epigenetic analysis.

Results : PBMCs from 20 JIA and 20 age-matched control children were collected: 8 JIA patients and 20 healthy controls from Cincinnati, 12 JIA patients from Vanderbilt. All children were between 2 and 8 years old (average 62 months) at the time of sample donation. JIA patients are polyarticular (n=18) or extended oligoarticular (n=2), female (n=16), and ANA positive (n=19). Samples were collected at diagnosis (n=5). Preliminary studies comparing 7 JIA and 2 healthy controls found that differentiated effector Th cell cultures from children with JIA secrete much higher levels of characteristic cytokines than healthy control children. Surprisingly, undifferentiated Th0 cells from children with JIA secrete IFNγ.

Conclusions : Differentiated Th cell cultures from children with JIA express more IFNγ, IL-5 and IL-13, and IL-17 than cultures from healthy children. Mechanisms underlying differences between Th cell cultures from young children and children with JIA can reveal differences in the differentiation of naïve T cells into effector cells. The data gained from these studies may provide insight into JIA disease mechanisms that can be used to guide development of diagnostics and therapies.

The study was approved by the Vanderbilt University and Cincinnati Children’s Hospital Institutional Review Boards.

Acknowledgments: This project is supported by a CARRA – Arthritis Foundation Fellow Grant. The authors wish to acknowledge the ongoing Arthritis Foundation financial support of CARRA.

## A37 Improvement of Teratogenic Medication Risk Counseling in Adolescent Females in a Rheumatology Clinic Setting

### Kelly Wise, Fatima Barbar-Smiley, Veronica Mruk, Edward Oberle, Kyla Driest, Stephanie Lemle, Vidya Sivaraman, Cagri Yildrim Toruner, Stacy Ardoin, Elise Berlan

#### Nationwide Children's Hospital / The Ohio State University, Columbus, OH, USA

##### **Correspondence:** Kelly Wise

Background : The U.S. Food and Drug Administration has a voluntary Risk Evaluation and Mitigation Strategy (REMS) program for mycophenolate due to risk of first-trimester miscarriage and congenital malformations if the medication is used during pregnancy. The REMS program encourages patient education on teratogenic risk and pregnancy prevention. To signify completed education, a Patient-Prescriber Acknowledgement form is signed. Nationwide Children’s Rheumatology Division developed a similar consent form for teratogenic medications, such as methotrexate and leflunomide, which lack REMS programs. In an effort to improve teratogenic risk counseling, we created a quality improvement project with the aim to increase the percentage of female adolescents ≥14 years of age prescribed teratogenic medications that have signed teratogenic risk consent forms from 0% to 90% by June 2019, and sustain for 6 months.

Methods : Major interventions of the quality improvement project included the creation of methotrexate and leflunomide consent forms, pharmacist-facilitated education on teratogenic risk and pregnancy prevention with patients and providers, development of a pre-visit planning process, and ongoing education at staff meetings (Figure 1). We developed a workflow for point-of-care pregnancy testing for female patients prior to starting teratogenic medications and ongoing screening for females at high-risk for pregnancy.

Results : By November 2018, the percentage of female adolescents ≥14 years of age prescribed teratogenic medications that have signed teratogenic consent forms increased from 0% to 43% (Figure 2). One patient became pregnant while on methotrexate therapy during this project.

Conclusions : By implementing a streamlined process, we were able to improve teratogenic risk counseling for medications commonly used to treat rheumatic conditions in adolescent females. We are developing contraceptive plans, including referrals to pediatric and adult gynecologists for female patients at high-risk of pregnancy. We plan to assess the usage of contraceptive methods and unintended pregnancies in this patient population in order to determine the overall impact of teratogenic counseling in preventing pregnancy.

This project is deemed quality improvement and is exempt from review by the Institutional Review Board.

**Fig. 1 (abstract A37).**
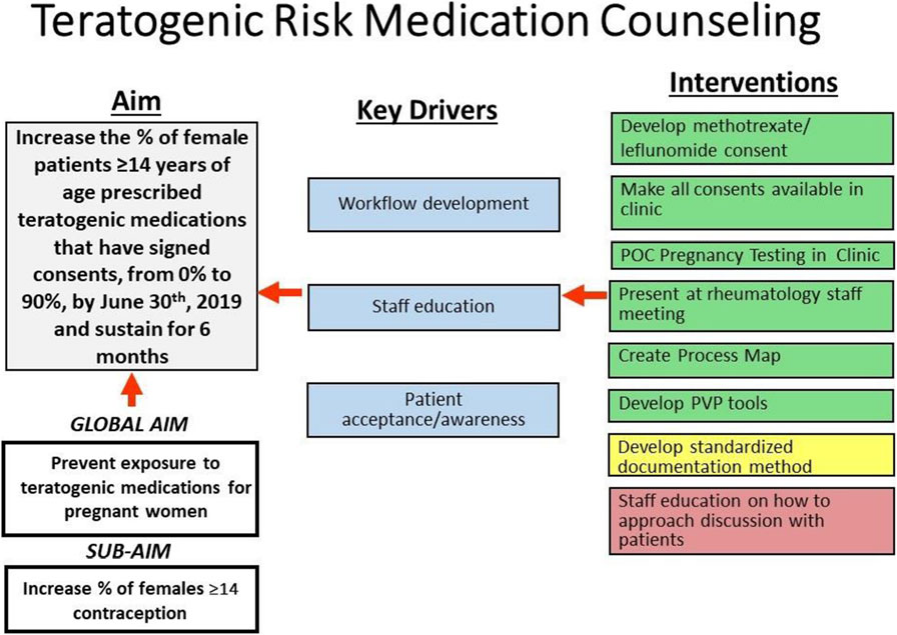
See text for description

**Fig. 2 (abstract A37).**
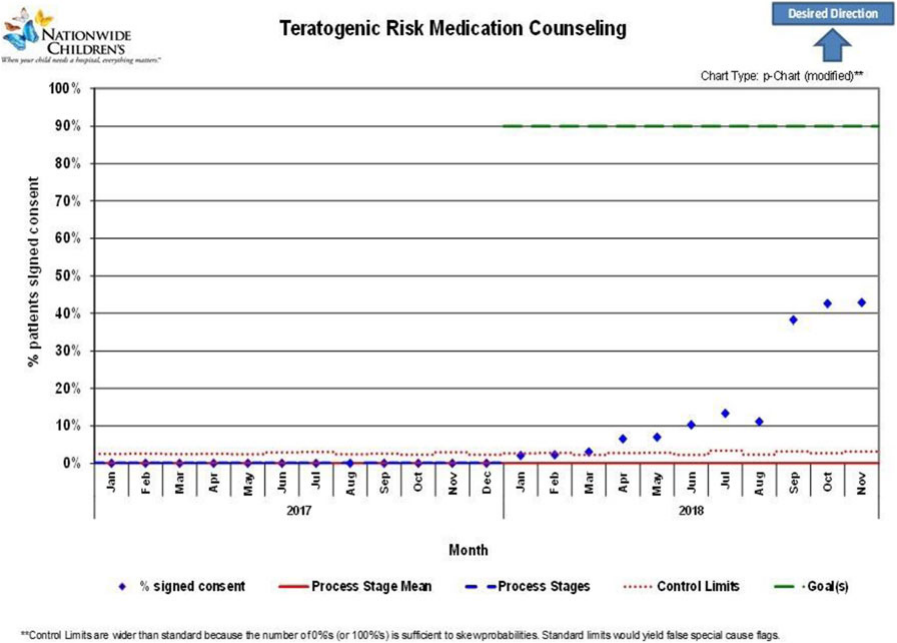
See text for description

## A38 Comparing Biologic Switching Among JIA Patients by Reason for Discontinuation: A Cohort Study in the Childhood Arthritis and Rheumatology Research Alliance Registry

### Melissa Mannion^258^, Fenglong Xie^258^, Daniel Horton^203^, Sarah Ringold^206^, Colleen Correll^281^, Anne Dennos^95^, Timothy Beukelman^258^, for the CARRA Registry Investigators

#### ^258^University of Alabama at Birmingham, Birmingham, AL, USA; ^203^Rutgers University, New Brunswick, NJ, USA; ^206^Seattle Children's Hospital, Seattle, WA, USA; ^281^University of Minnesota, Minneapolis, MN, USA; ^95^Duke University, Durham, NC, USA

##### **Correspondence:** Melissa Mannion

Background : Treatment of JIA has changed significantly with the availability of biologic medications. Recent recommendations suggest adjusting therapy, including switching biologics if necessary, to achieve inactive disease. Because biologics with different mechanisms of action, administration, and side effect profiles are available, individuals may switch treatments due to reasons other than inefficacy. The rates and types of switching by reason for switching in clinical practice is currently unknown.

Methods : We used the Childhood Arthritis & Rheumatology Research Alliance (CARRA) Registry of clinical data from >60 pediatric rheumatology clinics in the United States and Canada. Individuals with JIA were included if they newly started a biologic medication after January 1, 2008 and had a minimum of 12 months of observable time following medication start. We excluded individuals with systemic JIA. Subjects were sorted by reason for discontinuation of the first biologic into 6 categories: ineffective/disease flare, adverse event, infusion/injection reaction, mild adverse event/intolerance, other, or unknown. The characteristics and pattern of switching between those who stopped for ineffective/disease flare and all other reasons were compared by chi-square for categorical variables and Wilcoxon rank sum for continuous variables.

Results : There were 1361 children with JIA who newly started a biologic medication, of whom 349 (26%) were switchers. The majority of patients had started on a TNF inhibitor (1276, 94%), specifically etanercept (871, 64%). Of the biologic switchers, ineffective/disease flare was the most common reason for switch (202, 58%) (Table 1). The most common type of switch was from etanercept to another TNF inhibitor (221, 63%). Subjects switched sooner for ineffective/disease flare (median 306 days) compared to all other reasons (median 660 days). There were 105 subjects who switched from a 2nd to 3rd biologic, most of whom switched for ineffectiveness/disease flare (79, 75%). However, the most common type of second switch was from a TNF inhibitor other than etanercept to a non TNF inhibitor (53, 50%).

Conclusions : In a multicenter cohort of JIA patients who started a biologic medication, 26% switched to a second biologic and of those 30% switched to a third biologic. The most common first switch was from etanercept to another TNF inhibitor. Ineffectiveness/disease flare was the most common reason for discontinuing both the first and second biologic medication. Additional studies are needed to evaluate the outcomes following biologic switching, to identify the optimal timing of switching, and the preferred second-line agent.

Acknowledgments: Support for this work was funded by a CARRA – Arthritis Foundation Grant. This work could not have been accomplished without the aid of the following organizations: The NIH’s National Institute of Arthritis and Musculoskeletal and Skin Diseases (NIAMS) & the Arthritis Foundation. We would also like to thank all participants and hospital sites that recruited patients for the CARRA Registry.

The study was approved by University of Alabama's Institutional Review Board, protocol number X170112004.

**Table 1 (abstract A38).** Characteristics of biologic switchers in the CARRA Registry

	All 1^st^ Biologic Switchers (N = 349)	Switch for Inefficacy/Disease Flare n= 202	Switch for all other reasons n=147	p value
Reason for switch: n, %				xx
Adverse event	11 (3)	0	11 (7)	
Ineffective/Disease Flare	202 (58)	202 (100)	0	
Infusion/Injection reaction	7 (2)	0	7 (5)	
Mild AE/Intolerance of Delivery mode	32 (9)	0	32 (22)	
other	66 (19)	0	66 (45)	
Unknown/Incomplete data	31 (9)	0	31 (21)	
Age (years) median, IQR	10 (7-13)	11 (8-13)	9 (6-13)	0.0099
Female (N, %)	274 (79)	157 (78)	117 (80)	ns
Time from start medication to switch (start 2^nd^) (days) median, IQR	394 (192-891)	306 (175-633)	660 (252-1233)	<0.0001
Elapsed time from discontinuation of 1^st^ biologic to start of 2^nd^ biologic (days) median, IQR	0 (0-21)	0 (0-7)	1 (0-80)	<0.0001
Calendar year of switch: median, range, IQR	2016 (2015-2017)	2016 (2015-2017)	2016 (2014-2017)	ns
Type of switch: n,%				0.03
Etanercept to other TNF	221 (63)	130 (64)	91 (62)	
Etanercept to non TNF	33 (10)	19 (9)	14 (10)	
Other TNF to Etanercept	28 (8)	10 (5)	18 (12)	
Other TNF to non TNF	21 (6)	16 (8)	5 (3)	
Non TNF to Etanercept	7 (2)	1 (1)	6 (4)	
Non TNF to other TNF	4 (1)	3 (1.5)	1 (1)	
Non TNF to non TNF	5 (1)	3 (1.5)	2 (1)	
Other TNF to other TNF	30 (9)	20 (10)	10 (7)	

IQR, interquartile range; AE, adverse event; TNF, tumor necrosis factor inhibitor

## A39 Children with sudden onset neuropsychiatric symptoms: inflammation markers, arthritis, enthesitis and concurrent autoimmune/inflammatory diseases

### Avis Chan, Hannah Karpel, Carolyn Herrera, Grace Goodwin, Margo Thienemann, Bahare Farhadian, Theresa Willett, Elizabeth Mellins, Jennifer Frankovich

#### Stanford University School of Medicine, Stanford, CA, USA

##### **Correspondence:** Avis Chan

Background : Immunological factors and familial clustering of autoimmune diseases are increasingly recognized in neuropsychiatric disorders, including obsessive compulsive disorder (OCD) and tic disorders. Pediatric Acute-onset Neuropsychiatric Syndromes (PANS) is defined as sudden and severe onset of OCD and/or eating restriction with at least two other sudden onset neuropsychiatric symptoms. This study presents rates of co-existing arthritis, concurrent autoimmune/inflammatory diseases, blood dyscrasias, abnormal levels of autoimmune markers and complements, and signs (laboratory and physical exam) relating to small vessel inflammation among a cohort of patients with PANS.

Methods : We reviewed records of 150 study participants who: meet PANS criteria, have at least 3 clinic visits, and live within 90 miles of the Stanford PANS clinic. We ran descriptive statistics and used a linear mixed model to study the association between levels of immune/vasculitis markers (C4a, C4, D-dimer, or vWF Antigen) and the patient’s Global Impairment (GI) score (a validated caregiver or patient--reported measure of symptom severity) to account for repeated measures, sex, age, time-in-clinic, pain and fatigue.

Results : 89/150 (59%) are male and 130/150 (87%) are non-Hispanic Caucasians. The mean age of neuropsychiatric symptom onset is 8.5 years. Table 1 shows prevalence rates of concurrent autoimmune/inflammatory diseases and blood dyscrasias; 85% of autoimmune diseases are detected after neuropsychiatric symptom onset. Tables 2 and 3 present the proportions of patients with abnormal levels of autoimmune and complement activation markers at illness presentation, as well as patients with physical signs/laboratory results suggestive of small vessel inflammation. Linear mixed modeling reveals each 1000 ng/mL increase in C4a is associated with one-point increase in the GI score (p=0.02) and the result remains robust after adding pain and fatigue to the model.

Conclusions : The rate of arthritis in our cohort is higher by > 100 fold than the general population while the rate of autoimmune diseases is higher by at least 3 fold. We also observe a high prevalence of abnormal autoimmune markers and vasculitis markers in our PANS cohort. Our finding of a positive association between C4a levels with the GI score suggests a potential role of inflammation in psychiatric symptoms.

The study was approved by the Stanford University’s Internal Review Board, protocol 26922. This institution is in compliance with requirements for protection of human subjects, including 45 CFR 46, 21 CFR 50 and 56, and 38 CFR 16.

**Table 1 (abstract A39).** Concurrent inflammatory/ autoimmune diseases and blood dyscrasia

	N (%)
*Arthritis*	
Any arthritis below^a^	63/150 (38.8%)
Enthesitis Related Arthritis	32/150 (22.0%)
Psoriatic Arthritis	10/150 (6.7%)
Spondyloarthritis	22/150 (14.7%)
Axial	5/150 (3.3%)
Peripheral	18/150 (12.0%)
Transient or Reactive Arthritis	9/150 (6.0%)
*Autoimmune Disease*	
Any autoimmune disease	26/150 (17.3%)
Autoimmune thyroiditis	16/150 (10.7%)
Celiac disease	5/150 (3.3%)
Psoriasis	7/150 (4.7%)
Chronic urticaria	3/150 (2.0%)
Behcet's disease	3/150 (2.0%)
Antiphospholipid syndrome	1/150 (0.7%)
Type 1 diabetes	1/150 (0.7%)
Inflammatory bowel Disease	2/150 (1.3%)
*Other Inflammatory Disease*	
Eosinophilic esophagitis (EoE)	3/150 (2.0%)
Periodic fever syndrome	1/150 (0.7%)
*Blood Dyscrasia*	
Leukopenia*	21/150 (14.0%)
Lymphopenia*	19/150 (12.7%)
Thrombocytopenia*	4/150 (2.7%)
Thrombocytosis	10/150 (6.7%)
Monocytosis	79/150 (52.7%)
Iron deficiency anemia^b^	9/60 (15.0%)
Low mean corpuscular volume (MCV)^b^	25/111 (22.5%)
Low ferritin^b^	34/79 (43.0%)

^a^More than one type of arthritis can exist in a patient due to overlapping criteria

^b^Denominators reflect number of patients who had the specific laboratory parameter evaluated

*Clinical criteria as per SLICC criteria for systemic lupus erythematosus

**Table 2 (abstract A39).** Autoimmune/ inflammation markers and complement activation markers at first presentation to our clinic and within four months of psychiatric symptom onset

Autoimmunity Marker	N (%)
High Anti-Histone Antibody*	21/130 (16.2%)
High Anti-Thyroglobulin Antibody*	21/100 (21.0%)
High Thyroid Peroxidase Antibody *	15/104 (14.4%)
Positive Anti-Nuclear Antibody (>1:320)	34/138 (24.6%)
Elevated C1Q Binding Assay*	30/89 (33.7%)
Low C4**	27/73 (37.0%)
Elevated C4a*	51/73 (69.9%)

Denominators reflect number of patients who had the specific laboratory parameter evaluated

Abbreviations: MCV = Mean Corpuscular Volume

*Above laboratory reference range

**Outside age/sex-adjusted reference ranges

**Table 3 (abstract A39).** Indirect signs and markers of systemic vascular injury and/ or inflammation

Marker	N (%)
*Physical Exam Findings*	
Livedo Reticularis	82/150 (54.7%)
Mild/unspecified	53/82 (64.6%)
Moderate/prominent	29/82 (35.4%)
Terry's Nails	81/150 (54.0%)
Periungual Redness	66/150 (44.0%)
Palatal Petechiae	65/150 (43.3%)
Elevated Von Willebrand Factor Antigen^a^	14/70 (20.0%)
Elevated D-Dimer^a^	8/68 (11.8%)
Elevated C-reactive protein (CRP)^b^	7/94 (7.4%)
Elevated erythrocyte sedimentation rate (ESR)^b^	14/82 (17.1%)

Denominators reflect number of patients who had the specific laboratory parameter evaluated

^a^At the time of illness presentation (defined as within 4 months of psychiatric symptom onset)

^b^During the first flare of neuropsychiatric symptoms in our clinic

## A40 Musculoskeletal Ultrasound Study In Childhood Arthritis a Limited Examination – MUSICAL - Examination

### Patricia Vega-Fernandez^100^, Johannes Roth^283^, Edward Oberle^154^, Tracy Ting^66^ for the CARRA Investigators

#### ^100^Emory University, Atlanta, GA, USA; ^283^University of Ottawa, Ottawa, ON, Canada; ^154^Nationwide Children's Hospital / The Ohio State University, Columbus, OH, USA; ^66^Cincinnati Children's Hospital, Cincinnati, OH, USA

##### **Correspondence:** Patricia Vega-Fernandez

Background : Juvenile Idiopathic Arthritis (JIA) is the most common rheumatologic disease of childhood. Most of the core set of assessment measurements are subjective by nature. A validated, accessible tool that can evaluate disease response is lacking. Musculoskeletal Ultrasound (MSUS) is a non-invasive, efficient, well accepted imaging tool capable of being used at the bedside by a trained ultrasonographer for the assessment of inflammatory arthritis. MSUS is known to have better sensitivity and reliability to detect synovitis than clinical examination. The aims of this study are 1. Determine the minimum number of joints needed to assess MSUS-evidenced disease activity in JIA; 2. Evaluate feasibility and acceptability from both patient/parent and clinician experience of a limited MSUS.

Methods : JIA patients presenting with an active joint count >4 without recent intraarticular corticosteroid injection in the past month, able to perform first visit within 1 week of starting a Disease-modifying Antirheumatic Drug (DMARD) are eligible for this study. A total of 30 pts will be enrolled for this pilot study. In addition to general demographic and clinical data, a comprehensive clinical physical examination and a 44 joint MSUS examination by an American College of Rheumatology Musculoskeletal Ultrasound (ACR RhMSUS) certified pediatric rheumatologist will be performed at baseline and at 3 months. Determination of a limited joint examination will be made by a data reduction process to detect at least 90% synovitis within the comprehensive exam.

Results : For a 44 joints MSUS examination a total of 248 views per patient were identified. To assure standardization during the process of imaging collection and scoring the authors have developed an Image Acquisition Manual and an Image Scoring Manual. After two calibration exercises addressing imaging acquisition and scoring acceptable reliability was established for all joints but the shoulder. A total of 25 patients have been enrolled (8 males and 17 females) and 16 patients have completed both study visits. Most of the patients (60%) have polyarticular RF negative JIA or polyarticular RF positive JIA (24% of patients). Patient mean age is 13 years of age (ranging from 5-17 years of age). Scoring exercise of acquired images is underway. We anticipate completing the study in the next 4-6 months.

Conclusions : A limited MSUS examination will help determine the role of MSUS as a diagnostic and prognostic instrument in pediatrics. It will improve clinical assessment of disease activity in JIA and strengthen medical decision making.

This study was approved by the Institutional Review Board at Emory University, Cincinnati Children’s Hospital Medical Center, Nationwide Children’s Hospital, and University of Ottawa.

Acknowledgements: This project was funded by a CARRA – Arthritis Foundation Grant. The authors wish to acknowledge the ongoing Arthritis Foundation financial support of CARRA.

## A41 Prevalence of Pain Amplification and Fibromyalgia among Pediatric Patients with Abrupt-onset Psychiatric Symptoms

### Avis Chan, Collin Leibold, Margo Thienemann, Matthew Sigurdson, Kristin Sainani, Kayla Brown, Shirley Chen, Bahare Farhadian, Theresa Willett, Elliot Krane, Jennifer Frankovich

#### Stanford University, Stanford, CA, USA

##### **Correspondence:** Avis Chan

Background : In 2012, we established a combined rheumatology-psychiatry clinic to further understand the relationship between inflammation, rheumatological symptoms, and psychiatric symptoms and to treat patients in an integrated manner. After observing a high rate of pain, fatigue, and sensory disturbances among patients meeting criteria for Pediatric-onset Neuropsychiatric Syndrome (PANS), we initiated a prospective study to evaluate and track pain in this patient population using the revised American College of Rheumatology fibromyalgia tool (ACR fibromyalgia tool) (Wolfe 2016).

Methods : Through a parent/patient questionnaire, we prospectively collected data which compose the ACR fibromyalgia at each clinic visit. We compared clinical characteristics in patients with PANS and healthy controls, also in patients with and without pain amplification. We ran correlation analyses of clinical variables with the ACR fibromyalgia tool scores.

Results : 109 patients (living within 90 miles of our clinic) meet criteria for this study (age 4-18 years, meet PANS criteria, and were seen for >1 year). More patients with PANS compared to controls reported pain, fatigue, sensory abnormalities and exercise intolerance (Table 1). Pain amplification syndromes were diagnosed by a rheumatologist in 47 of 109 patients. The risk of development peaks at time of PANS onset (8.5 years). Eight patients developed pain amplification prior to PANS onset (Figure 1A). In 39 of 47 cases, it lasted > 3 months; in 18 of 47 cases, it eventually remitted (Figure 1B). Most PANS disease severity variables are positively correlated with the ACR fibromyalgia tool. The most highly correlated variables are pain, PANS Global Impairment Score and “cognitive symptoms” as defined by the ACR fibromyalgia tool. Patients with pain amplification are more likely to have a chronic/static course of their PANS illness and objective evidence of enthesitis +/-arthritis (Table 2). They also had higher PANS Global Impairment Scores and Caregiver Burden Inventory at clinic entry but had faster decreases in those scores than patients without pain amplification.

Conclusions : Pain, fatigue, exercise intolerance, sensory disturbances and fibromyalgia are prevalent among patients with PANS and are associated with chronic/static course of PANS, arthritis, high caregiver burden and functional impairment.

The study was approved by the Stanford University’s Internal Review Board, protocol 26922. This institution is in compliance with requirements for protection of human subjects, including 45 CFR 46, 21 CFR 50 and 56, and 38 CFR 16.

**Table 1 (abstract A41).** Prevalence of pain, fatigue, sensory abnormalities, exercise intolerance, and fibromyalgia in 107 patients with PANS compared with 70 controls without psychiatric symptoms

Variable	Patient reported ever	Patient reported for ≥ 3 months	Control reported
Any pain not due to injury, N (%)	98 / 107 (92%)	73 / 107 (68%)	24 / 70 (34%)
Location of pain, N (%)			
Limb	83 / 107 (78%)	38 / 107 (36%)	2 / 70 (3%)
Upper back	53 / 107 (50%)	20 / 107 (19%)	0 / 70 (0%)
Lower back	62 / 107 (58%)	25 / 107 (23%)	0 / 70 (0%)
Neck	67 / 107 (63%)	26 / 107 (24%)	0 / 70 (0%)
Jaw	35 / 107 (33%)	8 / 107 (7%)	0 / 70 (0%)
Chest	47 / 107 (44%)	12 / 107 (11%)	0 / 70 (0%)
Abdomen	89 / 107 (83%)	51 / 107 (48%)	10 / 70 (14%)
Headaches	91 / 107 (85%)	59 / 107 (55%)	18 / 70 (26%)
Moderate or severe symptoms reported on the fibromyalgia ACR screening tool, N (%)			
Daytime fatigue	80 / 107 (75%)	43 / 107 (40%)	0 / 70 (0%)
Waking unrefreshed	82 / 107 (77%)	45 / 107 (42%)	0 / 70 (0%)
Cognitive symptoms	85 / 107 (79%)	41 / 107 (38%)	1 / 70 (1%)
Exercise intolerance, N (%)	64 / 107 (60%)	28 / 107 (26%)	0 / 70 (0%)
Sensory disturbances, N (%)	69/107 (65%)	48/107 (45%)	2/70 (3%)
Cold intolerance	36 / 107 (34%)	10 / 107 (9%)	2 / 70 (3%)
Heat intolerance	38 / 107 (36%)	16 / 107 (15%)	1 / 70 (1%)
Noise sensitivity	76 / 107 (71%)	39 / 107 (36%)	0 / 70 (0%)
Light sensitivity	71 / 107 (66%)	35 / 107 (33%)	1 / 70 (1%)
Smell/taste sensitivity	64 / 107 (60%)	28 / 107 (26%)	2 / 70 (3%)
Touch/texture sensitivity	66 / 107 (62%)	28 / 107 (26%)	1 / 70 (1%)
Shooting or burning pain	33 / 107 (31%)	8 / 107 (7%)	1 / 70 (1%)
Numbness	17 / 107 (16%)	1 / 107 (1%)	0 / 70 (0%)

**Table 2 (abstract A41).** Associations between pain amplification and demographic and neuropsychiatric disease characteristics in patients with PANS

Variable	Pain amplification	No pain amplification	p-value^a^
Male, N (%)	30 / 47 (64%)	40 / 62 (65%)	1.0
Age (years) at PANS onset, Mean (standard deviation)	8.5 (3.4)	8.7 (3.6)	0.8
Years between onset and 1^st^ clinic visit, Median (interquartile range)	1.6 (0.4 to 3.6)	0.8 (0.2 to 2.3)	0.3
Peak myofascial tender points, Median (interquartile range)	9.0 (5 to 17)	2.0 (0 to 5.5)	<.0001
Diagnosis of arthritis, N, %	28 / 47 (56%)	15 / 54 (28%)	0.001
Chronic/static course, N (%)	20 / 47 (43%)	10 / 62 (16%)	0.002
Fibromyalgia diagnosis (ACR criteria), N (%)	13 / 47 (28%)	3 / 62 (5%)	0.001
ACR fibromyalgia tool score, Median (IQR)	10 (8 to 13)	5 (2.5 to 8)	<.0001

^a^T-test for normally distributed data, Wilcoxon rank sum test for non-normally distributed data (time between onset and first clinic appointment, and peak myofascial tender points), chi square test for categorical data, Fisher’s exact test for categorical data that violate chi square assumptions.

Note: Eight patients were not assessed for arthritis

**Fig. 1 (abstract A41).**
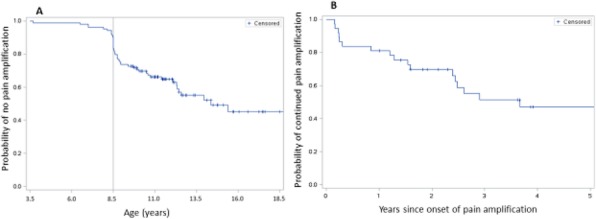
(A) Time-dependent risk of developing pain amplification in a cohort of patients with PANS: patients who were never diagnosed with pain amplification were censored at their last follow-up visit. (B) Rate of remittance from pain amplification syndrome: More rapid resolution of pain amplification is observed in the weeks following initial diagnosis; after three months, the rate of remittance slows. Patients who have not yet remitted were censored at their last follow-up visit

## A42 Patients Advocates and Rheumatology Teams Network for Research and Service: Researchers and Patient Families Working Together To Conduct Research That Matters, To You

### Emily von Scheven^244^, Judy Barlin^139^, Nisha Datta^94^, Mitali Dave^76^, Vincent Del Gaizo^41^, Guy Eakin^19^, Amy Fan^94^, Ewo Harrell^139^, Andew Heaton^76^, Melanie Kohlheim^181^, Sophie Le^94^, Renee Leverty^94^, Sarah Mabus^22^, Laura Marrow^19^, James Minow^76^, Esi Morgan^275^, Marc Natter^30^, Laura Noonan^22^, Anne Paul^275^, C. Rabinovich^95^, Marcela Riano^179^, Laura Schanberg^95^, Suzanne Schrandt^19^, Karin Tse^139^, Angela Young^191^

#### ^244^University of California, San Francisco, San Francisco, CA, USA; ^139^Lupus Foundation of America, Washington, DC, USA; ^94^Duke Clinical Research Institute, Durham, NC, USA; ^76^Cure JM, Baltimore, MD, USA; ^41^CARRA Inc., Milwaukee, WI, USA; ^19^Arthritis Foundation, Atlanta, GA, USA; ^181^Patient Representative; ^22^Atrium Health, Charlotte, NC, USA; ^275^University of Cincinnati College of Medicine, Cincinnati, OH, USA; ^30^Boston Children’s Hospital, Boston, MA, USA; ^95^Duke University, Durham, NC, USA; ^179^Parent Representative; ^191^ Pediatric Rheumatology Care and Outcomes Improvement Network (PR-COIN)

##### **Correspondence:** Emily von Scheven

Background : The PARTNERS Patient Powered Research Network is a consortium of five pediatric rheumatology organizations that was formed to promote patient-centered pediatric rheumatology research. Our vision is to improve the lives of children with rheumatic diseases through research that matters to YOU. The component organizations include Childhood Arthritis and Rheumatology Research Alliance (CARRA), The Pediatric Rheumatology Care and Outcomes Improvement Network (PR-COIN), the Arthritis Foundation (AF), Lupus Foundation of America (LFA), and CureJM.

Methods : Stakeholders (component organizations and patient families) are represented throughout the organization and operating procedures reflect the position that patients and their caregivers bring unique expertise to the research team. The Outreach Communication and Training sub-committee develops patient family and investigator training. Patient training is designed to empower, engage, and educate. Onboarding begins with one-to-one relationship building. Educational materials include online videos and written information. Opportunities for engaged families include participation at the CARRA Annual Scientific Meeting (ASM), research project participation and writing team membership. Investigator training included webinars and presentations at the CARRA ASM. The Research committee provides support to investigators for patient-engagement activities. The Ethics and Data Share committee provides guidance on legal and ethical issues related to privacy, data sharing/ownership and intellectual property.

Results : Over 500 patients and caregivers have expressed interest in participating in research by registering for PARTNERS (25% to complete online surveys, 22% to participate in research studies, 16% to participate in focus groups and 14% to serve on PARTNERS committees). Thirty patient volunteers attended the 2017 CARRA ASM and over 50 attended in 2018, bringing valuable coproduction to the meeting. Nineteen investigators have applied to the Research Committee (15 approved, 1 under review) for assistance with recruiting and training patient families for focus groups, dissemination of surveys and translating materials into lay language. Patient volunteers assumed leadership roles in the LIMIT JIA clinical trial where they lead the patient engagement core. PARTNERS is piloting a Learning Health System which aims to integrate CARRA research and PR-COIN quality improvement expertise to speed up discovery and return of research learnings to point of care. Through mutual respect, comprehensive training, bi-directional learning, and transparency, patients and researchers are successfully working side-by-side.

Conclusions : The culture of pediatric rheumatology research has changed, the inclusion of patient and family investigators in the research process is becoming the norm. The PARTNERS infrastructure supports patient-centered research and improves the relevance and success of research conducted in the pediatric rheumatology community. Next steps include scaling up the recruitment and training of patient partners, marketing investigator supportive services through an online presence on the CARRA website and ongoing activities at the CARRA ASM.

## A43 Parent Perspectives on Addressing Emotional Health for Youth with Juvenile Myositis: A Qualitative Focus Group Study

### Kaveh Ardalan^14^, Tosin Adeyemi^55^, Dawn Wahezi^9^, Anne Caliendo^168^, Megan Curran^51^, Jessica Neely^267^, Susan Kim^267^, Colleen Correll^281^, Emily Brunner^58^, Andrea Knight^229^

#### ^14^Ann & Robert H. Lurie Children's Hospital, Chicago, IL, USA; ^55^Children's Hospital of Philadelphia, Philadelphia, PA, USA; ^9^Albert Einstein College of Medicine, Children's Hospital at Montefiore, Bronx, NY, USA; ^168^Northwestern University Feinberg School of Medicine, Chicago, IL, USA; ^51^Children's Hospital of Colorado, Aurora, CO, USA; ^267^University of California, San Francisco, San Francisco, CA, USA; ^281^University of Minnesota, Minneapolis, MN, USA; ^58^Children's Hospital of Pittsburgh, Pittsburgh, PA, USA; ^229^The Hospital for Sick Children, Toronto, ON, Canada

##### **Correspondence:** Kaveh Ardalan

Background : While juvenile myositis (JM) can negatively affect quality of life, studies of the emotional health needs of youth with JM are limited. We examined parent perspectives on emotional health needs and interventions for youth with JM.

Methods : Six focus groups (60 minutes each, audio-recorded, transcribed verbatim) of parents of youth ages 6-21 years old with JM were conducted at the 2018 CureJM National Family Conference. Parents were grouped by their child’s age (6-12yo, 13-17yo, 18-21yo), with 2 focus groups per age range. Note takers in each focus group assisted with documentation of nonverbal cues by participants. At the start of each focus group, parents listed 3 words/phrases encapsulating JM-related emotional health challenges, which were then discussed in detail. Screening approaches and desired interventions were discussed. Using an a priori coding scheme, interview transcripts were independently coded using the constant comparison method and Dedoose software, with subanalysis by age group. Preference for emotional health interventions was also assessed via Likert scale (1 = very undesirable, 5 = very desirable).

Results : Demographics are shown in Table 1. Parents commonly reported depression/anxiety as challenges, though strength/resiliency also emerged especially in older groups (Figure 1). Themes from the focus groups related to emotional challenges included impact of JM diagnosis, impact of JM on siblings/family dynamic, resiliency, and parental emotional health. Themes related to screening/intervention included perceptions that youth with JM may not always openly report distress, barriers to treatment (e.g. lack of access), desired interventions (e.g. technological modalities, peer support), and provider roles. Parents strongly desired counseling and peer support groups for their children with JM (Figure 2).

Conclusions : Youth with JM and their family members experience substantial emotional challenges, with limited access to interventions. Resiliency in older age groups suggests coping may occur over time. Further studies should assess youth perspectives on emotional health in JM, as well as preferences for and efficacy of interventions.

The study was approved by Children’s Hospital of Philadelphia Institutional Review Board (IRB 18-015225).

**Table 1 (abstract A43).** Parent demographics and reported child characteristics

Parents (n = 45)	
Female, n (%)	38 (84%)
Race/Ethnicity, n (%)	
• White	43 (96%)
• Non-White*	2 (4%)
• Hispanic/Latino	4 (9%)
**Child** (n = 39)**	
Female, n (%)	29 (74%)
Mean age, years (SD)	13.7 (4.6)
Age group, n (%)	
• 6 to 12 years old	17 (44%)
• 13 to 17 years old	13 (33%)
• 18 to 21 years old	9 (23%)
Mean age disease onset, years (SD)	7.9 (4.6)
Mean disease duration, years (SD)	5.8 (4.7)

*non-white included: Asian (n = 1), other (n = 1); youth Asian (n = 1), other (n = 4)

**Six parent couples participated total

**Fig. 1 (abstract A43).**
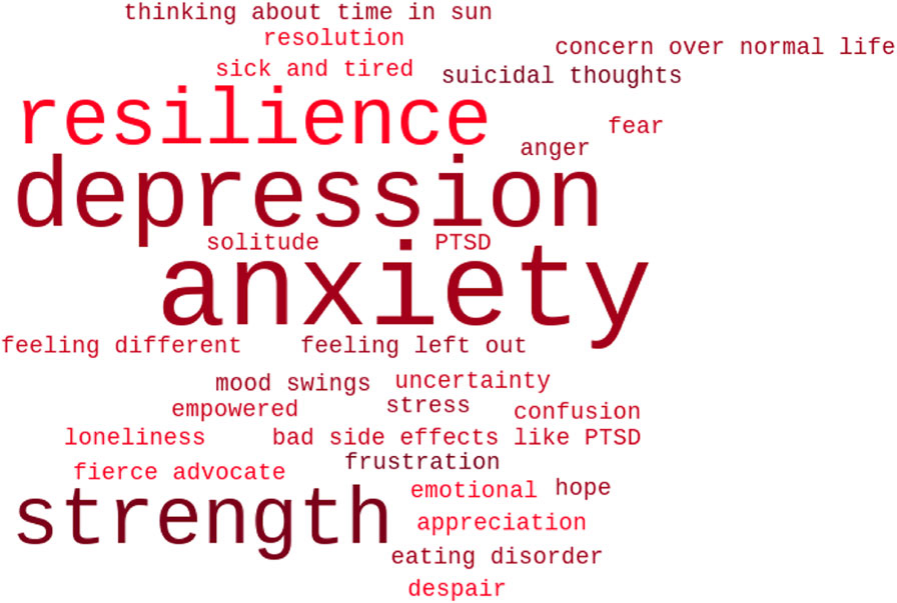
Parent-reported emotional health experiences of youth with juvenile myositis

**Fig. 2 (abstract A43).**
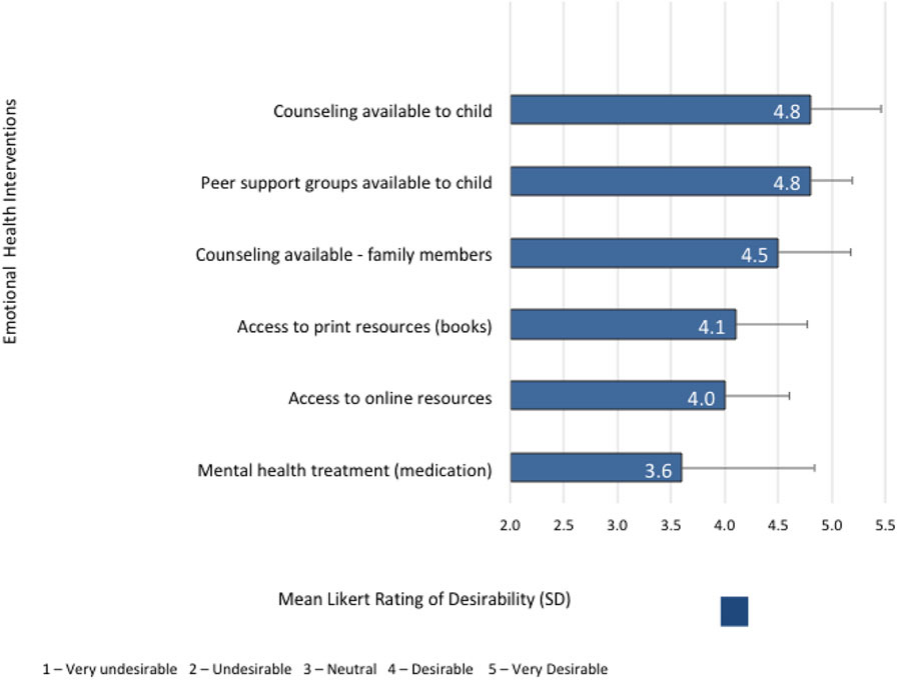
Emotional health interventions desired by parents of young adults with juvenile myositis

## A44 Hypertension and Blood Pressure Variability in Patients with Childhood-Onset Systemic Lupus Erythematosus in the APPLE Trial

### Laura Lewandowski^161^, Stacy Ardoin^174^, Scott Wenderfer^23^, for the APPLE investigators

#### ^161^NIH, Bethesda, MD, USA; ^174^Ohio State University and Nationwide Children's Hospital, Columbus, OH, USA; ^23^Baylor College of Medicine, Houston, TX, USA

##### **Correspondence:** Laura Lewandowski

Background : Hypertension is a frequent manifestation of kidney/vascular involvement in SLE, and in previous reports occurs in 30-70% of childhood onset systemic lupus erythematosus patients (cSLE). Hypertension is an important risk factor for progression to end stage renal disease in SLE patients, particularly pediatric lupus nephritis patients. Blood pressure variability (BPV) is defined as the degree of change between discrete blood pressure (BP) readings separated in time. Increased BPV has been shown to be associated with the development of organ damage in adults and children but has not been studied in a cSLE population. As hypertension is a modifiable risk factor, defining the rates at which cSLE patients present with and develop hypertension is important for understanding risk and guiding management for these patients. In this study, we describe the proportion of patients in the Atherosclerosis Prevention in Pediatric Lupus Erythematosus (APPLE) cohort who present with hypertensive range BP (HBP) or develop HBP and describe the BPV in this cohort. We also characterized differences between those whose BP became hypertensive from those who remained normotensive.

Methods : Demographic and clinical data were analyzed from the APPLE trial database. Blood pressures were reviewed for all baseline and follow up visits for participants in the 3 year APPLE trial. Hypertension was defined as systolic or diastolic blood pressure >95% for age, gender, and height for patients <18 years, and BP > 140/90 in patients older than 18 years. BP measurements were obtained at each study visit. A systolic and diastolic BP index was calculated; BP index ≥1 indicates HBP; every 0.1 U increase represents a 10% increase in BP above hypertensive range. Visit-to-visit BPV was calculated using 2 metrics of systolic and diastolic BPV: (1) standard deviation (SD) and (2) average real variability (ARV) which was calculated as the mean difference in BP between visits.

Results : Table 1. Baseline Characteristics of APPLE subjects (n=221) Table 2. Characteristics of patients with hypertension at baseline and follow up within the APPLE cohort

Conclusions : While only 14% of the APPLE cohort had HBP range at baseline, 45% were found to have HBP at some point in the 3 year study. The BP variability for visit to visit measures was large. Patients who remained normotensive throughout the study tended to have a lower BMI, a shorter duration of SLE, and were less likely to have proliferative LN, compared to those who developed HBP. Risk factors for development of elevated BP, persistent hypertension, and increased BPV and associated outcomes of patients with HTN should be further studied in cSLE patients.

This study was determined to be exempt from IRB review by the NIAMS IRB.

Acknowledgements: The APPLE trial was supported by the NIH (National Institute of Arthritis and Musculoskeletal and Skin Diseases contract N01-AR-2-2265), the Edna and Fred L. Mandel Jr. Center for Hypertension and Atherosclerosis, and Pfizer, which provided atorvastatin and matching placebo.

**Table 1 (abstract A44).** See text for description

Mean age, years (SD)	15.7 (2.6)
Female % (n)	83.3 (184)
Minority status (Hispanic or nonwhite) % (n)	65.2 (144)
BMI, kg/m2 (SD)	24.4 (5.3)
Smoker % (n)	3.2 (7)
Lupus nephritis at baseline % (n)	36.1 (79)
Steroid medication % (n)	81.9 (181)
Prednisone dosage, mean (mg/kg/day)	0.19
Angiotensin-converting enzyme inhibitor (ACEI)	24.4 (54)
History of hypertension	34 (73)
Hypertensive range BP at baseline % (n)	13.6 (30)
Hypertensive range BP ever at follow up % (n)	45.0 (131)
SBP, mean, SD (mm Hg)	112.8 (12.1)
DBP, mean, SD (mm Hg)	66.3 (9.6)
SBP SD (mm Hg)	9.3
DBP SD (mm Hg)	7.4
SBP Index, mean	0.89 (0.10)
DBP Index, mean	0.80 (0.11)
Systolic ARV mean, range (mm Hg)	9.30 (0-26.6)
Diastolic ARV mean, range (mm Hg)	7.33 (2.3-23.4)

Baseline Characteristics of APPLE subjects (n=221)

**Table 2 (abstract A44).** See text for description

	Hypertensive BP at baseline (n=30)	Normotensive BP at baseline (n=191)	Hypertensive BP at follow up (n=131)	Normotensive BP at follow up (n=90)
Minority race % (n)	77 (23)	64 (121)	69.4 (91)	60 (53)
Renal disease % (n)	57 (17)	41 (77)	49.6 (65)	32.6 (29)
Proliferative LN % (n)	23 (7)	26 (50)	33.6 (44)	19 (17)
Mean BMI kg/m2 (SD)	26.3 (6.3)	24 (5.1)	25.2 (5.7)	23.2 (4.4)
Mean duration of SLE, months	27.6	31.7	33.6	26.7
ACEI ever	60 (18)	36 (69)	44 (58)	31 (28)

Characteristics of patients with hypertension at baseline and follow up within the APPLE cohort

## A45 Stopping Medicines for Inactive Juvenile Idiopathic Arthritis: What do Patients and Families Consider?

### Daniel Horton^203^, Jomaira Salas^88^, Aleksandra Wek^141^, Timothy Beukelman^258^, Alexis Boneparth^85^, Jaime Guzman^28^, Ky Haverkamp^299^, Melanie Kohlheim^183^, Melissa Mannion^258^, Lakshmi Moorthy^202^, Elizabeth Stringer^133^, Lori Tucker^28^, Sarah Ringold^206^, Marsha Rosenthal^201^

#### ^203^Rutgers University, New Brunswick, NJ, USA; ^88^Department of Sociology, Rutgers, the State University of New Jersey, New Brunswick, NJ, USA; ^141^Mathematica Policy Research, Princeton, Princeton, NJ, USA; ^258^University of Alabama at Birmingham, Birmingham, AL, USA; ^85^Department of Pediatrics, Columbia University Medical Center, New York, NY, USA; ^28^BC Children's Hospital and University of British Columbia, Vancouver, BC, Canada; ^299^University of Washington School of Medicine, Seattle, WA, USA; ^183^Pediatric Rheumatology Care and Outcomes Improvement Network (PR-COIN), Cincinnati, OH, USA; ^202^Rutgers Robert Wood Johnson Medical School, New Brunswick, NJ, USA; ^133^IWK Health Centre, Dalhousie University, Halifax, NS, Canada; ^206^Seattle Children's Hospital, Seattle, WA, USA; ^201^Rutgers Institute for Health, Health Care Policy and Aging Research, New Brunswick, NJ, USA

##### **Correspondence:** Daniel Horton

Background : Prior research has focused on factors important to clinicians in decisions about withdrawing JIA therapy. Based on recent interviews with patients and caregivers about stopping JIA therapy, we conducted an online survey to study the trade-offs patients and caregivers consider when deciding to stop treatments.

Methods : From June 2017 to February 2018, we conducted an anonymous online survey in English and Spanish using REDCap software. We recruited volunteer participants via social media, email, and flyers in pediatric rheumatology clinics in the U.S. and Canada. Eligible participants were adolescents with JIA (13-17y), adults with JIA (≥18y), or caregivers of children with JIA. Survey questions focused on factors that might influence decisions about stopping JIA treatment. Questions were based on findings from our prior telephone interviews and refined via pilot testing. Analyses used descriptive statistics as well as logistic regression to identify factors associated with respondents’ agreement with doctors’ hypothetical recommendations to withdraw or continue treatment.

Results : 1456 individuals opened the survey and 839 (58%) completed it, including 782 eligible participants (40 adolescents, 120 young adults, and 622 caregivers). A majority of the participants were from the US (93.6%), reported a history of severe JIA flares (84.6%), and used systemic anti-rheumatic medicines (91.9%). Among all groups, damage from JIA was the most highly ranked consideration in deciding whether to stop treatment, but caregivers prioritized this more than patients. Compared to other groups, adolescents prioritized the duration and durability of inactive disease and the impact of JIA or medicines on mood or a sense of normalcy; they ranked harms from medicines as less important. Adults with JIA prioritized harms, cost, and access to medicines more than other groups; trust in doctors was a relatively lower priority for this group. Greater trust in doctors corresponded with agreement with doctors' recommendations to withdraw or continue treatment (adjusted odds ratio [aOR] 2.3-5.4, Tables 1 and 2). Those most concerned about JIA flare or harms from treatment were less likely to agree with recommendations to withdraw (aOR 0.4, 95% CI 0.2, 0.5) or continue (aOR 0.7, 95% CI 0.5, 0.9) treatment, respectively. After accounting for other factors (Tables 1 and 2), patients and caregivers did not appreciably differ in their likelihood to agree with recommendations.

Conclusions : Among survey respondents, JIA-related damage was the top consideration in deciding whether to stop treatments for well-controlled JIA, but patients and caregivers prioritized other considerations differently. Trust in doctors led to more agreement with recommendations, whereas strong concerns about JIA flare or treatment corresponded with risk-averse decision-making. These findings can help inform shared decision-making when considering whether to stop medicines for well-controlled JIA.

The study was approved by the Rutgers University Institutional Review Board, protocol number Pro20160000193. All participants gave consent or, for participants under age 18, assent to participate in the anonymous online survey.

Acknowledgements: Funding from CARRA-Arthritis Foundation, NIH/NIAMS. The authors wish to acknowledge the ongoing Arthritis Foundation financial support of CARRA.

**Fig. 1 (abstract A45).**
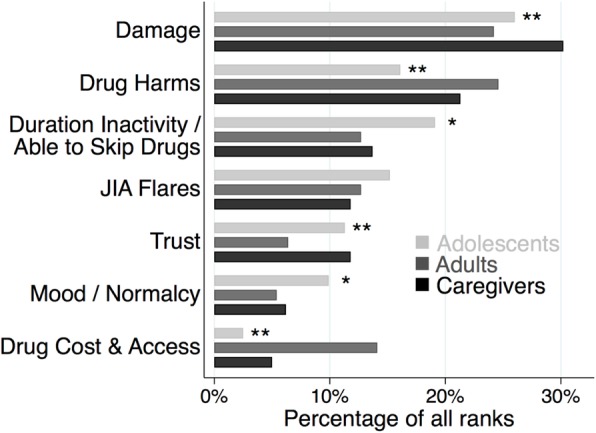
Relative ranking of factors of importance regarding treatment withdrawal

**Table 1 (abstract A45).**
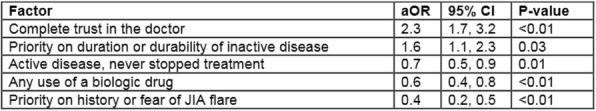
Factors associated with agreement with doctors' hypothetical recommendations to withdraw treatment

**Table 2 (abstract A45).**
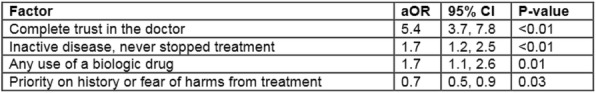
Factors associated with agreement with doctors’ hypothetical recommendations to continue treatment

## A46 Expanded autoantibody testing in pediatric scleroderma

### Kathryn Torok^287^, Emily Mirizio^287^, May Choi^262^, Christopher Liu^288^, Kaila Schollaert-Fitch^287^, Marvin Fritzler^262^

#### ^287^University of Pittsburgh Medical Center, Pittsburgh, PA, USA; ^262^University of Calgary, Calgary, AB, Canada; ^288^University of Pittsburgh, Children's Hospital of Pittsburgh, Pittsburgh, PA, USA

##### **Correspondence:** Kathryn Torok

Background : Scleroderma is a chronic autoimmune disease including 2 main forms: localized scleroderma (LS) and systemic sclerosis (SSc). LS mainly affects the skin and underlying tissues, while SSc affects multiple organ systems. Identification of a disease activity biomarker is vital to accurately assess disease state and assist clinicians with intervening prior to disease progression. The Childhood Arthritis and Rheumatology Research Alliance (CARRA) Scleroderma Foundation Multi-Center Collaborative Research (SCORE) cohort has 2 main goals: 1) Use patient biospecimens to evaluate the predominate circulating phenotype, cytokine/chemokine profile, and autoantibody profile that can predict LS or SSc manifestations, and 2) Merge clinical and laboratory data to develop a composite biomarker for active disease, treatment responsiveness, and predicted organ involvement. Eighteen CARRA centers were identified that could meet enrollment targets. Preliminary autoantibody profiling was performed on the lead center’s pediatric scleroderma cohort to address the proposed aims and establish a statistical methodology template for the inclusion of data from multiple centers.

Methods : Clinical data and sera from 68 pediatric LS, 13 SSc patients, and 46 pediatric healthy controls from the University of Pittsburgh were tested at the University of Calgary for presence of Anti-nuclear antibody (ANA) by indirect immunofluorescence on HEp-2 cells (>1:80) and SSc-related autoantibodies via extractable nuclear antigens (ENA) using the FIDIS™ Connective Profile SSc line immunoassay (LIA) (Euroimmun, Germany) and MagPix® (Luminex™) addressable laser bead immunoassay (ALBIA). Normal cut-offs were determined from healthy pediatric samples (+2 SD) and applied to LS and SSc samples to determine positivity. Chi-square, and when appropriate, Fisher’s exact test were used to determine the relationship between autoantibody results with clinical variables.

Results : Pediatric SSc samples show autoantibodies similar to adult SSc with a prevalence ranging from 14 to 52%. Preliminary data shows these ‘classic’ SSc-associated autoantibodies were also found within pediatric LS patients at a frequency ranging from 6 to 14%, including anti-Scl-70, anti-centromere A, and anti-RNA polymerase (RP11). These correlated (p<0.05) to deep tissue involvement affecting joint mobility, nerve entrapment, and muscular development in LS, and with the modified Rodnan skin score (mRSS) and composite finger flexion measurements in SSc.

Conclusions : Though LS clinical manifestations may be different from SSc, traditional SSc-associated antibodies could potentially classify or predict disease involvement in LS. Additional blood samples and corresponding clinical data from the CARRA SCORE cohort will be analyzed in the future to better characterize these relationships in pediatric LS and SSc.

Samples and clinical data were collected in accordance with the Declaration of Helsinki under University of Pittsburgh IRB# PRO11060222, the National Registry for Childhood Onset Scleroderma. Informed consent was obtained from patients prior to participation.

Acknowledgements: This project was funded by a CARRA-Arthritis Foundation grant. The authors wish to acknowledge CARRA, and the ongoing Arthritis Foundation financial support of CARRA, the Scleroderma Foundation and Taylor Foundation grants.

## A47 A Single Center Experience with Weekly Adalimumab versus Infliximab Therapy in Refractory Uveitis

### Jordan Roberts^33^, Margaret Chang^33^, Peter Nigrovic^35^, Mindy Lo^33^

#### ^33^Boston Children's Hospital, Boston, MA, USA; ^35^Boston Children's Hospital, Brigham and Women's Hospital, Boston, MA, USA

##### **Correspondence:** Jordan Roberts

Background : Adalimumab is currently the only non-corticosteroid therapy approved for the treatment of pediatric uveitis. Although it is approved for every 2 week (Q2 week) dosing, many rheumatologists will increase the frequency to weekly (Q1) dosing if uveitis remains uncontrolled. We sought to assess the effectiveness of this approach compared to switching to infliximab in treating uveitis refractory to Q2 week adalimumab.

Methods : We conducted a retrospective chart review of children with uveitis treated with Q2 week adalimumab who switched to Q1 week adalimumab or monthly infliximab due to persistent uveitis. Patients seen in the Boston Children’s Hospital rheumatology clinic between 2000 and 2018 with an ICD-9 or ICD-10 code of uveitis were included. The primary outcome measure was percent of patients achieving sustained uveitis control. Time to control, frequency of uveitis recurrence, and ocular complications were also collected.

Results : We identified 24 patients with persistent uveitis despite treatment with Q2 week adalimumab who were switched to either Q1 week adalimumab or Q4 week infliximab. Twelve of 19 patients (63%) who were switched to Q1 week adalimumab achieved sustained uveitis control (Figure 1). Of the 7 who did not achieve control with Q1 week dosing, 5 were subsequently controlled on infliximab. Of the 5 patients who were switched from Q2 week adalimumab directly to infliximab, 4 (80%) achieved sustained control (Figure 1). There was no significant difference in percentage of patients achieving control with Q1 week adalimumab versus infliximab (p=0.42), nor in the amount of time required to achieve uveitis control (p=0.16, Figure 2). Infliximab dose needed to achieve control ranged from 5-15 mg/kg. Once controlled, patients averaged 0.21 uveitis flares per patient year on infliximab, compared to 0.24 on weekly adalimumab. Glaucoma occurred in 2 patients (17%) controlled on adalimumab and 3 patients controlled on infliximab (30%) (p=0.62). Cataracts occurred in 2 patients (17%) controlled on adalimumab and 4 patients controlled on infliximab (40%) (p=0.34). No serious infections were noted in any patients.

Conclusions : In our cohort, 63% of patients with refractory uveitis who increased adalimumab to Q1 week dosing achieved uveitis control. This rate was comparable to patients who switched from Q2 week adalimumab to monthly infliximab. Both groups also showed similar time to control, frequency of recurrence, and rate of ocular complications. Further research is needed to demonstrate the safety of Q1 week adalimumab and compare the efficacy of these TNF-inhibitors in larger cohorts.

This study was approved by the Boston Children’s Hospital Institutional Review Board, protocol number P00028620.

**Fig. 1 (abstract A47).**
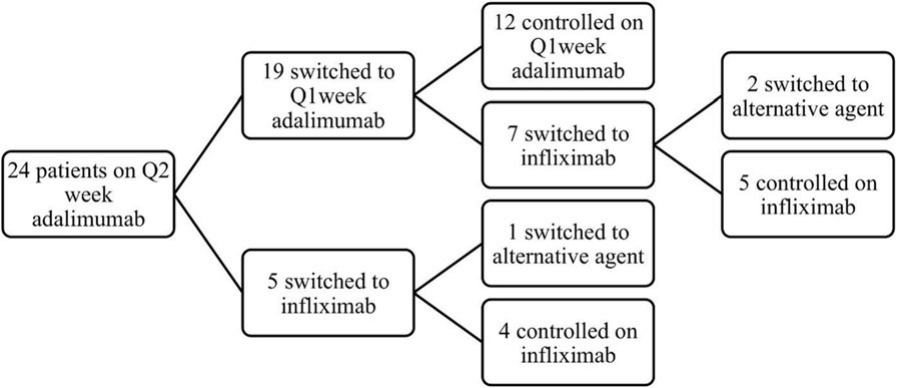
Patient cohort

**Fig. 2 (abstract A47).**
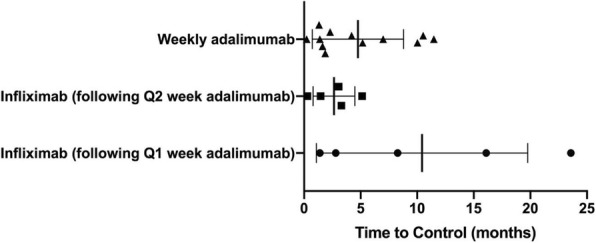
Time to uveitis control following biologic switch

## A48 Anti-thyroid Antibodies Following Subcutaneous Abatacept Treatment in Children and Adolescents With Polyarticular JIA

### Hermine Brunner^193^, Alberto Berman^249^, Francisco Avila Zapata^122^, Ruben Cuttica^124^, John Bohnsack^295^, Maria Alessio^252^, Mara Becker^228^, Alexandre Belot^45^, Ruben Burgos Vargas^123^, Maria Luz Gamir^126^, Iloite Maria Scheibel^112^, Maria Teresa Terreri^250^, Marleen Nys^38^, Robert Wong^38^, Daniel Lovell^193^, Alberto Martini^251^, Nicola Ruperto^194^

#### ^193^PRCSG, Cincinnati Children’s Hospital Medical Center, Cincinnati, OH, USA; ^249^Universidad Nacional de Tucumán, Tucumán, Argentina; ^122^Hospital Galenia, Cancún, Q.R., Mexico; ^124^Hospital General de Niños Pedro, Buenos Aires, Argentina; ^295^University of Utah Health Sciences Center, Salt Lake City, UT, USA; ^252^Università di Napoli Federico II, Naples, Italy; ^228^The Children's Mercy Hospital, Kansas City, MO, USA; ^45^Centre Hospitalier Universitaire de Lyon, Pierre-Bénite, France; ^123^Hospital General de México, Mexico City, Mexico; ^126^Hospital Universitario Ramón y Cajal, Madrid, Spain; ^112^Grupo Hospitalar Conceição, Porto Alegre, RS, Brazil; ^250^Universidade Federal de São Paulo, São Paulo, Brazil; ^38^Bristol-Myers Squibb, New York, NY, USA; ^251^Università di Genoa, Genova, Italy; ^194^PRINTO, Instituto Gaslini, Genova, Italy

##### **Correspondence:** Hermine Brunner

Background : Anti-thyroid antibodies and autoimmune thyroid diseases have been reported in adults with RA, including those receiving biologic therapy, and also in patients with JIA.[1] The association between biologic therapy and thyroid autoimmunity needs further study. Therefore, we measured anti-thyroid autoantibodies and thyroid-stimulating hormone (TSH) in children with polyarticular (p)JIA after 2 years of treatment with abatacept.

Methods : The design of this single-arm, two-cohort (ages 2–5 and 6–17 years), open-label Phase III study (ClinicalTrials.gov, NCT01844518) has been described.[2] Briefly, patients with pJIA received approved doses of SC abatacept weekly for 4 months; JIA-ACR 30% improvement criteria responders at 4 months could receive abatacept for another 20 months. Blood samples for anti-glutamic acid decarboxylase (GAD), anti-thyroid peroxidase (TPO) and TSH (a biomarker of thyroid function) were drawn on Days 1, 113 and every 6 months thereafter, and during a 6-month follow-up period after the last dose of study drug (Days 85 and 168).

Results : A total of 219 patients (2–5 years, n=46; 6–17 years, n=173) were treated. All patients in the 2–5-year-old cohort were negative for anti-GAD and anti-TPO at baseline. In this cohort, positive anti-thyroid antibody values were reported in two patients during the treatment period; no positive values were seen in the follow-up period (Table 1). In the 6–17-year-old cohort, 150/157 (anti-GAD) and 140/149 (anti-TPO) patients were negative at baseline. In this cohort, positive anti-thyroid antibody values (while negative at baseline) were reported in four patients during the treatment period and in only one of these patients in the follow-up period (Table 1). In the 2–5-year-old cohort, no shifts in normal TSH value relative to baseline were seen. In the 6–17-year-old cohort, one patient had elevated TSH at Day 477; subsequent TSH values (Days 645 and 729) were normal. Anti-GAD+ and anti-TPO+ were not associated with any autoimmune thyroid-related events in either cohort.

Conclusions : New anti-thyroid antibodies occurred infrequently in children with JIA following 2 years of abatacept treatment. These antibodies were transient and not associated with hypothyroidism or autoimmune thyroid disease after 2 years of follow-up.

References [1] Kanakoudi-Tsakalidou F, et al. Cytokine 2008;42:293-7. [2] Brunner HI, et al. Arthritis Rheumatol 2018;70:1144-54.

This study was approved by each participating institution's internal review board.

**Table 1 (abstract A48).** Safety Summary

	2 years of abatacept treatment				6-month follow-up period	
	Day 113	Day 477	Day 645	Day 729	Day 85	Day 168
2–5-year-old cohort						
Anti-GAD+	1	1	0	0	0	0
Anti-TPO+	0	0	0	0	0	0
6–17-year-old cohort						
Anti-GAD+	0	0	0	1^‡^	0	0
Anti-TPO+^†^	0	3	1	1^‡^	0	1

Values in table are number of patients. *Autoantibody positivity was defined as anti-GAD ≥5 IU/mL or anti-TPO ≥60 U/mL. TSH abnormalities were defined as >1.5x ULN, or if baseline was >ULN then >2x baseline value. †One patient had new-onset positive anti-TPO antibodies on Days 477, 645, 729 and Day 168 during the follow-up period. ‡The patients with positive anti-GAD and anti-TPO at Day 729 are different patients. GAD=glutamic acid decarboxylase; TPO=thyroid peroxidase; TSH=thyroid-stimulating hormone; ULN=upper limit of normal

## A49 Investigating the Role of Complement in Atherosclerotic Disease in Children and Adults with Systemic Lupus Erythematosus

### Evan Mulvihill^152^, Stacy Ardoin^152^, Scott Wenderfer^23^, Hermine Brunner^193^, Bi Zhou^233^, Logan Welch^12^, Chack-Yung Yu^152^

#### ^152^Nationwide Children's Hospital, Columbus, OH, USA; ^23^Baylor College of Medicine, Houston, TX, USA; ^193^PRCSG, Cincinnati Children’s Hospital Medical Center, Cincinnati, OH, USA; ^233^The Research Institute at Nationwide Children's Hospital, Columbus, OH, USA; ^12^Allegheny University, Meadville, PA, USA

##### **Correspondence:** Evan Mulvihill

Background : Chronic systemic inflammation puts patients with SLE at increased risk for cardiovascular disease (CVD). Using data from the APPLE Trial, we previously demonstrated that children with SLE and hypertension had higher mean serum complement C3 and C4 levels than those without hypertension. They also had a higher mean gene copy number (GCN) of C4B, an allotype of the C4 gene that may be relatively atherogenic. With support from a CARRA Fellows Grant, we aim to replicate those findings in three diverse cohorts of children with SLE and one adult SLE registry. Our first aim is to demonstrate an association between hypertension and C4B GCN. Our second aim is to demonstrate an association between clinical CVD outcomes and C4B GCN in the adult cohort.

Methods : Pediatric SLE data comes from existing patient cohorts at Nationwide Children’s Hospital (NCH), Cincinnati Children’s Hospital Medical Center (CCHMC), and Texas Children’s Hospital. Adult analysis is based on data from the Ohio State University Lupus, Vasculitis, and Glomerulonephritis (LVG) Registry. Blood pressure is classified into one of four standard categories: normal, elevated, stage 1 hypertension, and stage 2 hypertension. TaqMan-based realtime PCR experiments is used to interrogate GCN of C4B, C4A and total C4 for each participant. Two-sample t tests are used to compare demographic, clinical, and genetic data in hypertensive and non-hypertensive patients. Categorical data between groups is compared by χ2 analyses. Once data is complete, stepwise logistic regression will be used to determine risk factors for hypertensive status.

Results : Interim analysis demonstrated that the NCH and CCHMC cohorts are composed largely of female patients who are predominantly white or African American (Table 1). Of the 118 patients in these two cohorts, 33.90% have a diagnosis of lupus nephritis and 33% have a history of obesity. Of those with relevant clinical data available, 18.31% were hypertensive at diagnosis and 33.33% were hypertensive at most recent visit. No significant clinical differences were seen between those with hypertension and those without (Table 2). Serum complement levels at diagnosis and at most recent visit were similar between the two groups as were complement genetics.

Conclusions : This CARRA funded project aims to further investigate the role of complement in hypertension and CVD in patients with SLE. Interim analysis demonstrates no association between complement and hypertension. Next steps in this project are to complete pediatric analysis and to analyze clinical data in the adult registry.

Acknowledgements: This project was supported by Childhood Arthritis and Rheumatology Research Alliance (CARRA)-Arthritis Foundation Fellows Small Grant. The authors wish to acknowledge CARRA, and the ongoing Arthritis Foundation financial support of CARRA.

This study was performed under approved IRB Protocol (IRB18-00339).

**Table 1 (abstract A49).**
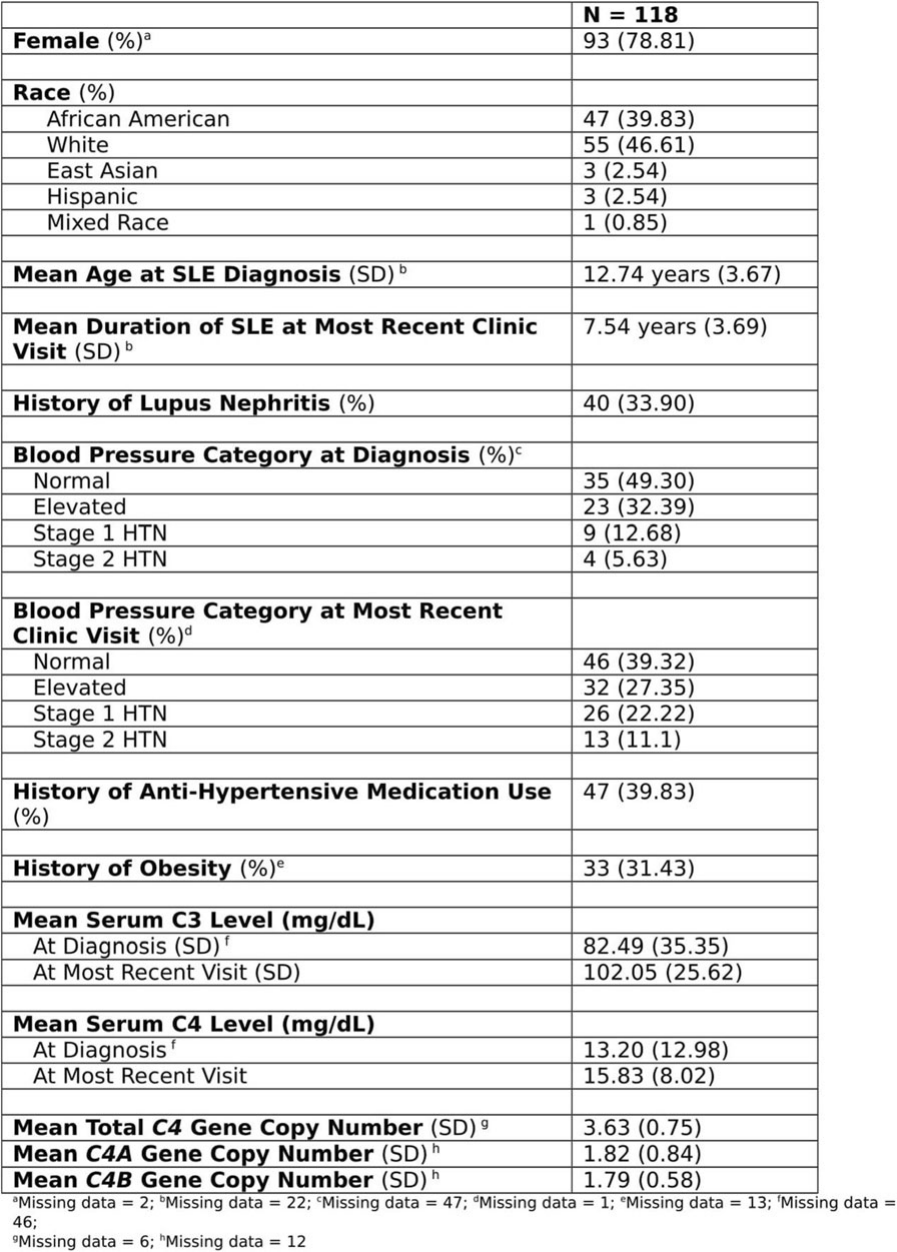
See text for description

Clinical Data from Nationwide Children’s Hospital and Cincinnati Children’s Hospital Medical Center SLE Cohorts

**Table 2 (abstract A49).**
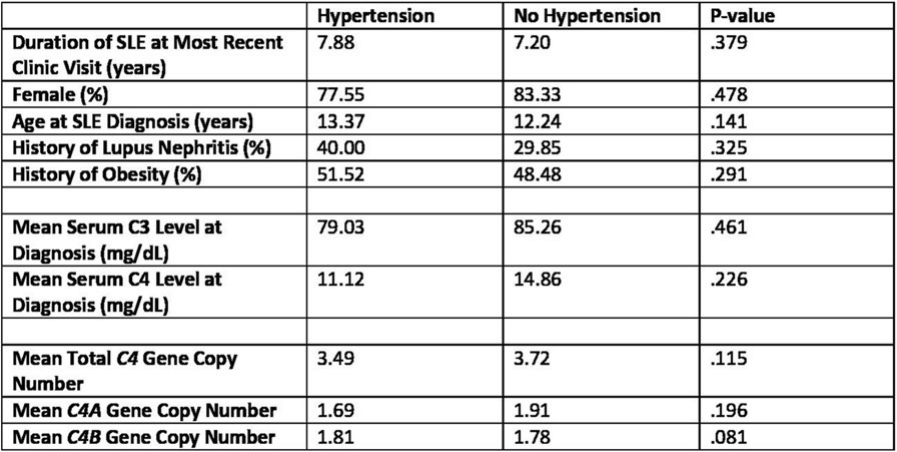
See text for description

Clinical and Genetic Data in Patients With and Without Hypertension

## A50 Management of TMJ Arthritis; Eminence or Evidence

### Jocelyne Beelen^75^, Peter Stoustrup^2^, Thomas Klit Pedersen^4^, Cory M. Resnick^31^, Shelly Abramowicz^102^, Marinka Twilt^91^, for TMJaw and the CARRA TMJ interest group

#### ^75^Cumming School of Medicine, University of Calgary, Calgary, AB, Canada; ^2^Section of Orthodontics, Department of Dentistry and Oral Health, Aarhus University, Denmark; ^4^Aarhus University Hospital, Section of Orthodontics, Aarhus University, Denmark; ^31^Boston Children’s Hospital, Harvard School of Dental Medicine, Harvard Medical School, Boston, MA, USA; ^102^Emory University School of Medicine, Children's Healthcare of Atlanta, Atlanta, GA, USA; ^91^Division Rheumatology, Alberta Children’s Hospital, University of Calgary, Calgary, AB, Canada

##### **Correspondence:** Jocelyne Beelen

Background : The temporomandibular joint (TMJ) is often affected in children with Juvenile Idiopathic Arthritis (JIA), with the occurrence varying widely depending on factors such as JIA type, diagnostic approaches, and study population. [1] Inflammation in the TMJs can result in joint deformity, dysfunction, and substantial morbidity in the pediatric arthritis population. [2] However, management of TMJ arthritis is difficult due to the uniqueness of the joint and need for multidisciplinary care. This study will aim to develop management recommendations based on expert consensus for TMJ arthritis in JIA by involving members of TMJaw and the Childhood Arthritis and Rheumatology Research Alliance (CARRA) TMJ interest group.

Methods : A three-step approach will be used to develop management guidelines for TMJ arthritis in JIA: 1) conduct a systematic review of the available literature on the management of TMJ arthritis in JIA; 2) seek endorsement by TMJaw members, CARRA TMJ interest group members, and international experts on the management recommendations presented in the selected high-quality articles; 3) develop TMJ arthritis management recommendations.

Results : Of the 60 articles selected for full-text review, 18 articles were deemed high-quality from the fields of: dentistry (n=1), imaging analysis and stereology (n=7), rheumatology (n=5), orthodontics (n=2), and oral maxillofacial surgery (n=3). No trials were available and therefore no meta-analysis was possible. Extrapolated evidence suggests that a multidisciplinary approach to care is necessary for diagnosis, interception, and management. Intervention with an orthodontic/orthopedic appliance may provide symptom relief and can minimize or correct developing deformity. Intraarticular TMJ injections generally result in symptom relief but may negatively influence TMJ growth and cause heterotopic bone formation. Joint reconstruction corrects dentofacial deformity and improves function when necessary.

Conclusions : Early diagnosis, monitoring, and treatment is necessary to reduce potential morbidity of TMJ arthritis. A multidisciplinary approach is necessary.

References 1. Pedersen TK, Jensen JJ, Melsen B, Herlin T. Resorption of the temporomandibular condylar bone according to subtypes of juvenile chronic arthritis. J Rheumatol. 2001; 28(9):2109-15. 2. Abramowicz S, Cheon JE, Kim S, Bacic J, Lee EY. Magnetic Resonance Imaging of Temporomandibular Joints in Children With Arthritis. J Oral Maxillofac Surg. 2011; 69(9):2321-2328.

## A51 Developing CTPs for Anti-NMDA Receptor Encephalitis

### Jocelyne Beelen^75^, Marinka Twilt^91^, Heather Van Mater^95^, Marisa Klein-Gitelman^13^, Elizabeth Wells^62^, Susanne Benseler^91^, Dominic Co^303^, Eyal Muscal^26^, for the CARRA Autoimmune Encephalitis Working Group

#### ^75^Cumming School of Medicine, University of Calgary, Calgary, AB, Canada; ^91^Division Rheumatology, Alberta Children’s Hospital, University of Calgary, Calgary, AB, Canada; ^95^Duke University, Durham, NC, USA; ^13^Ann & Robert H. Lurie Children’s Hospital of Chicago, Chicago, IL, USA; ^62^Children's National Health System, Washington, DC, USA; ^303^University of Wisconsin, American Family Children's Hospital, Madison, WI, USA; ^26^Baylor College of Medicine/Texas Children's Hospital, Houston, TX, USA

##### **Correspondence:** Jocelyne Beelen

Background : Anti-N-methyl-D-aspartate receptor (NMDAR) encephalitis is a devastating autoimmune disorder that presents in individuals of all ages. [1] It can result in significant neurological morbidity and even death, however early treatment in the pediatric population has been demonstrated to result in better outcomes. [1,2] No uniform treatment plans are used among health care providers caring for children with NMDAR encephalitis. Therefore one of the aims of the Childhood Arthritis and Rheumatology Research Alliance (CARRA) Autoimmune Encephalitis (AE) working group is to develop CARRA endorsed consensus treatment plans (CTPs) for anti-NMDAR encephalitis in children.

Methods : A three-step approach will be used to develop the CARRA endorsed CTPs for anti-NMDAR encephalitis in children: 1) conduct a systematic review of the available literature on the treatment of anti-NMDAR encephalitis in children; 2) analyze and discuss the treatments and outcomes of the selected high-quality articles to suggest treatment protocols; 3) seek expert consensus and CARRA endorsement; 4) develop AE outcome measurement tools to evaluate treatment response. This abstract shows results for step 1 & 2.

Results : Of the articles screened, 17 articles were deemed high quality. No trials were available and therefore no meta-analysis was possible. The majority of patients received induction treatment with either 1) prednisone and IVIG which was both well-tolerated and effective overall, or with prednisone, IVIG and cyclophosphamide and/or rituximab. Relapse rates were higher in the group treated with prednisone and IVIG alone. Favourable neurological outcome, assessed using modified Rankin scale (mRS) scores were seen in both groups. CTPs for anti-NMDAR encephalitis in children should aim to use induction treatment options including; Prednisone, IVIG with or without a combination of cyclophosphamide and/or rituximab.

Conclusions : Early induction treatment of prednisone, IVIG and/or cyclophosphamide and/or Rituximab are frequently used treatment strategies in NMDAR encephalitis. The CARRA AE working group will develop CTP strategies based on the outcome of the systemic review and expert opinions in the CARRA AE working group and external experts. In addition the AE working group is working on developing an evaluation mechanism for the treatment strategies and building North American pediatric neurology, rehabilitation, and behavioural health (psychiatry, psychology and neuropsychology) AE networks.

References 1. Titulaer MJ, McCracken L, Gabilondo I, Armangué T, Glaser C, Iizuka T, Honig LS, Benseler SM, Kawachi I, Martinez-Hernandez E, Aguilar E, Gresa-Arribas N, Ryan-Florance N, Torrents A, Saiz A, Rosenfeld MR, Balice-Gordon R, Graus F, Dalmau J. Treatment and prognostic factors for long-term outcome in patients with anti-NMDA receptor encephalitis: an observational cohort study. Lancet Neurol. 2013; 12(2):157-65. 2. Byrne S, Walsh C, Hacohen Y, Muscal E, Jankovic J, Stocco A, Dale RC, Vincent A, Lim M, King M. Earlier treatment of NMDAR antibody encephalitis in children results in a better outcome. Neurol Neuroimmunol Neuroinflamm. 2015; 2(4):e130.

Acknowledgements: This study is funded through a CARRA – Arthritis Foundation Grant. The authors wish to acknowledge the ongoing Arthritis Foundation financial support of CARRA.

## A52 Reintroduction of DMARDS and Biologics in Pediatric Rheumatology Patients with Histoplasmosis

### Rachel Brown^230^, Fatima Barbar-Smiley^155^, Monica Ardura^155^, Stacy Ardoin^155^, Shoghik Akoghlanian^155^

#### ^230^The Ohio State University, Columbus, OH, USA; ^155^Nationwide Children's Hospital, Columbus, OH, USA

##### **Correspondence:** Rachel Brown

Background: Children with rheumatic diseases (cRD) receive immunosuppressive medications (IM) that increase risk for acquiring invasive histoplasmosis (IH). Withholding IM during active IH is recommended, but poses risk of rheumatic disease flares. Conversely, reinitiating IM increases risk for IH recurrence. TNFa inhibitor (TNFai) biologics carry the highest risk for acquiring IH, so other treatments are preferred after IH. In this case series, we examine clinical characteristics and outcomes of patients with IH and juvenile idiopathic arthritis (JIA), systemic lupus erythematosus (SLE), or mixed connective tissue disease (MCTD) after reintroduction of IM.

Methods : Retrospective chart review of cRD diagnosed with IH at a large freestanding children’s hospital in the Ohio Valley. Collected data included: demographics, rheumatic diagnosis, imaging, and IH labs, including Histoplasma blood and urine antigens (histAg), antibodies, and lung tissue biopsy if performed. We also noted medications used prior to, during, and following IH infection.

Results : Nine patients met inclusion criteria: 7 (78%) JIA, 1 SLE, & 1 MCTD patients; 7 (78%) females; 7 (78%) Caucasian, 2 (22%) African American. Median age at IH diagnosis was 16 years (5-18). Medications at time of acquiring IH were: 7 (78%) IM (4 TNFai, 6 methotrexate [MTX], 3 hydroxychloroquine [HCQ]), 3 (33%) systemic steroids, and 2 (22%) solely NSAIDs. Median time (MT) from starting IM to IH was 14.5 months (mo) (5-32). At IH diagnosis, IM except HCQ was halted in patients with disseminated disease (histAg+), but not in histAg- patients. Itraconazole was given for a MT of 30 mo (2-56); 5 patients received amphotericin B first. Patients initially used NSAIDs or HCQ to control rheumatic disease during antifungal therapy (AT); 8 patients received intra-articular steroid injections during AT. MT to IH clearance for 7 patients was 13 mo (2-37); 2 patients still remain histAg+ at 11 & 18 mo post-IH diagnosis. In 2 patients who received systemic steroid bursts (SSB), MT to histAg resolution was 37 mo compared with 17 mo in 2 patients who did not receive SSB. Active rheumatic disease led to IM initiation in 7 patients (5 abatacept, 1 MTX & tocilizumab, 1 HCQ) a MT of 15 mo (5-26) after IH diagnosis; 4 of whom did not yet demonstrate complete IH clearance. In these 4 patients, 2 were started on abatacept after a MT of 23 mo, 1 on MTX (11 mo), and 1 on HCQ (6 mo), with 3 clearing their IH to date (1 abatacept-37 months, 1 MTX-37 mo, 1 HCQ-21 mo). No patients had recurrent IH in the MT of 46 mo (14-79) follow-up.

Conclusions : In this small case series, DMARD and non-TNFai biologic treatments did not appear to cause IH recurrence, but systemic steroids may delay clearance of histAg. Unlike MTX, HCQ does not increase infection risk, but each patient starting either medicine cleared their IH. Additionally, 50% of patients beginning abatacept cleared their disease to date without their IH worsening. Further study is needed to assess abatacept use in treating cRD with IH.

This study was reviewed and approved by the Nationwide Children’s Hospital Institutional Review Board, approval number IRB17-01241.

## A53 Feasibility of Using Wearable Thermometers to Improve Diagnosis and Treatment of PFAPA

### Jonathan Hausmann^34^, Kalpana Manthiram^148^, Edwin Anderson^33^, Sivia Lapidus^136^, Fatma Dedeoglu^33^, for the CARRA PFAPA Subcommittee

#### ^34^Boston Children's Hospital / Beth Israel Deaconess Medical Center, Boston, MA, USA; ^148^National Institutes of Health, Bethesda, MD, USA; ^33^Boston Children's Hospital, Boston, MA, USA; ^136^Joseph M. Sanzari Children’s Hospital, Hackensack Meridian Health, Hackensack, NJ, USA

##### **Correspondence:** Jonathan Hausmann

Background : PFAPA (periodic fevers, aphthous stomatitis, pharyngitis, and cervical adenitis) is the most common autoinflammatory disease in children. One of the challenges of diagnosing PFAPA is distinguishing fever episodes from those of recurrent viral illnesses. As a result, most children with PFAPA have delays in diagnosis on the order of months to years. In this preliminary study, we seek to assess the feasibility of using wearable thermometers to determine fever patterns in patients with PFAPA.

Methods : Children with PFAPA meeting eligibility criteria for enrollment in the PFAPA Consensus Treatment Plan (CTP) (Table 1) were eligible to enroll in this study. The study was conducted at Boston Children’s Hospital, but consent could be obtained over the phone for participants from any CARRA site, and materials could be sent via mail. Families were given an iThermonitor (Raiing Medical, Boston, MA), an FDA-approved device that records continuous body temperature. Children were asked to wear the iThermonitor for a few days before, during, and after a PFAPA flare. Temperature data was sent automatically and securely to an online dashboard accessible to study investigators. Families also completed an online health survey and fever diary in which they recorded symptoms and timing of medication administration.

Results : Twelve participants (5 boys) were enrolled in the study. Average age at enrollment was 3.9 years (range 2.2-6.7). Nine participants (75%) wore the thermometer and logged temperatures at least once. Two participants had inaccurate temperature recordings and their data were discarded. Participants wore the thermometers continuously during 1-5 episodes (average=2.1) for an average of 10.2 hours per episode (range 0.3-21). Three participants had fever episodes while wearing the device (Figure 1).

Conclusions : In this preliminary study, we were successful in collecting continuous temperature data from children with PFAPA. Participants wore the thermometer for up to 21 hours at a time, and several febrile episodes were captured. However, 25% of participants did not log any temperatures due to technical difficulties in using the app or to loss of interest in the study, and 17% had technical malfunctions with the device. Only 3 participants captured febrile episodes, reflecting the challenges of timing the use of the iThermonitor during fever flares. To address these issues, we plan to call participants regularly to resolve technical issues and to remind them to wear the iThermonitor more regularly. In future studies, we will assess whether continuous temperature monitoring could expedite the diagnosis of PFAPA, facilitate earlier treatment with steroids, and help to provide objective outcomes in comparative effectiveness research studies such as those in the PFAPA CTP.

The IRB at Boston Children’s Hospital approved this study.

Acknowlegements: This study is funded through a CARRA – Arthritis Foundation Grant. The Authors wish to acknowledge the ongoing Arthritis Foundation financial support of CARRA.

**Table 1 (abstract A53).**
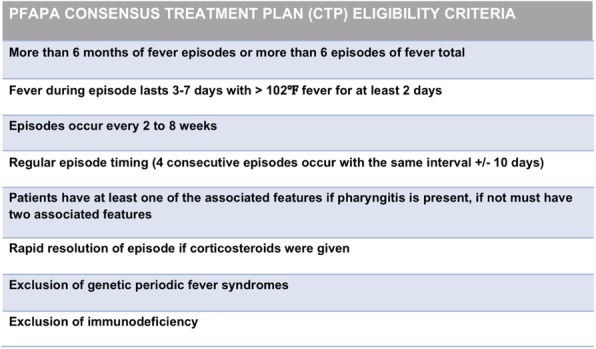
Preliminary eligibility criteria for the PFAPA CTP, which was also used for eligibility into this study

PFAPA Consensus Treatment Plan (CTP) eligibility criteria

**Fig. 1 (abstract A53).**
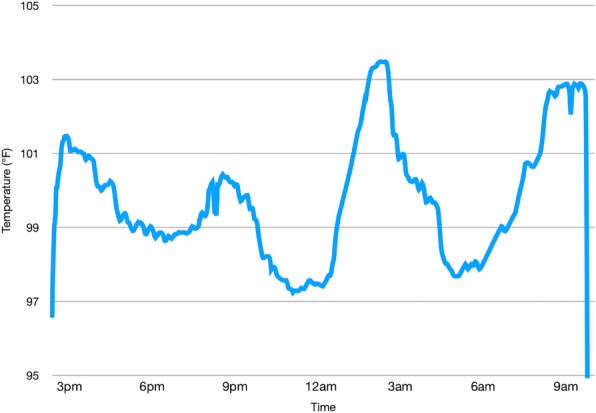
Temperature recordings from participant BCH02 during fever flare. Temperatures were automatically recorded every minute for a total of 1,052 temperatures during the time period shown. Continuous temperature data from participant BCH02

## A54 Pediatric ANCA-Associated Vasculitis (Ped-AAV): Developing Consensus Derived Treatment Protocols (CTPs) For Pragmatic Registry-Based Comparative Evaluation

### Eric Yen^241^, Linda Wagner-Weiner^270^, Vidya Sivaraman^155^, Karen James^294^, Kimberly Morishita^261^, Kathleen O'Neil^278^, David Cabral^261^, for the CARRA ANCA-Associated Vasculitis Workgroup

#### ^241^University of California, Los Angeles, Los Angeles, CA, USA; ^270^University of Chicago, Chicago, IL, USA; ^155^Nationwide Children's Hospital, Columbus, OH, USA; ^294^University of Utah, Salt Lake City, UT, USA; ^261^University of British Columbia and BC Children's Hospital, Vancouver, BC, Canada; ^278^University of Indiana, Indianapolis, IN, USA

##### **Correspondence:** Eric Yen

Background : Because of disease rarity and a lack of randomized controlled trials for Ped-AAV, pediatric specialists have unsystematically adapted AAV treatment strategies based on adult data; this is despite known and potential differences in the effects of the disease and treatments in children versus adults. A 2015 international survey of clinicians caring for Ped-AAV identified wide variation in medication and dosing regimens for treating Ped-AAV and limited utilization of classification criteria and clinical scoring tools. It also endorsed a need for pediatric-specific treatment guidelines. In response, the CARRA AAV workgroup (AAV-WG) has developed consensus treatment protocols (CTPs) to guide treatment, defining a limited range of CTP options to better allow future registry-based comparative effectiveness study.

Methods : From 2015 to 2018, iterative consensus-making for Ped-AAV treatment used nominal group technique in face-to-face meetings with discussion based on existing adult AAV treatment guidelines, a comprehensive literature review (AAV classification, measurement of disease severity/activity/damage, and medical treatment), and surveys of current practice (CARRA and international vasculitis working groups). We aimed for >80% consensus for the 1) target population, 2) remission-induction and remission-maintenance therapy, 3) clinical scoring tools, and 4) outcome measures.

Results : Towards developing Ped-AAV CTPs for comparative study, 25 AAV-WG members found consensus on: 1) Target population: newly diagnosed patients <18 years of age (with granulomatosis with polyangiitis, microscopic polyangiitis, or renal-limited ANCA-associated vasculitis) with severe disease defined by the presence of a major disease item from the Birmingham disease activity score with a pediatric adaptation. 2) The remission-induction alternative protocols used cyclophosphamide (either NIH or EULAR dosing) for 3 to 6 months OR rituximab (choice of 4 doses at 375 mg/m2 or 2 doses at 750 mg/m2, maximum 1 gm/dose) PLUS recommends 0-4 methylprednisolone pulses (30 mg/kg, max 1 gm) for induction, followed by oral corticosteroids starting at 1-2mg/kg/day (max 60 mg/day) with taper goal to 0-10 mg/day (0-0.2 mg/kg/day) by 6-12 months. The remission-maintenance alternative protocols lasting ≥18 months are: methotrexate 0.5 mg/kg/week (max 25 mg), azathioprine 2-3 mg/kg/day (max 200 mg/day), and rituximab Q 6 months (either 2 doses at 375mg/m2, max 500 mg IV or 1 dose at 750 mg/m2, max 1 gm). 3) Proposed clinical scoring tools are Pediatric Vasculitis Activity Score (pVAS) and Pediatric Vasculitis Damage Index (pVDI) to assess disease activity and measure organ damage respectively. 4) Planned outcome measures to assess the efficacy of therapy include standard definitions of activity states: inactive, remission, improvement, refractory, and relapse.

Conclusions : These CARRA Ped-AAV CTPs provide a framework to assess the comparative effectiveness of a limited number of treatment protocols for both remission induction and remission maintenance of Ped-AAV. We will seek broader agreement from the CARRA community with a CARRA-wide survey, to be distributed in early 2019.

The authors wish to acknowledge CARRA, and the ongoing Arthritis Foundation financial support of CARRA.

## A55 Anakinra Usage in Febrile Infection Related Epilepsy Syndrome (FIRES): An International Retrospective Cohort Study

### Eyal Muscal^26^, Elizabeth Wells^62^, Nikita Shukla^26^, Krista Eschbach^86^, Ki Hyeong Lee^6^, Marios Kaliakatsos^158^, Ronny Wickstrom^159^, Maurizio Viri^47^, Elena Freri^84^, Tiziana Granata^84^, Srishti Nangia^160^, Robertino Dilena^304^, Andreas Brunklaus^106^, Mark Wainwright^83^, Mark Gorman^36^, Coral Strendy^36^, Abdurhman Asiri^21^, Asif Doja^90^, Eric Payne^182^, Yi Chen Lai^26^, Sookyong Koh^89^, Jessica Carpenter^62^, Jim Riviello^26^

#### ^26^Baylor College of Medicine/Texas Children's Hospital, Houston, TX, USA; ^62^Children's National Health System, Washington, DC, USA; ^86^Department of Pediatrics, Section of Neurology, Children's Hospital Colorado, University of Colorado, Aurora, CO, USA; ^6^AdventHealth, Child Neurology and Comprehensive Epilepsy Center, Orlando, FL, USA; ^158^Neurology Department, Great Ormond Street Hospital, London, England; ^159^Neuropediatric Unit, Department of Women's and Children's Health, Karolinska University Hospital, Solna, Sweden; ^47^Childhood Neuropsychiatric Departement, University Hospital Maggiore della Carità, Novara, Italy; ^84^Department of Pediatric Neuroscience, Fondazione IRCCS Instituto Neurological “Carlo Besta” Milan, Italy; ^160^New York Presbyterian Hospital-Weill Cornell Medical College, New York, NY, USA; ^304^UOC Neurofisiopatologia - Fondazione IRCCS Ca' Granda Ospedale Maggiore Policlinico, Milan, Italy; ^106^Fraser of Allander Neurosciences Unit, Royal Hospital for Children, Glasgow, Scotland; ^83^Department of Neurology, University of Washington, Seattle, WA, USA; ^36^Boston Children's Hospital, Harvard Medical School, Boston, MA, USA; ^21^Aseer Central Hospital, Abha, Saudi Arabia; ^90^Division of Neurology, CHEO Research Institute, Faculty of Medicine, University of Ottawa, Ottawa, ON, Canada; ^182^Pediatric Neurology, Mayo Clinic, Rochester, MN, USA; ^89^Division of Neurology, Department of Pediatrics, Emory University School of Medicine, Atlanta, GA, USA

##### **Correspondence:** Eyal Muscal

Background : Febrile Infection Related Epilepsy Syndrome (FIRES) is a catastrophic epilepsy syndrome that develops in children after an innocuous febrile illness. It is hypothesized that FIRES may be an innate immune condition characterized by an amplified IL-1 response. We report preliminary safety and outcomes data from the largest international cohort of children who have received anakinra for FIRES.

Methods : Case requests were sent to the McMaster Rheumatology list-serve during the period of 10/2017-2/2018. Participants at a FIRES symposium at the American Epilepsy Society meeting in 12/2017 reached out to collogues for additional cases. We collected clinical manifestations, anakinra usage parameters, and functional outcomes data via a standardized abstraction tool. We analyzed changes in continuous variables utilizing paired t tests and assessed associations between clinical parameters using Pearson correlation coefficients.

Results : We abstracted data on 25 children who received anakinra during their initial FIRES illness. Cases were obtained from 13 centers in 6 countries (US and Europe predominant). Age at diagnosis was 8.4 + 3.4 (range 4-16 yrs.). Boys predominated (68%) in a multi-ethnic cohort (Caucasian 32%, Hispanic 16%, E. Asian 16%). CRP/ESR elevations were seen in 65% and CSF protein elevation only in 30% of children. Testing was negative for autoimmune encephalitis antibodies in all patients. CSF neopterin was obtained in 5 children (60% elevated). All 9 children with serum cytokine testing had elevations (IL-6, IL-8 prevalent). 76% of children required pentobarbital and burst suppression in addition to > 4 anti-seizure medications. Prior to anakinra, immunomodulators were started but did not dampen seizure burden (corticosteroids/IVIG 88% and rituximab 20%). The ketogenic diet was started in 76% of children and epidiolex or CBD started in 32%. Anakinra was started 20.6 + 11.5 days after illness. Mean maximum anakinra dose was 6.3 + 3.1 mg/kg. Length of anakinra therapy was 116.6 + 134.6 days. Seizure number and duration were reduced 1 week after starting anakinra (not statistically significant with missing paired data). An increase in the number of infections on anakinra was not significant (4.0 + 2.6 vs 1.5 + 5.0). Only one child had anakinra stopped due to infection. Other Adverse events includes DRESS in 12% and cytopenia in 8%. ICU and hospital LOS were protracted (respectively 57.9 + 33.8, 85.3 + 63.3 days) and 12% of the patients passed away. At f/u all children had epilepsy (4.2 + 7.9 seizures/ wk.) 35% had no or mild disability and another 35% moderate disability. Cognitive deficits were most prominent in attention and executive domains. 57% of children returned to school, most with academic accommodations. Earlier anakinra correlated with reduced ICU and hospital LOS [r=0.50 (p=0.01) and r=0.48 (p=0.03)] and reduced number of seizures/week at last f/u [r=0.55 (p=0.04)].

Conclusions : Anakinra usage was well tolerated in a cohort of critically ill children with FIRES. Cognitive outcomes of children in this cohort were more robust than older cohorts. Earlier anakinra usage correlated with reduced disease severity. Multi-disciplinary efforts are underway to design prospective FIRES anakinra trials.

IRB/ethics approvals were obtained at all institutions.

## A56 Identifying and discovering genetic variants for monogenic lupus

### Linda Hiraki^195^, Sergey Naumenko^224^, Jingjing Cao^107^, Declan Webber^195^, Daniela Dominguez^195^, Bhooma Thiruvahindrapuram^223^, Deborah Levy^195^, Andrew Paterson^107^, Earl Silverman^195^

#### ^195^Rheumatology, SickKids, Toronto, ON, Canada; ^224^The Centre for Computational Medicine, Research Institute, Toronto, ON, Canada; ^107^Genetics & Genome Biology, Research Institute, SickKids, Toronto, ON, Canada; ^223^The Centre for Applied Genomics, SickKids, Toronto, ON, Canada

##### **Correspondence:** Linda Hiraki

Background : There is strong evidence that genetics plays an important role in the pathogenesis of systemic lupus erythematosus (SLE). Within the broad category of SLE, there are genetically distinct Mendelian diseases presenting with lupus features, also known as monogenic lupus. This theory has been proven by the discovery of variants in single genes causing monogenic SLE in small numbers of individuals with young-onset disease and/or families with multiple members diagnosed with SLE. Specific Aims: To assemble a North American cohort and bio-repository comprised of families with monogenic lupus. Next generation sequencing (NGS) including whole exome sequencing (WES) and whole genome sequencing (WGS) of this population will identify variants and genes associated with disease risk.

Methods : 1. WES (Illumina HiSeq 2500 platform) n=17 patients with both cSLE and macrophage activation syndrome (MAS). 2. WGS (Illumina HiSeq X platform) n=8 cSLE patients diagnosed < 10 years and/or evidence of consanguinity. Variant calling completed with GATK and HAS, and functional annotation with ANNOVAR at The Centre for Applied Genomics, SickKids (annotation pipeline, v26.2, v.26.5). Small variants recalled and reannonated with gatk4/bcbio/VEP/vcfanno gemini. We prioritized variants based on population frequency in GNOMAD, functional impact (HIGH or MED: coding stop gain or lost, missense, frameshift, splice site and splice region) and panels of MAS-specific genes (AP3B1, BLOC1S6, CD27, GATA2, ITK, LYST, NLRC4, PRF1, RAB27A, SH2D1A, SIRPA, SLC7A7, STX11, STXBP2, UNC13D, XIAP) and lupus genes (ACP5, ADAR, C1QA, C1QB, C1QC, C1R, C1S, C2, C3, C4A, C4B, CYBB, DNASE1, DNASE1L3, FASLG, IFIH1, KRAS, MAN2B1, NEIL3, PEPD, PRKCD, PSMA3, PSMB4, PSMB8, PTEN, PTPN11, RAG2, RNASEH2A, RNASEH2B, RNASEH2C, SAMHD1, SHOC2, SLC7A7, TMEM173, TNFRSF6B, TREX1).

Results : We discovered 5 rare (MAF < 1%) variants in 3/17 MAS samples in 5 genes (SLC7A7; ITK, LYST, SIRPA - 3 variants in 1 sample; BLOCK1S6 ). When relaxing MAF threshold to MAF < 5% we discovered additional 8 variants in 7/17 MAS samples in 6 genes (SLC7A7, AP3B1, PRF1, UNC13D, LYST, STXBP2). In total, we discovered 13 rare (MAF < 5%) variants in 8/17 MAS samples. 4/13 variants were in splice regions and 9/13 were missense variants. Among SLE WGS samples we discovered rare (MAF < 1%) variants in 5/8 samples in genes (PKRCD, ADAR, DNASE1, SLC7A7, C4A, C2). One samples carried variants in two genes (stop gain in DNASE1 and missense in SLC7A7) In addition, we filtered many rare non-coding variants in target genes, however, their functional interpretation is not yet established.

Conclusions : NGS has identified candidate variants leading to MAS in SLE, as well as monogenic lupus. These findings provide insights into the pathogenesis of SLE as well as being potential prognostic factors and therapeutic targets.

Local IRB obtained.

Acknowlegements: This project was funded by a CARRA-Arthritis Foundation grant. The authors wish to acknowledge the ongoing Arthritis Foundation financial support of CARRA

## A57 Skin-biopsy Evidence of Decreased Epidermal Neurite Density in Juvenile Fibromyalgia

### Alexis Boneparth^85^, Shan Chen^82^, Daniel Horton^203^, Lakshmi Moorthy^202^, Heather Downs^81^, Hang Lee^29^, Anne Louise Oaklander^81^

#### ^85^Department of Pediatrics, Columbia University Medical Center, New York, NY, USA; ^82^Department of Neurology, Rutgers Robert Wood Johnson Medical School, New Brunswick, NJ, USA; ^203^Rutgers University, New Brunswick, NJ, USA; ^202^Rutgers Robert Wood Johnson Medical School, New Brunswick, NJ, USA; ^81^Department of Neurology, Massachusetts General Hospital and Harvard Medical School, Boston, MA, USA; ^29^Biostatistics Center, Massachusetts General Hospital, Boston, MA, USA

##### **Correspondence:** Alexis Boneparth

Background : Fibromyalgia (FM) is defined by the presence of idiopathic, chronic, widespread musculoskeletal pain. Approximately 40% of adults with FM have lower-leg skin biopsies with decreased epidermal neurite densities (END). Decreased END is a known diagnostic finding in small-fiber polyneuropathy (SFN). However, the clinical significance of decreased END in patients with FM is unknown. Similar END findings in SFN and FM suggest the possibility of a shared pathophysiology; alternatively, END findings in FM may have a distinct physiologic explanation. The prevalence and clinical significance of decreased END in juvenile fibromyalgia (JFM) are unknown. The aim of this study is to test whether JFM is associated with decreased END.

Methods : We screened 20 participants aged 13-20 years with JFM diagnosed by pediatric rheumatologists. 17 met modified American College of Rheumatology criteria for JFM and completed cross-sectional surveys assessing pain severity, functional disability, and autonomic symptom severity. All 17 were offered standard lower-leg skin biopsy for assessment of END and 12 families consented. Biopsies were removed from anesthetized lower-leg skin, processed, immunolabeled against PGP9.5 and analyzed using standard methods in a Joint Commission-accredited clinical diagnostic lab. The primary outcome was END <5th centile of age/gender/race predicted normal distribution. Control skin biopsies were blindly selected from a cohort of 351 healthy normal volunteers whose skin biopsy data was used to establish the lab's normative values for END. An algorithm selected all who matched the JFM participants by age, sex and race, which yielded 25 controls. We compared END between groups using Wilcoxon rank-sum test and Fisher’s exact test.

Results : There were no significant differences between participants with JFM and controls for the 3 salient demographic variables (mean ages: JFM, 16.9 years; controls 17.9 years [p=0.17]). More participants with JFM than controls had low END below the 5% centile (67% vs. 4%, p<0.001). Participants with JFM averaged 239±147 epidermal neurites/mm2 skin surface area vs. 403±152/mm2 in healthy controls (p=0.003).

Conclusions : These results demonstrate high prevalence of abnormally low END in youth with JFM, in concordance with evidence of low END in adults with fibromyalgia. Further study is needed to confirm these findings in larger pediatric populations and to elucidate their clinical significance.

Participation of human subjects was approved by the Institutional Review Boards of Columbia University Medical Center, Rutgers Robert Wood Johnson Medical School, and Massachusetts General Hospital.

Acknowledgments: The authors wish to acknowledge the ongoing Arthritis Foundation financial support of CARRA

This study is supported by the CARRA-Arthritis Foundation Grant Program.

Supported in part by the National Institutes of Health (R01NS093653, K24NS059892) and the

Department of Defense (GW093049) (to ALO) as well as the Harvard Catalyst | The Harvard Clinical

and Translational Science Center (National Center for Advancing Translational Sciences, National

Institutes of Health Award UL 1TR002541) and financial contributions from Harvard University and

its affiliated academic healthcare centers.

## A58 Implementation of Six Core Elements of Health Care Transition in a Pediatric Rheumatology Practice

### Stacy Ardoin^155^, Rebecca Furru^155^, Darby MacDonald^155^, Vidya Sivaraman^155^, Paul Jensen^131^, Stephanie Lemle^155^

#### ^155^Nationwide Children's Hospital, Columbus, OH, USA; ^131^Intermountain Healthcare, Salt Lake City, UT, USA

##### **Correspondence:** Stacy Ardoin

Background : Transition from pediatric to adult health care remains challenging with successful transfers of care occurring at best 50% of the time. A federally funded initiative, Got Transition, has developed and successfully implemented the Six Core Elements of Health Care Transition. This approach is applicable to all primary care and pediatric subspecialty patients and includes the following components: transition policy, transition tracking and monitoring, transition readiness, transition planning, transfer of care and transfer completion. We performed a quality improvement (QI) intervention to implement these elements in a large, single center pediatric rheumatology practice.

Methods : We established a transition-focused QI team including pediatric rheumatology physician, nurse practitioner and fellow providers, adult rheumatology providers, social worker, nurse, and QI staff with input from patients and families. This team met monthly and developed a key driver diagram, using information gathered from a needs assessment of adult and pediatric providers. The team developed a health literacy appropriate transition policy and distribution plan, initiated nurse practitioner and social worker led protocol to assess transition readiness, developed a registry to monitor transition and transfer processes, and developed a standardized transfer of care bundle to be provided to patient and adult provider. In addition, the team developed an annual transfer satisfaction survey of adult rheumatology colleagues. P-charts were created to document outcomes over times and standard methods were used to identify special cause changes.

Results : The most successful interventions have included dissemination of transition policy to patients (Figure 1), development of transition registry and documentation of transfer completion to adult rheumatologist (Figure 2). To date, 87 individuals have completed transfer of care in our registry and 63 are within 12 months of anticipated transfer. Additional QI interventions have included offering nurse practitioner led self-management visits, creation of a paper transition resource packet, development of an adult rheumatology provider directory, use of a standard transfer to adult care packet which includes letter and medical summary.

Conclusions : A QI approach to implementing the Six Core Elements of Health Care Transition in a large pediatric rheumatology practice was feasible and successful. Our most successful interventions included implementing a procedure to disseminate a transition policy to all patients 14 years of age or older and to sustain this improvement for more than 12 months. Additionally, we increased our documentation of transfer completion to adult care by developing a transition registry and using a standardized transfer packet to provide information to patients and adult providers. One challenge remains universal implementation of the transition readiness assessment. Next steps include efforts to perform annual transition readiness assessment on all patients 14 years and older and developing a standardized approach to documenting transition milestones in the electronic medical record.

This QI study was deemed by IRB to be exempt from IRB review.

**Fig. 1 (abstract A58).**
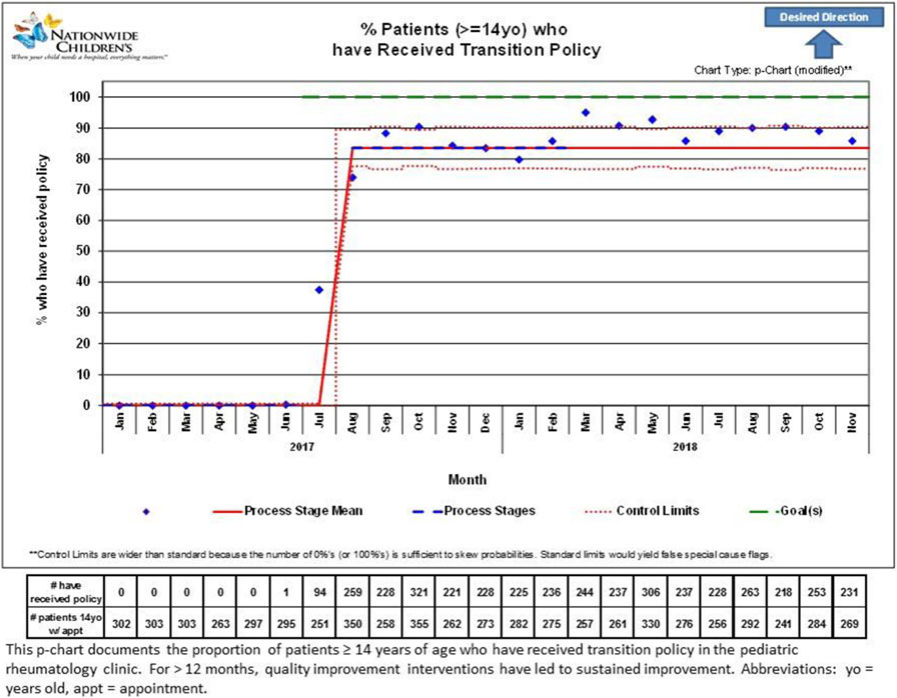
Transition Policy Dissemination. This p-chart documents the proportion of patients ≥ 14 years of age who have received transition policy in the pediatric rheumatology clinic. For > 12 months, quality improvement interventions have led to sustained improvement. Abbreviations: yo = years old, appt = appointment

**Fig. 2 (abstract A58).**
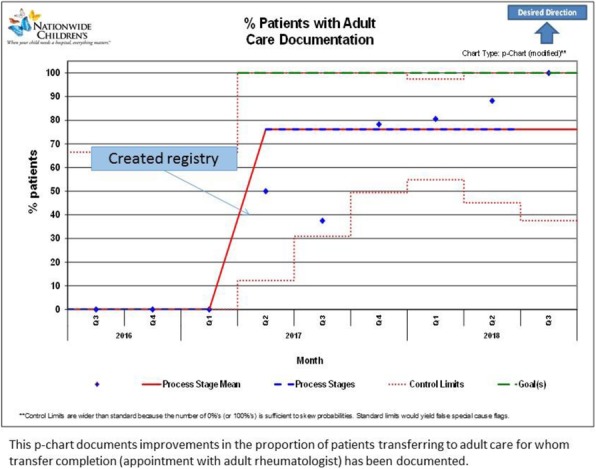
Documentation of Transfer of Care to Adult Provider. This p-chart documents improvements in the proportion of patients transferring to adult care for whom transfer completion (completed appointment with adult rheumatologist) has been documented

## A59 Demographic and Clinical Characteristics of Patients with Systemic Lupus Erythematosus and Related Conditions in the CARRA Registry: An Update

### Mary Beth Son^33^, Anne Dennos^95^, Stacy Ardoin^155^, Deborah Levy^195^, Andrea Knight^229^, Tamar Rubinstein^9^, Scott Wenderfer^23^, Laura Lewandowski^161^, Lisa Arkin^302^, Kaveh Ardalan^172^, Barry Myones^166^, Aimee Hersh^294^, for the CARRA Registry Investigators

#### ^33^Boston Children's Hospital, Boston, MA, USA; ^95^Duke University, Durham, NC, USA; ^155^Nationwide Children's Hospital, Columbus, OH, USA; ^195^Rheumatology, SickKids, Toronto, ON, Canada; ^229^The Hospital for Sick Children, Toronto, ON, Canada; ^9^Albert Einstein College Of Medicine, Children's Hospital At Montefiore, Bronx, NY, USA; ^23^Baylor College of Medicine, Houston, TX, USA; ^161^NIH, Bethesda, MD, USA; ^302^University of Wisconsin School of Medicine and Public Health, Madison, WI, USA; ^172^Nothwestern University Feinberg School of Medicine, Chicago, IL, USA; ^166^Pediatric Rheumatology, Sugar Land, TX, USA; ^294^University of Utah, Salt Lake City, UT, USA

##### **Correspondence:** Mary Beth Son

Background : We sought to describe the demographic and clinical characteristics at baseline (BL) and most recent follow up (FU) for patients (pts) enrolled in the CARRA Registry with SLE and related conditions.

Methods : Patients were eligible for enrollment in the CARRA registry if they were diagnosed with SLE per the Systemic Lupus International Collaborating Clinic (SLICC) Classification Criteria or developed new onset lupus nephritis within 2 years of BL visit, or if they had a related condition: cutaneous Lupus Erythematosus (CLE), Sjogren’s (SS), Mixed Connective Tissue Disease (MCTD), Anti-Phospholipid Syndrome (APS) or probable SLE (≤3 SLICC criteria). IRB approval was via Protocol 00054616 (Duke University).

Results : From March 2017-December 2018, 380 pts from 46 CARRA sites have enrolled with 330 SLE pts, 6 CLE pts, 15 SS pts, 22 MCTD pts, and 7 probable SLE pts. 219 pts have ≥1 FU with a median of 8.9 months from BL to most recent FU (IQR: 5.3, 12.2; range 3-19). The median age at enrollment was 15.7 years (IQR: 13.4, 17.1) and the median age at diagnosis was 14.3 years (IQR: 11.8,16). 315 pts (83%) are female. Subject ethnicity/race is 26% Black, 26% White, 22% Hispanic, 12% Asian, 9% with > 1 race declared and 5% other/unknown. Over half of pts (198, 52%) have private insurance, 32% (n=122) have Medicaid. 94% of pts were ANA positive. At BL, the mean Systemic Lupus Erythematosus Disease Activity Index (SLEDAI) score was 5.2 (SD 6.2), median = 2 (range 0-37). The median SLICC Damage Index score was 0.0 (range 0-8). 61% of pts were prescribed oral prednisone at BL with a median dose of 20 mg (IQR 10, 30) whereas 45% were prescribed prednisone at most recent FU (median dose 10 mg, IQR: 5, 19.9). Medication data collected at BL are listed in Table 1. One third of pts (n=126) were being treated for lupus nephritis at the time of BL, with 50 pts (40%) according to a CARRA Lupus Nephritis Consensus Treatment Plan. Of patients in the CTPs, 75% received mycophenolate/mycopholic acid and 24% received intravenous cyclophosphamide for induction treatment. Steroid regimens included primary oral (66%), oral and intravenous (24%) and primarily intravenous (10%). Manifestations of SLE at BL and FU were varied (Table 2) but serologic disease, mucocutaneous disease and active nephritis were the most prevalent.

Conclusions : Almost 400 SLE pts are enrolled in the CARRA Registry. This is a multi-racial cohort with moderate disease activity and varied treatment. Further enrollment will continue to build a robust data source to study disease course and outcomes in a pediatric SLE cohort.

Acknowledgements: This work could not have been accomplished without the aid of the following organizations: The NIH’s National Institute of Arthritis and Musculoskeletal and Skin Diseases (NIAMS) & the Arthritis Foundation. We would also like to thank all participants and hospital sites that recruited patients for the CARRA Registry.

**Table 1 (abstract A59).** Medication Use Documented at Baseline Visit of Patients with SLE and Related Conditions in the CARRA Registry (n=380)

Medication	n (%)
Hydroxychloroquine	336 (88%)
Oral Prednisone	232 (61%)
Mycophenolate mofetil/Mycophenolic Acid	161 (42%)
Methotrexate	63 (17%)
Cyclophosphamide	49 (13%)
Azathioprine	50 (13%)
Rituximab	47 (12%)
Belimumab	4 (1%)

**Table 2 (abstract A59).** Components of SLEDAI Present within 30 days of Baseline Visit and at Most Recent Follow Up Visits

SLEDAI Component	Baseline Visit, n=380 n (%)	Most Recent Follow Up Visit , n=219 n (%)
Seizure	0	0
Psychosis	4 (1%)	0
Organic Brain Syndrome	0	0
Visual Disturbance	4 (1%)	2 (1%)
Cranial Nerve Disorder	0	0
Lupus Headache	11 (3%)	4 (2%)
Cerebrovascular Accident	0	0
Vasculitis	11 (3%)	7 (3%)
Arthritis	61 (16%)	46 (12%)
Myositis	15 (4%)	7 (3%)
Urinary Casts	57 (15%)	18 (8%)
Hematuria	27 (7%)	11 (5%)
Proteinuria	27 (7%)	7 (3%)
Pyuria	23 (6%)	7 (3%)
Rash	80 (21%)	33 (15%)
Alopecia	27 (7%)	7 (3%)
Mucosal Ulcers	30 (8%)	7 (3%)
Pleurisy	4 (1%)	0
Pericarditis	4 (1%)	0
Low Complement	148 (39%)	68 (31%)
Increased DNA Binding	103 (27%)	50 (23%)
Fever	27 (7%)	2 (1%)
Thrombocytopenia	15 (4%)	2 (1%)
Leukopenia	53 (14%)	22 (10%)

IRB approval was via Protocol 00054616 (Duke University)

## A60 Research Priorities for Addressing Mental Health Needs of Pediatric Patients with Rheumatologic Disease

### Tamar Rubinstein^9^, Ekemini Ogbu^101^, Lindsay Waqar^226^, Jennifer Woo^266^, William Lapin^24^, Lawrence Ng^293^, Erin Treemarcki^311^, Andrea Knight^229^, for the CARRA Mental Health Workgroup

#### ^9^Albert Einstein College Of Medicine, Children's Hospital At Montefiore, Bronx, NY, USA; ^101^Emory University and Children's Healthcare of Atlanta, Atlanta, GA, USA; ^226^The Children’s Hospital of Philadelphia, Philadelphia, PA, USA; ^266^University of California Los Angeles, Los Angeles, CA, USA; ^24^Baylor College of Medicine, Houston, TX, USA; ^293^University of Toronto, Toronto, ON, Canada; ^311^Weil Cornell Medical College, New York, NY, USA; ^229^The Hospital for Sick Children, Toronto, ON, Canada

##### **Correspondence:** Tamar Rubinstein

Background : Mental health problems are prevalent in pediatric rheumatology patients. Gaps in knowledge exist regarding the detection, effective treatment, and the impact of mental illness on patients with rheumatologic disease. To address these gaps and direct research efforts to improve mental health for children and adolescents with rheumatologic diseases, the Childhood Arthritis and Rheumatology Research Alliance (CARRA) Mental Health Workgroup developed and prioritized an agenda of research topics.

Methods : A systematic review of the literature on mental health in pediatric rheumatology was completed and published. From the review, 5 major research domains in further need of study were identified: (A) mental health burden and relationship to pediatric rheumatologic disease, (B) impact of mental health disorders on outcomes, (C) mental health awareness and education, (D) mental health screening, and (E) mental health treatment. Research topics within these areas were developed and presented at the CARRA Mental Health Workgroup Annual Meeting in April 2018, where they were discussed and refined. An online survey was created to prioritize the 33 refined topics. The survey was emailed to 103 people who expressed interest in the workgroup, including pediatric rheumatologists, patients/parents, research coordinators, psychologists, social workers, and members of industry. Participants were asked to score each research topic on a 5-point Likert scale on how important, feasible, and actionable they were. Likert scale responses were 1 = Low, 2 = Somewhat Low, 3 = Neutral, 4 = Somewhat High, 5 = High. We defined the cut-off for “highly important,” “highly actionable,” and “highly feasible” as a ≥4 mean score. Participants were also asked to provide an overall ranking of the research topics within each research area.

Results : Seventy-three participants (71%) responded to the survey, and 59 of the respondents completed the survey (81%), 6 participants (8%) opted out and 8 participants (11%) started by did not complete the survey. Among the proposed research topics 32/33 were rated as highly important, 8/33 were rated as highly actionable (Table 1), and 4/33 were rated as highly feasible (Table 2). Topics related to the impact of mental health on clinical outcomes were rated most important, and those related to mental health screening were rated most feasible and actionable (Table 1). Overall rankings revealed additional topics of high priority within each research domain (Figure 1).

Conclusions : Addressing gaps in knowledge about how mental health affects youth with rheumatologic disease, and how best to address mental health for these patients is an important step in improving care. We have identified high priority research topics regarding mental health of pediatric rheumatology patients in need of further investigation that are feasible to study, and believed to lead to actionable results in patient care.

Acknowledgements: This project was funded by a CARRA – Arthritis Foundation Grant. The authors wish to acknowledge CARRA, and the ongoing Arthritis Foundation financial support of CARRA.

This study was approved by The Children’s Hospital of Philadelphia Institutional Review Board (IRB 18-015073).

**Table 1 (abstract A60).** Highest Scored Topics for Mental Health Research in Pediatric Rheumatology by Actionability

Actionable Rank	Research Topic	Mean Score (±SD)
1	(D1) Determine which mental health conditions are most important to screen for in pediatric patients with rheumatologic disease.	4.3 (±0.8)
2	(D2) Determine the accuracy of mental health screening tools for identifying mental health conditions in specific pediatric rheumatology disease populations (i.e. validation of tools for disease-specific diagnostic cut-points).	4.2 (±0.8)
3	(B6) Investigate the impact of mental health on quality of life and social outcomes for patients.	4.1 (±0.8)
4	(D4) Determine barriers and facilitators to mental health screening in the pediatric rheumatology setting.	4.1 (±0.8)
5	(D5) Determine acceptability of mental health screening in pediatric rheumatology clinics for patients, caregivers, clinicians, and identify strategies to improve acceptability.	4.1 (±0.8)
6	(A1) Determine the prevalence and incidence of mental health disorders in pediatric patients with rheumatologic disease, as well as socio-demographic and disease-specific risk factors.	4.0 (±0.8)
7	(E6) Investigate factors contributing to social-cultural disparities in mental health care for pediatric patient with rheumatology disease, and test interventions to reduce disparities.	4.0 (±0.8)
8	(B1) Investigate the impact of mental health on clinical outcomes, such as disease activity.	4.0 (±0.8)

Means and standard deviations presented of the highest ranking research topics from 33 developed research topics addressing mental health in pediatric rheumatology around actionability (ability to apply the results of research on the topic towards advancing clinical care and pediatric rheumatology research)

**Table 2 (abstract A60).** Highest Scored Topics for Mental Health Research in Pediatric Rheumatology by Feasibility

Feasibility Rank	Research Topic	Mean Score (±SD)
1	(D4) Determine barriers and facilitators to mental health screening for pediatric patients with rheumatologic disease.	4.3 (±0.8)
2	(D2) Determine the accuracy of mental health screening tools for identifying mental health conditions in specific rheumatology disease populations.	4.2 (±0.8)
3	(A1) Determine the prevalence and incidence of mental health disorders in pediatric patients with rheumatologic disease, as well as socio-demographic and disease-specific risk factors.	4.1 (±0.8)
4	(D1) Determine which mental health conditions are most important to screen for in pediatric patients with rheumatologic disease.	4.1 (±0.8)

Means and standard deviations presented of the highest ranking research topics from 33 developed research topics addressing mental health in pediatric rheumatology around feasibility (ease with which research related to the topic can be conducted and completed)

**Fig. 1 (abstract A60).**
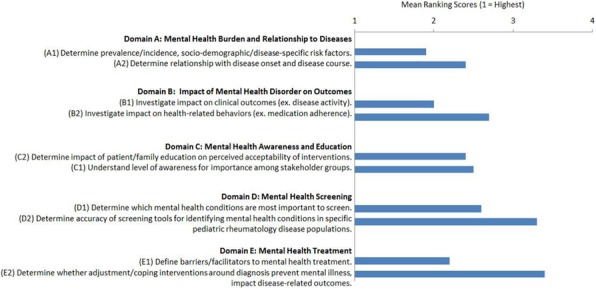
Highest Ranking Research Topics For Mental Health Research in Pediatric Rheumatology by Research Domains. Highest ranked mental health research topics among 33 research topics by domain

## A61 Changes in Healthcare Transition Views, Practices, and Barriers among CARRA Pediatric Rheumatology Providers from 2010 to 2018

### Erica Lawson^267^, Kiana Johnson^97^, Cuoghi Edens^239^, Peter Chira^243^, Aimee Hersh^294^, Y. Ingrid Goh^229^, Joyce Hui-Yuen^68^, Rebecca Sadun^92^, Nora Singer^42^, Lynn Spiegel^240^, Jennifer Stinson^240^, Patience White^109^, for the CARRA Transition Subcommittee

#### ^97^East Tennessee State University, Johnson City, TN, USA; ^239^University of Chicago, Chicago, IL, USA; ^243^University of California San Diego, CA, USA; ^294^University of Utah, Salt Lake City, UT, USA; ^229^The Hospital for Sick Children, Toronto, ON, Canada; ^68^Cohen Children's Medical Center, New Hyde Park, NY, USA; ^92^Duke University, Durham, NC, USA; ^42^Case Western Reserve University, Cleveland, OH, USA; ^240^University of Toronto (SickKids), Toronto, ON, Canada; ^109^George Washington University, Washington, DC, USA; ^267^University of California San Francisco, San Francisco, CA, USA

##### **Correspondence:** Erica Lawson

Background : Healthcare transition is defined as the planned process of moving from a child to an adult model of health care, with or without a transfer to a new clinician. In 2016 the American College of Physicians partnered with national organizations, including the American College of Rheumatology (ACR), to develop tools to promote a smooth transition to adult-oriented care. We aim to assess current transition practices and beliefs among Childhood Arthritis and Rheumatology Research Alliance (CARRA) rheumatology providers, and to identify differences from a 2010 provider survey published by Chira et al.

Methods : In April 2018, CARRA members received a 25-item online survey about healthcare transition. Got Transition’s Current Assessment of Health Care Transition Activities for Transitioning Youth to Adult Health Care Providers was used to measure clinical transition processes on a scale of 1 (basic) to 4 (comprehensive). Bivariate data analysis was used to compare 2010 and 2018 survey findings.

Results : Over half of CARRA members completed the 2018 survey (217/396). Participants included pediatric rheumatologists (74%), adult- and pediatric-trained rheumatologists (4%), pediatric rheumatology fellows (18%), and other (4%), including emeritus faculty and mid-level providers. Most belonged to university-affiliated practices (87%) in the U.S. (91%). Most providers aim to transfer patients at age 18 (23%) or 21 (33%), but the actual age of transfer is often 21 or older (56%). The most common target age to begin transition planning was 15-17 (49%). Few providers use the ACR transition tools (31%) or have a dedicated transition clinic (23%). Only 17% have a transition policy in place; 63% do not consistently address healthcare transition. Transition outcomes of interest included an adult rheumatology visit within 6 months of the last pediatric visit (80%), adherence to medications and plan of care (78%), continuous insurance coverage (78%), and patient-reported gaps in access to care (76%). When compared to the 2010 survey, improvement was noted in 3 of 12 transition barriers: availability of adult primary care providers, availability of adult rheumatologists, and transition knowledge and skills of pediatric staff (p<0.001). However, more providers cited the close bond among adolescents, parents and pediatric providers as a barrier (Figure 1).

Conclusions : This survey of pediatric rheumatology providers demonstrates some improvement in transition barriers since 2010, though most practices still provide minimal support for patients and providers around healthcare transition. Further research is needed to understand how to effectively facilitate transition to adult care for young adults with childhood-onset rheumatic diseases, as well as quality improvement processes that can create a standardized approach to transition support.

An exemption was approved for this study by the Institutional Review Boards of the University of Utah.

Acknowledgements: This project is funded by a CARRA – Arthritis Foundation grant. The authors wish to acknowledge CARRA, and the ongoing Arthritis Foundation financial support of CARRA.

**Fig. 1 (abstract A61).**
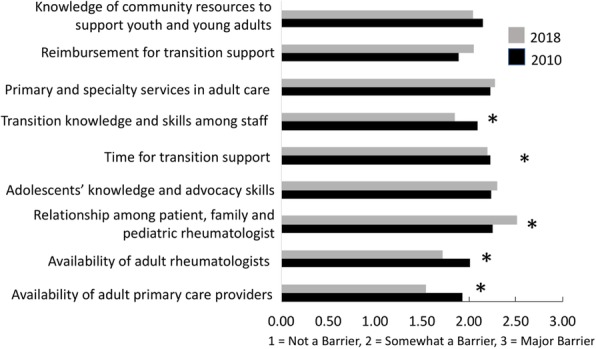
Barriers to Healthcare Transition Reported by Pediatric Rheumatology Providers, 2010 and 2018

## A62 Ultrasound Evaluation of Oligoarticular Juvenile Idiopathic Arthritis Patients Pre and Post-Treatment: A Pilot Study

### Leandra Woolnough^276^, Heather Benham^221^

#### ^276^University of Florida, Gainesville, FL, USA; ^221^Texas Scottish Rite Hospital for Children, Dallas, TX, USA

##### **Correspondence:** Leandra Woolnough

Background : Musculoskeletal ultrasound (US) is a non-invasive technique that detects joint inflammation. Inflammation detected by ultrasound has been shown to predict disease course in adults with rheumatoid arthritis. Currently, there is no unified approach for identifying early or subclinical joint inflammation in Oligoarticular Juvenile Idiopathic Arthritis (oJIA).

Methods : Six newly diagnosed oJIA patients with clinically active arthritis were included in the study were evaluated at new diagnosis visit then at a follow up visit. Subjects underwent US of bilateral wrists (mid-carpal and radiocarpal recesses) and bilateral knees (suprapatellar, medial and lateral recesses) by grey-scale and power Doppler at baseline then 3.4 months later. US scans were read by three independent pediatric radiologists blinded to the clinical musculoskeletal exam. Joint pathology was described by depth (mm) and semi-quantitative scale (range 0-3).

Results : Median clinical disease activity scores decreased from 3.0 (range 2-6) to 1.25 (range 0- 2). Median total lesion measurements in clinically active joints decreased from 6.3mm (range 2.5-14) to 2.8mm (range 1-12.2), synovial thickening score decreased slightly from a median of 1.8 (range 1.3-3) to 1.0 (0.67-2.0), joint effusion score decreased from median of 1.0 (range 0-3) to 0.3 (range 0-2) and hyperemia score decreased from 0.8 (range 0.3-1.7) to 0.3 (range 0-0.3) following treatment. Inter-rater reliability was excellent for the three knee recesses and fair for the two wrist recesses with the exception of being poor for the right radiocarpal recess.

Conclusions : This study demonstrated the feasibility of utilizing US to evaluate joints of newly diagnosed patients with oJIA. While lesions with clinically apparent correlates in a large joint such as the knee were identified with good reliability, subclinical findings of questionable clinical significance in the complex joint of the wrist were described with greater variation.

This study was approved by the Texas Scottish Rite Research Advisory Panel and the UT Southwestern Institutional Review Board.

Acknowledgements: This project was funded by a CARRA-Arthritis Foundation grant. The authors wish to acknowledge the ongoing Arthritis Foundation financial support of CARRA.

## A63 Von Willebrand Factor is Localized in the Extravascular Tissue of Patients with Juvenile Scleroderma

### Natalia Vasquez Canizares^9^, Beamon Agarwal^108^, Dawn Wahezi^9^, Tamar Rubinstein^9^, Morayma Reyes Gil^10^

#### ^9^Albert Einstein College of Medicine, Children's Hospital At Montefiore, Bronx, NY, USA; ^108^GenomeRxUS LLC, Secane, PA, USA; ^10^Albert Einstein College of Medicine, Montefiore Medical Center, Bronx, NY, USA

##### **Correspondence:** Natalia Vasquez Canizares

Background : Von Willebrand Factor (vWF) is a glycoprotein synthesized in endothelial cells and megakaryocytes that has an essential role in primary hemostasis. There is increasing evidence indicating that vWF is involved in inflammation and may play a role in the pathogenesis of cutaneous inflammatory conditions. Our objective was to study the role of vWF in the pathogenesis of skin manifestations of patients with Juvenile Scleroderma (JScl) and Juvenile Dermatomyositis (JDM).

Methods : We examined 8 skin biopsies from 2 patients with systemic sclerosis (SSc), 2 with localized scleroderma (LS) and 4 with JDM. Double immunofluorescence staining was performed in each tissue with antibodies against vWF and collagens type I and III. DAPI (4′, 6-diamidino-2-phenylindole) was also used for counterstaining of inflammatory cells. Tissue staining patterns were compared between groups.

Results : Biopsies were obtained from the upper extremity of 7 females and the lower extremity of 1 male. Median age and disease duration from the first presenting symptom at time of biopsy was 8 years (IQR 4.5-11) and 5.5 months (IQR 2.5-7), respectively. Seven patients had elevated levels of vWF in serum around time of biopsy (median 245%, IQR 203-302). All but 1 biopsy was performed prior to initiation of immunosuppressive therapy. Immunofluorescence staining showed a superficial and deep perivascular inflammatory cell infiltrate that co-localized with vWF in all tissues. There was expression of vWF in the extravascular tissue of patients with JScl co-localizing with collagen III in the reticular dermis (Figures 1 and 2). In comparison, vWF expression was restricted to the endothelium and did not co-localize with collagen in the dermis of patients with JDM (Figure 3). Patients with SSc had higher expression of vWF as compared to patients with LS.

Conclusions : vWF may participate in the pathogenesis of cutaneous inflammatory conditions. We have demonstrated that vWF co-localizes with cellular inflammatory infiltrates in the perivascular areas and in the dermis of patients with JScl and JDM. We additionally speculate that vWF may participate in the pathogenesis of fibrosing skin diseases based on evidence of increased extravascular expression in the tissue of patients with JScl (vs. JDM), and its co-localization with collagen. vWF expression intensity in the dermis of JScl patients may relate to disease extension (SSc vs. LS).

This study was approved by the Albert Einstein College of Medicine's Institutional Review Board by expedited review under 45 CFR 46.110 and 21 CFR 56.110 as the research fits into the following category: Category 5: Research involving materials (data, documents, records, or specimens) that have been collected, or will be collected solely for nonresearch purposes (such as medical treatment or diagnosis). This submission was approved with the following stipulation: The waiver of informed consent and HIPAA authorization were approved. IRB approval number 2017-8543.

**Fig. 1 (abstract A63).**
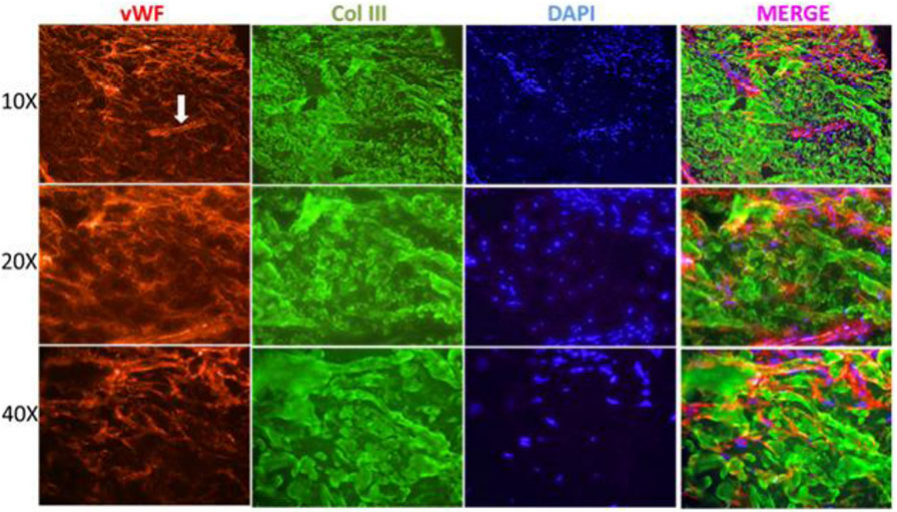
Double Immunofluorescence Staining for vWF and Collagen III in the Skin of a Patient with Diffuse SSc. Cuts of the reticular dermis are shown at different magnifications. There is increased collagen expression (green) throughout the reticular dermis that diffusely co-localize with increased expression of vWF (red). Vessels (arrow) stain at a higher intensity due to the presence of vWF in the subendothelium. A superficial and deep perivascular inflammatory cell infiltrate (blue) is also found co-localizing with vWF in areas of higher vWF expression

**Fig. 2 (abstract A63).**
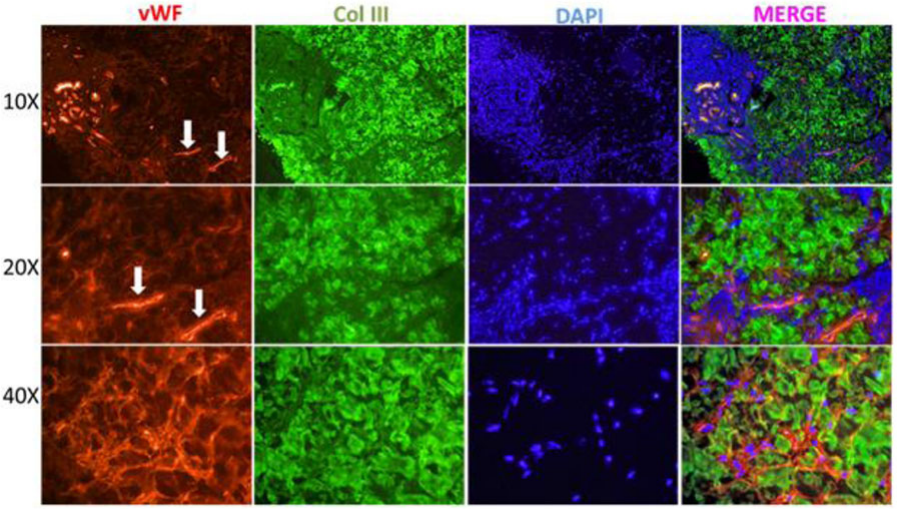
Double Immunofluorescence Staining for vWF and Collagen III in the Skin of a Patient with LS. Cuts of the reticular dermis are shown at different magnifications. There is increased collagen expression (green) throughout the reticular dermis that diffusely co-localize with increased expression of vWF (red). Vessels (arrow) stain at a higher intensity due to the presence of vWF in the subendothelium. A superficial and deep perivascular, and interstitial inflammatory cell infiltrate (blue) is also found co-localizing with vWF in areas of higher vWF expression

**Fig. 3 (abstract A63).**
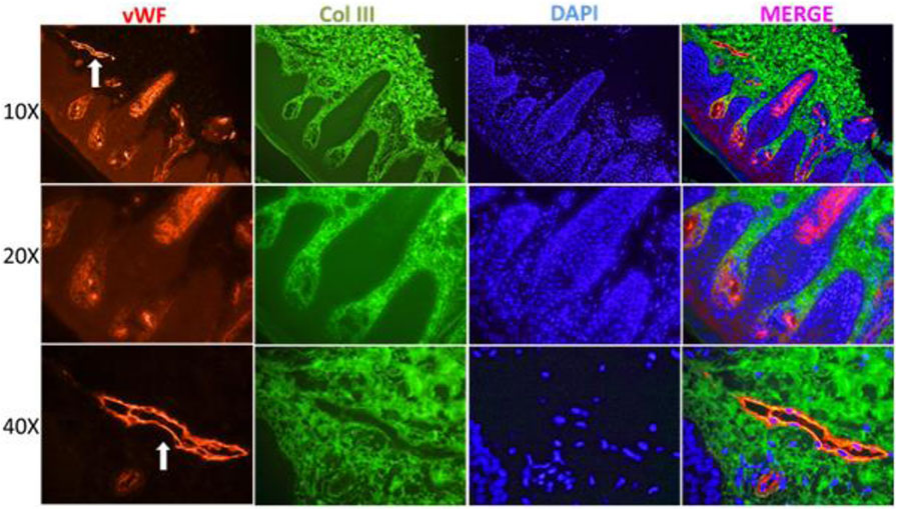
Double Immunofluorescence Staining for vWF and Collagen III in the Skin of a Patient with JDM. Cuts of the reticular and papillary dermis are shown at different magnifications. Expression of collagen III (green) is seen in the reticular dermis without co-localizing with vWF (red). Vessels (arrow) stain at a higher intensity due to the presence of vWF in the subendothelium. There is an inflammatory infiltrate at the dermal papillae and at the periphery of the vessels that co-localizes with vWF

## A64 Incidence of hypercalciuria and hematuria in Juvenile Idiopathic Arthritis

### Sarah Paul^187^, Kimberly Majors^189^, Barbara Ostrov^8^, Lisabeth Scalzi^187^

#### ^187^Penn State Children's Hospital, Hershey, PA, USA; ^189^Penn State Medical Center, Hershey, PA, USA; ^8^Albany Medical College, Albany, NY, USA

##### **Correspondence:** Sarah Paul

Background : Microscopic hematuria and hypercalciuria have previously been reported as a complication of Juvenile Idiopathic Arthritis (JIA). However, these studies are outdated, published prior to current diagnostic and therapeutic approaches. After recent experience with two patients with JIA and nephrolithiasis due to hypercalciuria, we aimed to readdress this concern by studying the incidence of microscopic hematuria and hypercalciuria in JIA. We wished to compare these results to the studies performed in 19851 and 19982 to determine differences in the incidence of hypercalciuria and hematuria in JIA in the current era of diagnosis and treatment.

Methods : 33 patients ages 4-18 years, fulfilling the criteria for the ILAR classification of subtypes of JIA, were identified and consented at the pediatric rheumatology clinic of Penn State Children’s Hospital. A random urine creatinine, calcium, and urinalysis was collected for all patients. Calcium:creatinine ratio was calculated for determination of hypercalciuria. A normal Ca:Cr ratio is less than 0.21 mg/dL. A brief survey was also performed to determine any family or personal history of nephrolithiasis, and any urinary symptoms. A chart review confirmed the date of diagnosis, disease activity, and concurrent medications taken at the time the urine sample was collected.

Results : Patients’ age ranged from age 6.3 to 18.1 (x=13.8 ) +/- , with 20 females (61%) and 13 males (39%). Two patients had a family history of nephrolithiasis but no patients had kidney stones during the study. Patients were taking the following therapies: NSAID -11; methotrexate - 8; sulfasalazine - 6; anti-TNF agent - 4; tocilizumab -3; canakinumab -1; abatacept-2; and secukinumab -1. 3 patients were taking methotrexate plus a biologic. No child was taking corticosteroids. 2 of 33 patients were found to be hypercalciuric (6%), 1 with sJIA and 1 with ERA, while only 1, with jPsA, had hematuria (3%). The joint counts of the patients with hypercalciuria were 1 and 0, while the joint count of the patient with hematuria was 0. The average calcium:creatinine ratios can be seen in Table 1. ANOVA analysis determined no significant differences between the average calcium:creatinine ratios of the JIA subtypes (p=.46).

Conclusions : Our results show a much lower proportion of hypercalciuria compared to the studies performed in 1985and 1998 which was identified as 32% and 46%, respectively. Our study also demonstrates a lower proportion of hematuria, with only one patient (3%) having hematuria in our study compared to 24% and 21% in the 19851 and 19982 studies, respectively. The inflammatory cytokines IL-1, IL-6, and TNF-α play a role in the bone resorption associated with idiopathic hypercalciuria and subtypes of JIA3,4 As these inflammatory markers are a major target for biologic DMARDs currently used to treat JIA, we propose the change in treatment approaches and low inflammation levels in our population may be a potential reason for the change in results compared to 20 years ago.

This study was approved by Penn State University’s Internal Review Board, approval number 00007386.

**Table 1 (abstract A64).** Average calcium:creatinine ratios of the subtypes of JIA

Subtype	N	Average Age	Average Ca:creatine ratio	Standard deviation	Hypercalciuria	Hematuria
Enthesitis-related	10	14.4	0.111	0.100	1	0
Oligoarticular	8	10.8	0.109	0.042	0	0
Polyarticular	6	15.2	0.081	0.060	0	0
Psoriatic	5	16.1	0.105	0.048	0	1
Systemic	3	12.3	0.187	0.056	1	0
Undifferentiated	1	16.0	0.149	n/a	0	0
Total	33	13.8	0.112	0.071	2	1

## A65 Examining Risk Factors Associated with Depression and Anxiety in Youth with Systemic Lupus Erythematosus

### Tamar Rubinstein^9^, Marija Dionizovik-Dimanovski^50^, Chelsey Smith^61^, Jordan Jones^61^, Julia Harris^61^, Raphael Kraus^118^, Martha Rodriguez^197^, Lauren Faust^227^, Alaina Davis^308^, Manda Mitchell^308^, Melissa Tesher^271^, Rebecca Puplava^271^, Karen Onel^120^, Bhupinder Nahal^245^, Sangeeta Sule^63^, Emily vonScheven^244^, Andrea Knight^229^, for the CARRA SLE Subcommittee

#### ^9^Albert Einstein College Of Medicine, Children's Hospital At Montefiore, Bronx, NY, USA; ^50^Children's Hospital at Montefiore, Bronx, NY, USA; ^61^Children's Mercy Hospitals and Clinics, Kansas City, MO, USA; ^118^Hospital for Sick Children/ University of Toronto, Toronto, ON, Canada; ^197^Riley Hospital for Children at University of Indiana, Indianapolis, IN, USA; ^227^The Children's Hospital of Philadelphia, Philadelphia, PA, USA; ^308^Vanderbilt University Medical Center, Nashville, TN, USA; ^271^University of Chicago Medicine Comer Children's Hospital, Chicago, IL, USA; ^120^Hospital for Special Surgery, New York, NY, USA; ^245^UCSF Benioff Children's Hospital, San Francisco, CA, USA; ^63^Children's National Health System, Washington, DC, USA; ^244^University of California, San Francisco, San Francisco, CA, USA; ^229^The Hospital for Sick Children, Toronto, ON, Canada

##### **Correspondence:** Tamar Rubinstein

Background : Depression and anxiety are prevalent in youth with systemic lupus erythematosus (SLE). Increasing our understanding of associated disease and sociodemographic characteristics may help identify patients at particular risk. A secondary analysis was conducted from our previous study designed to assess the acceptability of screening for depression and anxiety in youth with SLE, to determine factors associated with positive screens.

Methods : In a multi-center study of 6 collaborating CARRA clinics, patients with SLE ages 12-21 years were consecutively screened with the Patient Health Questionaire-9 (PHQ9) for depression and the Generalized Anxiety Disorder 7-item scale (GAD7) for anxiety. Sociodemographic, patient and family history, and disease characteristics were collected, including the Systemic Lupus Erythematosus Disease Activity Index (SLEDAI), presence of each American College of Rheumatology (ACR) criteria, SLE complications, and overlapping fibromyalgia/amplified pain. Patient reported outcomes (PROs) for depression, anxiety, fatigue, pain, and physical and social functioning were collected using the Patient Reported Outcomes Measurement Information System. Chi square and Wilcoxon rank-sum tests examined the relationship between patient characteristics and positive screens and Spearman correlation analyses examined the relationship between PROs, SLEDAI, steroid doses, PHQ9 and GAD7 scores. All patients met ACR SLE Classification Criteria or SLICC Classification Criteria.

Results : Eighty patients were screened. The median age was 17.1 years with median disease duration of 2.4 years. The majority were female (89%), and Black (44%) or Hispanic (25%); 44% of patients came from zipcodes where the median annual household income was <$50,000. Of those screened, 20% screened positive for depression, 16% for anxiety, and 11% had suicidal ideation. No sociodemographic or disease features, including disease duration, were associated with positive screens for depression or anxiety. Family history of psychiatric disease was associated with positive depression screens, but not positive anxiety screens. Patient history of psychological disease was not associated with positive screens for depression or anxiety. PHQ9 and GAD7 scores were highly correlated to PROs for fatigue, pain, pain interference, depression and anxiety. PHQ9 and GAD7 scores moderately correlated with impaired mobility and negatively correlated with peer relationships. PHQ9 and GAD7 scores did not correlate with SLEDAI score, steroid dose, or disease duration (Table 1).

Conclusions : No specific sociodemographic or patient characteristic were found to be associated with positive depression or anxiety screens among youth with SLE. While depression and anxiety scores were not correlated with disease activity measures, they were highly correlated with several PROs. Our findings support the need for a broad screening approach for youth with SLE, and a potential role for PRO assessment in interventions to improve psychosocial health.

This study was approved by the Albert Einstein College of Medicine Institutional Review Board (IRB 2016-7164).

This project was funded by a CARRA-Arthritis Foundation Small Grant Award. The authors wish to acknowledge the ongoing Arthritis Foundation financial support of CARRA. Einstein-Montefiore REDCap is supported by the Harold and Muriel Block Institute for Clinical and Translational Research at Einstein and Montefiore (1UL1TR002556). Tamar Rubinstein’s research is funded by the Rheumatology Research Foundation K-Bridge Award.

**Table 1 (abstract A65).** Correlations with depression (PHQ9) and anxiety (GAD7) scores in youth with SLE

	PHQ9 score	GAD7 score
GAD7 score	0.7, p<0.0001	1.00
SLEDAI score	0.07, p=0.6	0.2, p=0.2
Steroid dose	-0.05, p=0.7	-0.1, p=0.3
Disease duration	0.06, p=0.6	0.02, p=0.9
PROMIS measures:		
Worse depression	0.7, p<0.0001	0.7, p<0.0001
Worse anxiety	0.6, p<0.0001	0.8, p<0.0001
Worse fatigue	0.7, p<0.0001	0.6, p<0.0001
Stronger peer relationships	-0.3, p=0.002	-0.4, p=0.009
Lower physical functioning	0.4, p=0.0001	0.4, p=0.0004
Higher pain interference	0.5, p<0.0001	0.6, p<0.0001
Higher pain severity	0.5, p<0.0001	0.4, p=0.0003

PHQ9 (Patient Health Questionnaire-9) scores ranged from 0-27; GAD7 (Generalized Anxiety 7-Index) scores ranged from 0-21. SLEDAI (SLE Disease Activity Index 2K Modification) scores ranged from 0-20 (of a possible range 0-105). Steroid dose was current daily dose. PROMIS (Patient Reported Outcome Measurement Information System) scores were measured as T-scores, except for pain severity measured on a scale of 1-10. Spearman rho correlation coefficients shown

## A66 Evaluating Disease Activity Outcomes for Juvenile Idiopathic Arthritis Across the Pediatric Rheumatology Care and Outcomes Improvement Network (PR-COIN)

### Emily Smitherman^258^, Bin Huang^184^, Ron Laxer^119^, Catherine Bingham^188^, Cagri Yildrim Toruner^150^, Beth Gottlieb^69^, Jennifer Weiss^115^, Tzielan Lee^214^, Sheetal Vora^138^, Jon Burnham^55^, Julia Harris^61^, Judyann Olson^144^, Mileka Gilbert^145^, Michelle Batthish^143^, Michael Shishov^190^, Dustin Fleck^280^, Esi Morgan^275^

#### ^258^University of Alabama at Birmingham, Birmingham, AL, USA; ^184^Pediatric Rheumatology Collaborative Study Group (PRCSG), Cincinnati Children’s Hospital Medical Center, Cincinnati, OH, USA; ^119^Hospital for Sick Kids, Toronto, ON, Canada; ^188^Penn State Health Children's Hospital, Hershey, PA, USA; ^150^Nationwide Children's Hospital, Columbus, OH, USA; ^69^Cohen Children's Medical Center of New York, Queens, NY, USA; ^115^Hackensack University Medical Center, Hackensack, NJ, USA; ^214^Stanford University School of Medicine, Stanford, CA, USA; ^138^Levine Children's Hospital, Charlotte, NC, USA; ^55^Children's Hospital of Philadelphia, Philadelphia, PA, USA; ^61^Children's Mercy Hospitals and Clinics, Kansas City, MO, USA; ^144^Medical College of Wisconsin, Wauwatosa, WI, USA; ^145^Medical University of South Carolina, Charleston, SC, USA; ^143^McMaster Children's Hospital, Hamilton, ON, Canada; ^190^Phoenix Children's Hospital, Phoenix, AZ, USA; ^280^University of Michigan, Ann Arbor, MI, USA; ^275^University of Cincinnati College of Medicine, Cincinnati, OH, USA

##### **Correspondence:** Emily Smitherman

Background : Clinical remission is widely accepted as the primary target outcome for juvenile idiopathic arthritis (JIA). In practice, JIA disease activity can be measured using the ACR provisional criteria for Clinical Inactive Disease (CID). PR-COIN is a quality improvement collaborative that serves as a sample of pediatric rheumatology centers in North America. Through PR-COIN, the percentage of JIA patients in CID is prospectively measured, and variation between centers has been previously observed. The objective of the current study was to evaluate patient-level sociodemographic, clinical, and medication factors that may be associated with center-level variation.

Methods : This study used cross-sectional data provided by PR-COIN that was collected by PR-COIN centers from March 2015 to May 2016. For each patient, variables from the last encounter were extracted (Table 1). Patients with disease duration less than 9 months were excluded. Patients were grouped by CID status (inactive vs active disease). Descriptive statistics were calculated for the total population and subgroups. Multiple two-group comparisons were performed, including chi-square for categorical variables and independent t-tests for continuous variables. Missing data were excluded with each univariate analysis. Analyses were performed using R software.

Results : There were 2751 patients eligible for analysis from 14 centers, with 33 to 394 patients per center. We identified 1249 patients with inactive disease (47%) and 1428 patients with active disease (53%) across the population (Table 2). Differences were noted in patient age, ILAR subtype, and patient-reported outcome measures between patients with inactive and active disease. Differences were noted across all disease activity measures, as would be expected based on CID definition. Overall, more medication usage was observed in patients with active disease across multiple medication categories.

Conclusions : This study demonstrates initial differences in sociodemographic, clinical, and medication characteristics between patients with inactive and active JIA. Further analysis is needed to adjust center-level measures of JIA disease activity using these differences in patient case-mix. By using population data to identify predictors of inactive disease in JIA, there is potential to develop and implement targeted clinical interventions that can directly impact care delivery and lead to improved outcomes in JIA.

This study was approved by the Institutional Review Board at Cincinnati Children’s Hospital Medical Center as no greater than minimal risk.

**Table 1 (abstract A66).** Patient-Level Variables for Analysis

Sociodemographic	ESR and CRP elevation
Age	Active joint count
Gender	MD global assessment
Race	
Insurance type	Patient-Reported Measures
	Patient global assessment
Clinical Characteristics	Arthritis-related pain score
Disease duration	Childhood Health Assessment Questionnaire (CHAQ) score
ILAR subtype	
ANA status	Current Medications
	Non-biologic DMARD use
Disease Activity Measures	Biologic DMARD use
Active uveitis	NSAID use (daily vs intermittent)
Active systemic features	Glucocorticoid use
Morning stiffness > 15 min	- Intra-articular vs systemic vs ophthalmic

**Table 2 (abstract A66).** Characteristics of total population and patients divided into subgroups. Results reported as frequency (%) or mean (standard deviation)

Variable	All Patients (n = 2751)	Patients w Inactive Disease (n = 1249)	Patients w Active Disease (n = 1428)
SOCIODEMOGRAPHIC			
Age (years)	12.4 (4.7)	**11.8 (4.8)**	**12.9 (4.6)**
Female	1675/2356 (71%)	723/1024 (71%)	929/1304 (71%)
Race (White)	1885/2207 (85%)	824/970 (85%)	1045/1216 (86%)
Insurance Status (Private)	1536/1769 (87%)	713/812 (88%)	814/946 (86%)
CLINICAL CHARACTERISTICS			
Disease Duration (months)	68.1 (49.1)	69.0 (48.4)	67.2 (49.4)
ILAR Subtype			
Polyarticular, RF (-)	790 (29%)	**350 (28%)**	**420 (29%)**
Oligoarticular, persistent	768 (28%)	**417 (33%)**	**330 (23%)**
Enthesitis related arthritis	332 (12%)	**115 (9%)**	**207 (14%)**
Oligoarticular, extended	286 (10%)	**138 (11%)**	**136 (10%)**
Psoriatic arthritis	184 (7%)	**61 (5%)**	**121 (8%)**
Systemic arthritis	174 (6%)	**87 (7%)**	**83 (6%)**
Polyarticular, RF (+)	157 (6%)	**55 (4%)**	**100 (7%)**
Undifferentiated	54 (2%)	**25 (2%)**	**27 (2%)**
ANA Positive	1339 (49%)	611 (49%)	691 (49%)
DISEASE ACTIVITY MEASURES			
Active Uveitis	133/2393 (6%)	**0**	**129/1286 (10%)**
Systemic Features	21 (<1%)	**0**	**21 (1%)**
Morning Stiffness > 15 min	1934/2389 (81%)	**0**	**452/1303 (35%)**
Elevated ESR / CRP	204 (7%) / 139 (5%)	**0**	**204 (14%) / 139 (10%)**
Active Joint Count	0.9 (2.0)	**0**	**1.8 (2.5)**
MD Global Assessment	1.0 (1.4)	**0.03 (0.1)**	**1.8 (1.5)**
PATIENT-REPORTED MEASURES			
Patient Global Assessment (0-10)	1.7 (2.3)	**0.74 (1.5)**	**2.6 (2.5)**
Pain Score (0-10)	2 (2.5)	**0.8 (1.5)**	**2.9 (2.8)**
CHAQ	0.24 (0.44)	**0.11 (0.26)**	**0.36 (0.52)**
MEDICATIONS			
Non-biologic DMARDs	1059 (38%)	**399 (32%)**	**640 (45%)**
Methotrexate	914 (86%)	350 (88%)	549 (86%)
Leflunomide	68 (6%)	24 (6%)	43 (7%)
Sulfasalazine	51 (5%)	16 (4%)	34 (5%)
Hydroxychloroquine	43 (4%)	7 (2%)	34 (5%)
Other	14 (1%)	5 (1%)	10 (2%)
More than one	33 (3%)	3 (<1%)	30 (5%)
Biologic DMARDs	1101 (40%)	**434 (35%)**	**684 (48%)**
All Anti-TNF	863 (31%)	**350 (28%)**	**498 (35%)**
Etanercept	420 (38%)	191 (44%)	223 (33%)
Adalimumab	331 (30%)	125 (29%)	202 (30%)
Infliximab	105 (10%)	32 (7%)	68 (10%)
Certolizumab	9 (<1%)	5 (1%)	4 (<1%)
Golimumab	8 (<1%)	3 (<1%)	5 (<1%)
Tocilizumab	97 (9%)	32 (7%)	64 (9%)
Abatacept	80 (7%)	20 (5%)	60 (9%)
Anakinra	23 (2%)	13 (3%)	9 (1%)
Canakinumab	21 (2%)	12 (3%)	8 (1%)
Rituximab	6 (<1%)	1 (<1%)	4 (<1%)
Tofacitinib	4 (<1%)	0	4 (<1%)
Other	2 (<1%)	2 (<1%)	0
More than one	5 (<1%)	2 (<1%)	3 (<1%)
NSAIDs			
Daily	770 (28%)	**204 (16%)**	**551 (29%)**
Intermittent	345 (13%)	150 (12%)	195 (14%)
Glucocorticoids			
Intra-articular	201 (7%)	**46 (4%)**	**147 (10%)**
Systemic	84 (3%)	**17 (1%)**	**63 (4%)**
Ophthalmic	119 (4%)	**19 (2%)**	**94 (7%)**

A chi-square or t-test was performed to compare characteristics of patients with inactive and active disease. Results with p < 0.05 are bolded

## A67 A Single Center Study of the Presentation, Disease Course and Treatment of Childhood Onset Rheumatoid Arthritis

### Aimee Hersh^294^, Sampath Prahalad^99^, Karen James^294^, C.J. Inman^294^, Sara Stern^294^, John Bohnsack^295^

#### ^294^ University of Utah, Salt Lake City, UT, USA; ^99^Emory University and Children's Healthcare of Atlanta, Atlanta, GA, USA; ^295^University of Utah Health Sciences Center, Salt Lake City, UT, USA

##### **Correspondence:** Aimee Hersh

Background : Approximately 10% of all patients with Juvenile Idiopathic Arthritis (JIA) have Polyarticular (Rheumatoid-Factor Positive) JIA, also called Childhood Onset Rheumatoid Arthritis (CORA). There is limited data on the long-term outcomes of this JIA subtype which is likely to have disease extension into adulthood. We sought to describe the presentation, disease course and treatment for our single center cohort of CORA patients.

Methods : The Intermountain States Database of Childhood Rheumatic Diseases (ISDCRD) is a longitudinal cohort study started at the University of Utah in 1997 which currently includes >700 patients with all JIA subtypes. Clinical data collected in the ISDCRD includes date of diagnosis, symptom duration prior to diagnosis, ILAR subtype and the initial clinical presentation including joint count and relevant laboratory studies. The majority of ISDCRD participants also provide DNA samples. CORA subjects were identified in the ISDCRD by ILAR subtype. A detailed chart review was completed to describe the treatment course and outcomes from diagnosis until the subject was transferred or lost to follow-up from the pediatric rheumatology clinic. Descriptive statistics were used. Study approval was obtained from the University of Utah Institutional Review Board.

Results : 50 CORA subjects were identified in the ISDCRD; subjects were diagnosed between 1995-2015. 86% of the cohort is female. The race/ethnicity was 80% Caucasian, 15% Hispanic and 4% Asian. The median age of symptom onset was 11.1 years (range 1.1- 16 years) and median age of diagnosis was 12.3 years (range 3.1- 17.6 years). The median duration of clinic follow-up was 5.3 years (range 0-12.8) and the median current age at the time of the chart review was 24.7 years (range 9.4-34.3); 88% are >18 years old. 19 (38%) subjects had up to a third degree family history of RA. 100% of subjects had a positive rheumatoid factor, 80% were also anti CCP antibody positive. The median number of joints affected at diagnosis was 13 (range 1-44). Eight (16%) patients developed rheumatoid nodules during the follow-up period. 42 (84%) of patients had joint radiographs at diagnosis or during follow-up and of those 33 (79%) had erosions on at least one radiograph. Looking broadly at the treatment course, 10 (20%) patients responded to Methotrexate alone, 19 (38%) responded to a single biologic therapy (most commonly a Tumor Necrosis Factor alpha inhibitor), and 21 (42%) had a failure of one or more biologic therapies.

Conclusions : In this single center cohort study of CORA patients, there was a high frequency of radiographic joint damage and nearly half required one or more biologic therapies. Given the chronicity of this JIA subtype better characterization of long-term outcomes is needed. A follow-up study is underway to determine outcomes in adulthood.

Study approval was obtained from the University of Utah Institutional Review Board.

## A68 Improving Care for Children with Autoinflammatory Diseases: CARRA-Sponsored Network Building

### Jocelyne Beelen^75^, Eyal Muscal^26^, Susanne Benseler^91^, Rae S.M. Yeung^292^, Marinka Twilt^91^, for the CARRA Autoinflammatory Disease Workgroup

#### ^75^Cumming School of Medicine, University of Calgary, Calgary, AB, Canada; ^26^Baylor College of Medicine/Texas Children's Hospital, Houston, TX, USA; ^91^Division Rheumatology, Alberta Children’s Hospital, University of Calgary, Calgary, AB, Canada; ^292^University of Toronto and The Hospital for Sick Children, Toronto, ON, Canada

##### **Correspondence:** Jocelyne Beelen

Background : Over the last 25 years, our knowledge about childhood diseases presenting with non-infectious, ‘sterile’ inflammatory fever attacks has increased and changed our approach to treatment. In 1999, the concept of "autoinflammation" was proposed by Dan Kastner and colleagues as the molecular mechanisms other than autoimmunity as cause for known periodic fever syndromes. Autoinflammatory conditions are described as a genetically heterogeneous group of rheumatologic diseases caused by single gene mutations and driven by abnormal activation of the innate immune system. Innate immunity research and discoveries have led to the identification of disease causing genes associated with an excessive cytokine release. These diseases were associated with high mortality and morbidity, but increased recognition and early treatment can prevent organ damage and increase good outcomes. Often, life-long control of ongoing inflammation is needed to improve, stabilize, and minimize clinical symptoms and prevent damage. As with all orphan diseases, clinical trials included limited numbers of patients treated under controlled circumstances, so long-term real-life data cannot be extrapolated. Existing registries are treatment specific pharmacosurveillance registries, at specialized centers, with regimented data collection. Data from these traditional registries is not translatable to clinical practice. We propose to begin the first population based disease registry that will include patients from all CARRA Registry sites in North America. Real-life data will demonstrate the variability in care and treatment patterns, leading to improved outcomes for patients with AID at CARRA registry sites, providing rapid knowledge translation through the CARRA infrastructure.

Methods : CARRA small grant support has funded infrastructure for the improvement of care for children with autoinflammatory diseases (AID) through development of a CARRA autoinflammatory registry and the environment to include CTPs for the treatment of CAPS in the AID registry. Funds were provided for the development of the autoinflammatory network and of the AID registry. CARRA funds have supported a research assistant at the Alberta Children’s Hospital whose responsibilities included: assistance in development of AID registry data fields, set-up meetings, and literature search and review on AID registries.

Results : The autoinflammatory group has reached out to the CARRA PFAPA and CRMO group to develop one AID registry including all diseases. A first iteration of data-elements has taken place and will be finalized at the 2019 CARRA meeting. Conversation with the autoinflammatory program development group has led to inclusion of outcome measures and disease activity tools.

Conclusions : With the CARRA small grant we were able to establish an autoinflammatory network including the CARRA working groups that deal with AID to pursue the establishment of an AID registry. Continued collaboration with all partners is necessary to improve the care of children with AID.

Acknowledgements: This project was funded by a CARRA – Arthritis Foundation grant. The authors wish to acknowledge the ongoing Arthritis Foundation financial support of CARRA.

## A69 Health Care Providers’ Views of Decision-making for Pain Management among Youth with Juvenile Idiopathic Arthritis: Preliminary Results from Interviews

### Karine Toupin April^54^, Jennifer Stinson^240^, Adam Huber^77^, Deema Couchman^283^, Marco Benedetto Ragusa^283^, Hannah Sachs^53^, Aditi Sivakumar^53^, Isabelle Gaboury^254^, Ciaran Duffy^54^, Esi Morgan^275^, Lucie Brosseau^283^, Linda Li^260^, Bill Brinkman^66^, Marg Bisch^52^, Janice Cohen^52^, Elizabeth Stringer^133^, France Légaré^255^, Laurie Proulx^39^, Sabrina Cavallo^253^, Paul Fortin^44^, Peter Tugwell^284^

#### ^54^Children's Hospital of Eastern Ontario Research Institute and University of Ottawa, Ottawa, ON, Canada; ^240^Universty of Toronto (SickKids), Toronto, ON, Canada; ^77^Dalhousie University and IWK Health Centre, Halifax, NS, Canada; ^283^University of Ottawa, Ottawa, ON, Canada; ^53^Children's Hospital of Eastern Ontario Research Institute, Ottawa, ON, Canada; ^254^Université de Sherbrooke, Sherbrooke, QC, Canada; ^275^University of Cincinnati College of Medicine, Cincinnati, OH, USA; ^260^University of British Columbia, Vancouver, BC, Canada; ^66^Cincinnati Children's Hospital, Cincinnati, OH, USA; ^52^Children's Hospital of Eastern Ontario, Ottawa, ON, Canada; ^133^IWK Health Centre, Dalhousie University, Halifax, NS, Canada; ^255^Université Laval, Québec City, GC, Canada; ^39^Canadian Arthritis Patient Alliance, Canada; ^253^Université de Montréal, Montréal, GC, Canada; ^44^Centre de Recherche du Centre Hospitalier Universitaire de Québec, Québec City, GC, Canada; ^284^University of Ottawa and Ottawa Hospital Research Institute, Ottawa, ON, Canada

##### **Correspondence:** Karine Toupin April

Background : Youth with juvenile idiopathic arthritis (JIA) face various treatment decisions. However, no studies have fully explored decision-making needs when choosing how to manage pain. Our study explored health care providers’ (HCP) views of decision-making for pain management among youth with JIA and their parents.

Methods : Semi-structured face-to-face or online individual interviews were conducted with HCPs using a qualitative descriptive study design. Using purposive sampling, we recruited HCPs who work in various rheumatology clinics in Canada and the United States. The interview guide was based on the Ottawa Decision Support Framework. Interviews were audiotaped, transcribed verbatim and analyzed using simple content analysis.

Results : 10 HCPs, comprised of pediatric rheumatologists (n=4), physical therapists (n=3), pediatric rheumatology nurses (n=2) and an occupational therapist (n=1) participated in interviews. Themes are summarized below: (1) Views on pain: the majority of HCPs associated JIA pain with disease activity, pain amplification or mechanical pain. Most HCPs agreed that pain is common in JIA, although they mentioned difficulty qualifying pain due in part to discrepancies between youths’ and parents’ report of pain and the youths’ difficulty in remembering pain over time. (2) Treating pain: rheumatologists’ first goal was to control disease activity, which they felt usually would address the pain. While allied HCPs usually tended to focus on symptom management and improving function, most rheumatologists focused their interventions on medication to control disease activity, followed by medication and non-pharmacological options to reduce residual pain. HCPs recommended a range of non-pharmacological treatments but this varied among HCPs, with the most common ones being physical activity, heat and cold packs, and stretching. HCPs shared their clinical experience and links to trusted websites to help guide families to manage pain. They acknowledged that families preferred non-pharmacological treatments for fear of overmedicating; a sentiment echoed by most HCPs. (3) Decision-making needs: HCPs voiced a lack of knowledge about available options for pain management and about their evidence, especially for non-pharmacologic options. Some also mentioned limited evidence for these options. HCPs reported that they would benefit from a tool to help objectively qualify a youth’s pain and to present evidence-based information on a range of treatments that match each youth’s preferences and values to engage in a collaborative approach to pain management with youth and their parents.

Conclusions : Findings of interviews suggest an unmet need for evidence-based information on non-pharmacological treatment options for pain management for HCPs to share with youth and their parents. A decision-making tool may enable HCPs to work with youth and families to better qualify their pain and identify evidence-based treatment options to make informed and value-based decisions.

The Children’s Hospital of Eastern Ontario Research Ethics Board approved this study (protocol 16/100X) . Participants were asked to complete a consent form prior to being interviewed.

## A70 A Potential Role of Early Sjögren’s Antibodies as a Biomarker in Seronegative Children with Sjögren Syndrome

### Akaluck Thatayatikom^212^, Indraneel Bhattacharyya^71^, Melissa Elder^212^, Renee Modica^212^, Seunghee Cha^71^

#### ^212^Shands Children's Hospital, College of Medicine, University of Florida, Gainesville, FL, USA; ^71^College of Dentistry, University of Florida, Gainesville, FL, USA

##### **Correspondence:** Akaluck Thatayatikom

Background : Juvenile Sjögren syndrome (JSS) is an uncommon systemic autoimmune disease in children that primarily affects the salivary and lacrimal glands with variable systemic involvement. The lack of predictive laboratory screening, standardized imaging and diagnostic consensus criteria for JSS contribute to misdiagnosis or delay in diagnosis and treatment, especially children with negative SSA and SSB antibodies. Recent studies in adult patients with SS revealed early Sjögren’s antibodies may be useful as a biomarker for identifying patients with an early stage of SS. This potential biomarker has not been studied in children.

Methods : This is a descriptive study of children who were evaluated in Pediatric Rheumatology Clinic and Oral Medicine Clinic at the University of Florida between January 2018-December 2018 and had a diagnosis of JSS with negative SSA and SSB antibodies. Clinical manifestations, minor salivary gland biopsy (MSGBx), point-of-care salivary gland ultrasound (SGUS) and the early Sjögren antibody panel including salivary protein-1 (SP-1), carbonic anhydrase VI (CA-6) and parotid specific protein (PSP) were obtained.

Results : We identified three children with JSS based on abnormal MSGBx, positive early Sjögren autoantibodies in spite of negative SSA and SSB antibodies. Two out of 3 children had abnormal SGUS as well as abnormal sialometry indicating asymptomatic xerostomia. The anti-CA-6 antibody was the most common early Sjögren antibody found in this series of children (Table 1).

Conclusions : Children with JSS may present with glandular and/or extraglandular signs/symptoms and early Sjogren’s autoantibody positivity despite negative SSA and SSB antibody. Early Sjögren autoantibodies may also be correlated with abnormal MSGBx. Although these results may be limited due to the small number of subjects, our findings indicate that early Sjögren antibodies can potentially serve as a biomarker for JSS. Further evaluation of the specificity and sensitivity early Sjögren antibodies in children with JSS is warranted.

University of Florida IRB does not require an approval if the study is limited for 3 cases. Parents of the three subjects provided the consent to participate in the study.

**Table 1 (abstract A70).** Clinical manifestations, major salivary gland ultrasonography and diagnostic test results

Age at Dx (yr)	Age at onset (yr)	Glandular symptoms	Extra-glandular symptoms	Sialometry	MSGBx (Focus score)	SGUS score*	ANA	SSA & SSB	SP-1	CA-6	PSP
5	2	Recurrent parotitis	None	Abnormal	2	2	Neg	Neg	36.1 (IgM)	33.2 (IgG)	Neg
11	6	None	Gastroparesis, arthralgia, fatique	Normal	>1	0	Neg	Neg	Neg	31.1 (IgG)	Neg
15	11	History of parotitis	Raynaud’s, arthralgia, recurrent aseptic meningitis	Abnormal	>4	2	>1 :2560	Neg	>160 (IgM)	73.1 (IgG) >160 (IgM)	>160 (IgM)

## A71 IVIg-Refractory Kawasaki Disease: Distilling the Literature to Predict Outcomes

### Courtney Crayne, Timothy Beukelman

#### University of Alabama at Birmingham, Birmingham, AL, USA

##### **Correspondence:** Courtney Crayne

Background : Characterized by fever and mucocutaneous features, Kawasaki Disease (KD) is an acute, self-limited medium vessel vasculitis most commonly affecting infants and young children. Coronary artery aneurysms are a well-recognized complication of KD, occurring in roughly 25% of untreated disease. If IVIg (2g/kg) is given during the first 10 days of fever, coronary abnormalities risk during the first 30 days is reduced from ~25% with aspirin alone to ~5%. Refractory KD, defined as the return of fever at least 36 hours and <7 days following completion of initial IVIg infusion occurs in about 10-20%. Persistent fever is associated with a significantly higher risk of developing coronary artery aneurysms during the subacute phase. Evidence remains contradictory regarding next-line therapy and risk of cardiac sequelae. The objective of this study is to evaluate the efficacy and safety of three treatments [i.e. a second IVIg infusion, glucocorticoids (GC), and infliximab (IFX)] in patients with refractory KD using decision analysis methods.

Methods : A targeted review of literature published from 1990 to November 2017 was performed using PubMed, Embase, Cochrane, and ClinicalTrials.gov to extract outcome probabilities for a decision analysis model. The primary outcomes were response to treatment, defined as resolution of fever within 36 hours of treatment, and reduction in size of coronary artery lesions within 8 weeks. Eligibility criteria included articles written in English and original research, including cases or case series not previously reported. Basic laboratory science articles, data not specific to IVIg-refractory KD, previously reported data, and articles written in languages other than English were excluded. Expected values for each of the three treatments were calculated using previously reported data.

Results : Thus far, 9 manuscripts have been identified and reviewed. Four were prospective, randomized studies, and two were prospective, non-randomized. Three of the published studies were retrospective reviews. This combined cohort included 188 patients from Japan, 130 from North America, and 43 from Korea. In total, 209 patients received a second dose of IVIg, 89 received GC, and 63 received IFX. Treatment response was 72% with IVIg, 72% with GC and 89% with IFX. Overall, the total number of patients with coronary artery lesions was 17 (8.13%) in the second IVIg group, 10 (11.24%) in the GC group, and 24 (38.1%) in the IFX group. The number of patients with moderate-severe or persistent aneurysms was zero with IVIg, 7 with GC, and 3 with IFX. Estimated expected value of IVIg was 0.384, of GC was 0.16, and of IFX was 0.094.

Conclusions : Preliminary literature review suggests a second dose of IVIg is used more frequently in refractory KD and response to treatment is comparable to GC. The reported differences in reduction of coronary artery lesions among each treatment group is of unclear significance at this time. Preliminary expected value calculations suggest a second dose of IVIg has the best outcome return; however, this model does not account for medication costs or duration of hospital stay. This is an on-going analysis in the final stages of the targeted review and decision-analysis with plans to perform a cost-effective analysis using previously reported outcomes to determine the best second-line therapy for IVIg-refractory KD.

## A72 Decreasing Outpatient Telephone Calls by Standardizing the Obtainment and Review of Laboratory Tests: A Quality Improvement Initiative

### Lisa Buckley, Brian Nolan, Rosemary Peterson, Emily Liebling, Beth Rutstein, Julie Chase, Lisa Wiater, Danielle Dodson, Deborah Bieniakowski, Jon Burnham

#### Children's Hospital of Philadelphia, Philadelphia, PA, USA

##### **Correspondence:** Lisa Buckley

Background : Clerical work including answering telephone calls is consistently linked to physician burnout. At our institution, rheumatology physicians collectively answer an average of 15 telephone calls per day, 84% of which are answered by a single first-year fellow. From January 2016 to May 2018, lab results was the second most common reason for a telephone call and accounted for over a quarter of all outpatient telephone calls requiring a physician. Our goal was to decrease outpatient telephone calls by standardizing the obtainment and review of outpatient labs.

Methods : We identified 2 opportunities for improvement: 1) the process by which patients obtain routine medication toxicity and disease activity labs and 2) the communication of physician result review with nurses and families. In August 2018, we began a process shift in which routine labs are drawn 1-2 weeks prior to office visits so that results can be discussed in-person. An after-visit summary (AVS) smart phrase was modified to reflect this process change. Physicians were encouraged to discuss the AVS including this process change with families at the end of each visit. In September 2018, physicians began completing result notes to communicate their review of lab results with patients and nurses. These notes are released to families through the patient portal and are reviewable by the nurses in the event of a result-related telephone call. Outcome measures were monthly population-adjusted number of telephone calls and monthly proportion of telephone calls related to labs. Volume of calls was used as a surrogate for time spent on calls as accurately tracking time was found to be cumbersome and unreliable. Parent satisfaction was tracked intermittently through survey and parent representative feedback. Data were analyzed for special cause variation using statistical process control (SPC) methods.

Results : The monthly proportion of telephone calls related to lab results met special cause variation in November 2018 at which time the mean shifted from 12% to 8% of calls. We estimate that this is about 15 less phone calls per month. The population-adjusted number of telephone calls was down-trending but did not meet special cause during this period. Survey of parents showed that 100% (23/23) preferred the process of having labs performed prior to office visits. During the time period of August 2018 to January 2019, symptoms/illness was the most common reason for a telephone call followed by medication questions.

Conclusions : Although telephone calls related to laboratory tests significantly decreased following our interventions, overall phone calls were unchanged. Yet, the interventions were well-received and preferred by parents. Our data suggest that the decrease in lab calls was offset by an increase in medication-related calls, and that there are likely multiple additional contributing factors to high volume of telephone calls. Next steps include exploring these potential areas of improvement.

IRB approval was not needed as this was a quality improvement initiative.

## A73 Utility of the Pain Symptom Assessment Tool (PSAT) in assessing for fibromyalgia in JIA patients

### Melissa Tesher^271^, Mark Connelly^282^, Brent Graham^307^, Tracy Ting^66^, Jennifer Weiss^115^, for the CARRA Pain Committee

#### ^271^University of Chicago Medicine Comer Children's Hospital, Chicago, IL, USA; ^282^University of Missouri-Kansas City School of Medicine, Kansas City, MO, USA; ^307^Vanderbilt University Medical Center, Nashville, TN, USA; ^66^Cincinnati Children's Hospital, Cincinnati, OH, USA; ^115^Hackensack University Medical Center, Hackensack, NJ, USA

##### **Correspondence:** Melissa Tesher

Background : Pain in children with JIA has a significant impact on patient quality of life. Persistence of significant patient-reported pain despite normal objective measures of disease activity poses a quandary for the pediatric rheumatologist. The Pain Symptom Assessment Tool (PSAT) is a modified version of the 2010 ACR fibromyalgia criteria, and has demonstrated good specificity and sensitivity for diagnosis of juvenile primary fibromyalgia syndrome in adolescents. We aim to: 1. Evaluate the utility of the PSAT in identifying JIA patients with comorbid juvenile fibromyalgia (JFM) and 2. Identify differences amongst JIA patients with and without JFM including potential variables such as demographic data, disease characteristics, functional disability, and physician and patient/parent global assessments.

Methods : Children and adolescents ages 11-17, with a diagnosis of JIA, were eligible for the study. Consented patients completed the PSAT, Functional Disability Index (FDI), pain intensity visual analog scale, the Pain Catastrophizing Scale for Children (PCS-C), a demographics form, and global assessment during routine rheumatology clinic visits. Physicians completed an active joint count, global assessment, and tender point exam.

Results : Data collection was completed in December 2018. 139 child and adolescent subjects (74% female) were enrolled. All JIA subtypes were represented in the study population. 14 patients (10%) met criteria for JFM using the PSAT. Subjects meeting PSAT criteria for JFM had markedly higher FDI in comparison to the mean FDI among subjects not meeting criteria (see Table 1). These patients also had a higher mean and clinically meaningful (≥ 30) pain catastrophizing scale score. 97 subjects had a tender points exam performed. Among these, 32 patients were found to have at least 5 tender points on exam, of whom 22% met PSAT criteria for fibromyalgia.

Conclusions : Preliminary data analysis identified a relatively low prevalence (10%) of comorbid JFM among JIA patients as measured by PSAT. JIA patients who screened positive for JFM demonstrated markedly high levels of functional disability and pain catastrophizing.

This study was approved by the Institutional Review Board at the University of Chicago Medical Center, Vanderbilt University Medical Center, Cincinnati Children’s Hospital Medical Center, and Hackensack University Medical Center.

Acknowledgements: This project was funded by a CARRA – Arthritis Foundation grant. The authors wish to acknowledge the ongoing Arthritis Foundation financial support of CARRA.

**Table 1 (abstract A73).** Comparison of JIA patients who do and do not meet criteria for JFM via PSAT

	JFM+ via PSAT n (%)	JFM – via PSAT n (%)
Total Patients	125 (90)	14 (10)
Female (%)	103 (74)	12 (85.7)
JIA Subtype, No (%)		
Polyarticular	44 (32)	5 (36)
Oligoarticular	38 (27)	1 (7)
Enthesitis related	17 (12)	3 (21)
Psoriatic	3 (21)	3 (21)
Unclassified	18 (13)	4 (29)
FDI Parent (mean)	43.8	20.4
FDI Patient (mean)	47.1	20.5
PSC-C (mean)	30.4	15
Tender points (mean)	8	2.85

Pain Catastrophizing Scale for Children (PCS-C); Functional Disability Index (FDI)

## A74 Development and Evaluation of a Research Coordinator Mentoring/Coaching Program

### Y. Ingrid Goh^229^, Jennifer Woo^266^, Anne Dennos^95^, Chelsey Smith^61^, Joanne Drew^152^, Bipin Mala^238^, Marsha Malloy^144^, on behalf of the CARRA Research Coordinators

#### ^229^The Hospital for Sick Children, Toronto, ON, Canada; ^266^University of California Los Angeles, Los Angeles, CA, USA; ^95^Duke University, Durham, NC, USA; ^61^Children's Mercy Hospitals and Clinics, Kansas City, MO, USA; ^152^Nationwide Children's Hospital, Columbus, OH, USA; ^238^Tufts Medical Center, Boston, MA, USA; ^144^Medical College of Wisconsin, Wauwatosa, WI, USA

##### **Correspondence:** Y. Ingrid Goh

Background : Research coordinators are crucial personnel who support clinical research operations, such as maintaining regulatory documentation, conducting accurate data entry, and recruiting subjects. Coordinators who are unfamiliar with clinical research processes, new to CARRA, or who are the sole individual supporting operations at their site may benefit from having a mentor. Coordinator mentorship/coaching program (CMCP) offers multifaceted education and career mentoring. The objective of this project is to develop, pilot and evaluate CMCP.

Methods : Prior to CMCP development, the Childhood Arthritis Rheumatology Research Alliance (CARRA) Research Coordinator Advisory Committee (RCAC) performed a literature review of CMCP programs and consulted with a certified faculty-level executive coach and CARRA leadership. Subsequently, a needs assessment was conducted among research coordinators to identify desired program aspects. All CARRA coordinators (N=183) were asked about their interest in participating in the program as a mentor or mentee. Interested individuals were matched based on the mentee’s identified goals and the mentor’s area of expertise. Matches received an introduction email detailing the goals of the program and were encouraged to contact each other as well as to ask the RCAC for support at any time. The RCAC sent mentors and mentees a follow-up email to assess satisfaction and progress approximately 1 month and 4 months after the match. Data were analyzed using descriptive statistics.

Results : Approximately 27% (n=50) of coordinators responded to multiple survey inquiries about participation in CMCP. Fourteen respondents indicated interest in being a mentee, 9 as mentors, and 3 as both a mentee and a mentor. Four of the 14 respondents who originally indicated an interest in receiving mentorship declined to participate prior to being matched. Reasons cited for change in interest were: have an onsite mentor/coach, no longer working on CARRA projects and “not good timing”. Mentor/mentee pairs were connected at 10 sites. Feedback from mentors and mentees has been positive at both 1- and 4-month time points. All coordinators who participated in the initial 6-month match period have remained at their active CARRA Registry sites. One mentored coordinator achieved their goal of becoming a Society of Clinical Research Associates-certified coordinator.

Conclusions : This pilot CMCP provided resources to aid coordinators/sites to achieve their mentoring/mentee/coaching goals. Further directions include expanding CMCP to include additional research coordinators and to assist investigators who have limited research coordinator support.

This study is a program evaluation and is under consideration for exemption of human subjects research by the IRB at the University of Wisconsin-Milwaukee.

Acknowledgments: The authors would like to acknowledge Karen Marc Dante, MD, Medical College of Wisconsin, who is a professional coach certified by the Executive Coach program, Kelly Mieszkalski and Miranda Wenzlaff who provided executive-level guidance for the development of the coordinator mentorship/coaching program and members of the CARRA Research Coordinator Advisory Committee (RCAC): Heather Benham, Joni Dean, Kim Francis, Ching Hung, Suzy Jones, and Mary Ellen Riordan who contributed tangentially to the project. The authors wish to acknowledge CARRA, and the ongoing Arthritis Foundation financial support of CARRA.

## A75 The Intersection between Pain, Fatigue, and Cardiovascular Risk in Teens with Juvenile Idiopathic Arthritis: A Metabolomics Pilot Study

### Kimberly Lewis^210^, Nicole Osier^236^, Stefano Tiziani^234^, Ruy Carrasco^80^, Patricia Carter^234^, Alexandra Garcia^236^, Shelby Brooks^180^, Janet Orrock^80^, Lauren Connolly^210^

#### ^210^Seton Healthcare Family/The University of Texas at Austin, Austin, TX, USA; ^236^The University of Texas at Austin/Dell Medical School, Austin, TX, USA; ^234^The University of Texas at Austin, Austin, TX, USA; ^80^Dell Children's Medical Center, Austin, TX, USA; ^180^Patient

##### **Correspondence:** Kimberly Lewis

Background : Patients with JIA are at higher risk of cardiovascular morbidity and mortality than the general population, which may be related to the inflammatory nature of the disease process. Teens with JIA are exposed to inflammation both during critical development periods and for a longer duration of time than people who develop chronic conditions in adulthood. The purpose of this pilot study is to explore the metabolomic signature associated with JIA in teens, and to differentiate the cardiovascular risk phenotype. A secondary purpose is to describe the relationships between pain and fatigue symptoms, diet, physical activity, environmental pollution, and cardiovascular risk factors.

Methods : N=40 children (n=20 children with JIA age 13-17 and n=20 controls) will be recruited from the ‘Specially for Children Pediatric Rheumatology at Dell Children’s Medical Center in Austin, TX. Participants and parents complete surveys, medical history, and assessments [Visual Analog pain scale, PROMIS pain interference scale, AHA CV risk factors, PROMIS fatigue scale, PROMIS physical activity (PA), JIA history, Physician’s Global Assessment of Disease Activity, 24 hour dietary recall, vital signs, height, weight, and BMI percentile, demographics, pollution levels by zip code]. Fasting blood and urine samples are collected and processed. Once all samples have been collected, blood will be processed using validated methods of nuclear magnetic resonance and/or mass spectroscopy to differentiate the phenotype and identify the metabolomic signature. Survey data will be analyzed using descriptive statistics, Pearson’s correlations, and t-tests.

Results : This work is currently in progress. Thirty percent of total patients have been recruited to-date (55% of the control group, 5% of JIA group). Successful recruitment strategies for healthy teenagers has included: enrolling during school holidays, encouraging teens who know each other to come together, and mailing surveys and documents in advance of the visit. Including a patient in the research team has also led to additional patient and control referrals and enrollment. A majority of the control group have been recruited from the PI’s existing social networks. Challenges with enrollment include: scheduling the fasting, morning blood draw which often overlaps with school or parent work schedules and screen failures.

Conclusions : We expect to find an indication that there is a metabolic difference between cardiovascular risk phenotypes that warrants further study in larger samples, and that there are relationships between variables. Identifying and enrolling teen control patients can be challenging, but creative recruitment strategies have been utilized in this study.

This study received Institutional Review Board Approval (2017-03-0042) from the University of Texas at Austin Institutional Review Board prior to enrollment and research activities. All participants and parents provided informed assent and consent prior to participating in the research activities.

Acknowledgments: This project was funded by a CARRA – Arthritis Foundation grant. The authors wish to acknowledge the Arthritis Foundation’s ongoing financial support of CARRA.

## A76 Correlates of Health Self-management among youth with jSLE: An update

### Kiana Johnson^97^, Anna Richmond^306^, Rich Vehe^281^, Eyal Muscal^26^, Kathleen O'Neil^129^

#### ^97^East Tennessee State University, Johnson City, TN, USA; ^306^Vanderbilt University, Nashville, TN, USA; ^281^University of Minnesota, Minneapolis, MN, USA; ^26^Baylor College of Medicine/Texas Children's Hospital, Houston, TX, USA; ^129^Indiana University School of Medicine, Riley Hospital for Children, Indianapolis, IN, USA

##### **Correspondence:** Kiana Johnson

Background : Although outcomes among youth with lupus have improved significantly over the last 20 years, these advances have outpaced our system’s capacity to effectively support these youth as they transition to adulthood and adult healthcare. Whether measured as access to quality healthcare, educational, vocational or quality of life outcomes, too many young adults with SLE are faring poorly. But it need not be so. The ultimate goal of this study is to examine factors explaining self-management skills and transition readiness for youth with jSLE in the context of the dynamic interaction of personal youth, family and healthcare system factors. For the purposes of this abstract, we report only preliminary results of surveys 67 patients with jSLE. We anticipate being able to present additional results from our sample which includes linked EMR data of youth from four different institutions (~200 youth with jSLE).

Methods : Participants include 67 youth with jSLE from two pediatric rheumatology clinics. Participants completed several surveys and using RedCap. De-identified data from the electronic medical record (EMR) was linked with participants’ responses on the survey. Survey measures include the TRAQ with revised response categories in accord with social loafing theory. Bivariate correlation analysis (Pearson’s r) were conducted. Regression analyses will be conducted to examine determinants of health self-management among youth with lupus using TRAQ scores, health self-efficacy, and disease knowledge (STARx) as predictors and patient’s SLEDAI score, hospital admissions, and ER visits as dependent variables. This study was approved by the Institutional Review Board of Vanderbilt University and East Tennessee State University (IRB# 161674). All psychometric information for instruments utilized are provided in Table 1. At the time of abstract submission, demographic information had not been linked to the de-identified database in RedCap.

Results : Our analyses demonstrated all scales to have good to excellent reliability. Significant correlations with moderate to strong effect sizes were observed for the associations between self-rated health and health self-efficacy, and social loafing. Additionally, Significant correlations with moderately strong effect sizes were demonstrated for the associations between social loafing and disease knowledge (STARx) and Task value. Further analyses will be conducted using multiple regression to predict independent health self-management.

Conclusions : These preliminary analyses show that for youth with jSLE self-efficacy was moderately related with poorer health; youth who reported poor health also reported had low self-efficacy scores for managing their health. Youth who reported poorer health also reported a lack of independent health self-management as indicated by the social loafing TRAQ scores. Lastly, youth with jSLE who reported more independent health self-management, as indicated by the social loafing TRAQ, also reported more disease knowledge and task value for managing their health. Once EMR data is released and linked to the data on 1/15/2019, we will conduct regression analyses to examine how and whether these variables serve to predict clinical health outcome variables (SLEDAI scores, ER visits, and hospital admissions) controlling for demographic variables (age, sex, duration of jSLE, and insurance status).

This study was approved by the Institutional Review Board of Vanderbilt University, Indiana University, and East Tennessee State University (IRB# 161674).

**Table 1 (abstract A76).** Properties of Study Measures

Measures	
Health	
range	1-4
mean(sd)	2.33(.80)
Health Self-efficacy	
range	1-5
mean(sd)	2.4(1.3)
Social Loafing TRAQ	
range	1-5
mean(sd)	3.3 (.47)
reliability	0.87
Task Value	
mean(sd)	4.4(.49)
reliability	0.81

**Table 2 (abstract A76).** Bivariate Correlations of study variables

	Health self-efficacy	SLTRAQ	STARx	Task Value
Poor health (higher scores on health variable indicate poorer health)	-.61**	-.53*	-.24*	-.43
Health self-efficacy		.26	.21	.40
SLTRAQ	—	—	.59*	.47*
StarX	—	—	—	.34*

## A77 Research Coordinator Efforts and Impact on Participant Recruitment in the CARRA Registry: 2015-2018

### Y. Ingrid Goh^229^, Anne Dennos^95^, Jennifer Woo^266^, for the CARRA Registry Investigators

#### ^229^The Hospital for Sick Children, Toronto, ON, Canada; ^95^Duke University, Durham, NC, USA; ^266^University of California Los Angeles, Los Angeles, CA, USA

##### **Correspondence:** Y. Ingrid Goh

Background : Creating and maintaining a robust registry requires a lot of effort. Launched in 2015, the Childhood Arthritis and Rheumatology Research Alliance’s (CARRA) latest registry plans to follow over 10,000 patients to learn about the progression of pediatric rheumatic conditions and medication safety. One of the factors which may impact the registry’s success is the availability of research coordinators to support each site’s activities. The objective of this project is to determine whether there is an association between indicators of research coordinator effort and participant enrollment in the Registry.

Methods : Registry enrollment numbers and data on research effort for all CARRA sites were obtained from CARRA’s coordinating center. The effects of the number of coordinators, length of coordinator time, and lapse in research coordinator support on the rate of participant enrollment into the Registry were assessed.

Results : Sixty-four CARRA sites were established between 2015-2018. 272 research coordinators contributed 370 service years across all sites. Research coordinators worked on the Registry for an average of 16 months (range: 9 days – 3.4 years). Although all sites reported at least one research coordinator, 14 sites reported a lapse in coordinator support ranging from 9 to 216 days. The average recruitment rates for sites with and without a lapse in research coordinator support were 37.1 and 43.5 participants per year, respectively. The annual recruitment rate was positively correlated with average length of research coordinator support, longest duration of research coordinator support by a single research coordinator, and total amount of research coordinator support after controlling for duration of Registry participation, as well as the number of research coordinators at a site. Annual recruitment rate was negatively correlated with longer lapses in research coordinator support.

Conclusions : Coordinator support contributes greatly to participant enrollment in the Registry. Increased research coordinator support, whether in duration or magnitude, is correlated with higher rates of participant recruitment. Strategies to support recruitment and retention of coordinators such as the CARRA Coordinator Pilot Program and mentorship programs would likely contribute to growth in annual Registry recruitment.

The study was approved by Duke Health Internal Review Board, approval number Pro00101777.

Acknowledgement: This work could not have been accomplished without the aid of the following organizations: The NIH’s National Institute of Arthritis and Musculoskeletal and Skin Diseases (NIAMS) & the Arthritis Foundation. We would also like to thank all participants and hospital sites that recruited patients for the CARRA Registry.

## A78 Assessing Oral Health and Microbiome Dysbiosis in Juvenile Dermatomyositis

### Albert Chow^207^, Peggy Lee^300^, Elizabeth Velan^207^, Pamela Gardner^162^, Laurie Brenchley^162^, Roger Bumgarner^301^, Mary Eckert^205^, Formosa Huang^301^, Nastaran Bayat^163^, Rita Volochayev^163^, Sharon Jackson^164^, Adam Schiffenbauer^163^, Lisa Rider^163^, Anne Stevens^207^

#### ^207^Seattle Children's Hospital, Seattle, WA, USA; ^300^University of Washington, School of Dentistry, Seattle, WA, USA; ^162^NIH, National Institute of Dental & Craniofacial Research, Bethesda, MD, USA; ^301^University of Washington, Seattle, WA, USA; ^205^Seattle Children’s Research Institute, Center for Immunity & Immunotherapies, Seattle, WA, USA; ^163^NIH, National Institute of Environmental Health Sciences, Bethesda, MD, USA; ^164^NIH, National Institute on Minority Health and Health Disparities, Bethesda, MD, USA

##### **Correspondence:** Albert Chow

Background : Juvenile dermatomyositis (JDM) is a rare and serious systemic autoimmune disease characterized by typical rashes and proximal muscle weakness. The etiology of JDM is unknown, and no studies have explored the link between oral dysbiosis and JDM. A variety of oral manifestations have been described, such as gingival telangectasia, similar in appearance to dilated nailfold capillary loops. Gingival inflammation suggests oral pathogens may be associated with a systemic inflammatory response. Previous studies investigating the relationship between the microbiome and autoimmunity have implicated dysbiosis in dental plaque as a factor in the pathogenesis of chronic inflammatory diseases such as rheumatoid arthritis. We hypothesize that children with JDM have an altered oral microbiome compared to their healthy siblings and parents. This study aims to identify the microbial communities specific to JDM patients in the salivary and plaque microbiome by examining the frequencies and diversity of organisms, and to evaluate the oral health of JDM patients in order to test for associations with disease activity or with specific organisms that may trigger inflammation via mucosal epithelial signals.

Methods : Twenty JDM families will be recruited at Seattle Children’s Hospital (SCH) and ten JDM families at the National Institutes of Health (NIH). Research visit procedures consist of a patient-reported oral health questionnaire, validated Manual Muscle Testing (MMT8), Cutaneous Dermatomyositis Disease Area & Severity Index (CDASI), oral dental exam by a dentist, and collection of saliva and supragingival/subgingival plaque for bacterial 16S rRNA sequencing. Relative frequencies as percentages of bacterial genera and species will be calculated. Statistical plan will include comparing patient-sibling dyads and patient-parent dyads. The microbiome diversity will be described and rarefaction will be plotted for each group. Comparisons and differences in microbiome diversity will be quantified by unweighted Jaccard and PERANOVA.

Results : Nineteen JDM families have been consented at SCH. 13 have completed study visits at SCH and 4 have completed study visits at the NIH. MMT8 scores were abnormal in 8 patients, median of 80 [interquartile range of 77 - 80]. Six JDM patients had abnormal nailfold capillaries and 12 patients had abnormal CDASI scores: median of 2 [0 - 5] for Activity, median of 1 [0 - 2] for Damage; 3 had normal MMT scores. The median gingival index was 0.3 [0.1 – 0.7] for JDM patients and 0.5 [0.1 - 1.4] for unaffected siblings. The median plaque index was 1.1 [0.6 – 1.4] in JDM patients and 1.1 [0.6 – 1.5] for unaffected siblings. Samples have been collected from 18 patients and 44 family members.

Conclusions : The feasibility of a pediatric rheumatology/dental collaborative study of the oral microbiome has been established. The remaining JDM families will complete study visits at SCH and NIH this winter. Bacterial DNA will be sequenced in the spring. The results of this study will inform the role of the microbiome in systemic inflammation in JDM.

This study was approved by the Seattle Children’s and NIDDK/NIAMS Human Subjects Committees.

This project was funded by a CARRA-Arthritis Foundation grant. The authors wish to acknowledge the Arthritis Foundation’s ongoing financial support of CARRA.

## A79 Development of a Protocol and Recruitment Materials to Support Recruitment for a Multi-site Study

### Y. Ingrid Goh^229^, Vincent Del Gaizo^41^, Alexandra Sirois^229^, Jennifer Weiss^115^, Mary Ellen Riordan^137^, Eyal Muscal^26^, Chivon McMullen-Jackson^23^, C.J. Inman^294^, Suzy Jones^296^, Jennifer Huntington^296^, Brian Feldman^229^, for the CARRA Investigators

#### ^229^The Hospital for Sick Children, Toronto, ON, USA; ^41^CARRA Inc., Milwaukee, WI, USA; ^115^Hackensack University Medical Center, Hackensack, NJ, USA; ^137^Joseph M. Sanzari Children's Hospital, Hackensack University Medical Center, Hackensack, NJ, USA; ^26^Baylor College of Medicine/Texas Children's Hospital, Houston TX, USA; ^23^Baylor College of Medicine, Houston, TX, USA; ^294^University of Utah, Salt Lake City, UT, USA; ^296^University of Utah Hospitals and Clinics, Salt Lake City, UT, USA

##### **Correspondence:** Y. Ingrid Goh

Background : Patients’ and caregivers’ decisions to participate in pediatric clinical research studies are influenced by numerous factors. Being faced with this decision immediately after receiving a diagnosis can be extremely difficult. We previously proposed to develop a study to help research teams understand how to better engage newly diagnosed patients with rheumatic conditions and their caregivers to join pediatric rheumatology research. The objective of the following is to provide a status update on this project.

Methods : We reviewed best practices documents regarding the engagement of patients in research. In addition, we reviewed studies examining factors which influence patients' and caregivers' decisions to participate in research in pediatric medical conditions other than rheumatology. A study protocol was designed based on the lessons learned from the review. Furthermore, materials to assist with recruitment across the multiple sites were developed.

Results : The literature review revealed the importance of including patients and caregivers in the development of the project. We invited two patient/caregiver representatives to join the study team, which is comprised of staff from four Childhood Arthritis and Rheumatology Research Alliance (CARRA) sub-sites. Together, the team developed the protocol and study materials. Moreover, an informational video was developed to standardize and support the informed consent process across multiple centres. The study has been submitted to local ethics boards for approval.

Conclusions : The collaboration of multiple research sites, patients, and caregivers has enabled the development of a study protocol and recruitment materials which will help researchers improve their understanding of factors which influence enrollment of newly diagnosed patients. We hope to commence study recruitment and data collection in the near future.

The study has been submitted and is under review at The Hospital for Sick Children's Research Ethics Board, REB # 1000060143.

Acknowledgment: This project was funded by a CARRA – Arthritis Foundation grant. The authors wish to acknowledge the ongoing Arthritis Foundation financial support of CARRA

## A80 Baseline Features and Outcomes of Pediatric-Onset Discoid Lupus Erythematosus: Interim Data Analysis of a Multicenter Retrospective Cohort Study

### Kaveh Ardalan^15^, Lisa Arkin^302^, Kevin Buhr^302^, Cordellia Nguyen^302^, Heather Brandling-Bennett^299^, Leslie Castelo-Soccio^55^, Yvonne Chiu^144^, Benjamin Chong^290^, Lucia Diaz^79^, Marisa Klein-Gitelman^15^, Amy Paller^168^, Jennifer Schoch^277^, Emily von Scheven^244^, Victoria Werth^286^, Julie Grossman-Kranseler^299^, Andrew Hudson^222^, Erin Ibler^144^, Mariana Marques^15^, Reesa Monir^277^, Elana Putterman^55^, for the CARRA Investigators

#### ^15^Ann & Robert H. Lurie Children's Hospital of Chicago, Chicago, IL, USA; ^302^University of Wisconsin School of Medicine and Public Health, Madison, WI, USA; ^299^University of Washington School of Medicine, Seattle, WA, USA; ^55^Children's Hospital of Philadelphia, Philadelphia, PA, USA; ^144^Medical College of Wisconsin, Wauwatosa, WI, USA; ^290^University of Texas Southwestern Medical Center, Dallas, TX, USA; ^79^Dell Children's Hospital, Austin, TX, USA; ^168^Northwestern University Feinberg School of Medicine, Chicago, IL, USA; ^277^University of Florida School of Medicine, Gainesville, FL, USA; ^244^University of California, San Francisco, San Francisco, CA, USA; ^286^University of Pennsylvania / Philadelphia Veterans Administration Medical Center, Philadelphia, PA, USA; ^222^Texas Tech University Health Sciences, Lubbock, TX, USA

##### **Correspondence:** Kaveh Ardalan

Background : Discoid lupus erythematosus (DLE) is rare in children. Prior studies suggest 25-30% of children with skin-limited DLE are diagnosed with systemic lupus erythematosus (SLE) over time. Biomarkers and risk factors to identify those at highest risk are unknown. This multicenter, retrospective study aims to characterize baseline features and outcomes in pediatric patients with skin-limited DLE, as well as risk factors for the progression of DLE to SLE. Baseline characteristics of all patients with DLE and initial study findings are presented in this interim analysis.

Methods : Nine of 18 committed clinical sites, including pediatric dermatologists and rheumatologists, retrospectively reviewed all medical records of patients =/<18 years of age with clinical and/or histopathologic findings consistent with DLE. Baseline data were collected on all patients, including demographics, dates of DLE onset and diagnosis, distribution of DLE, and family history of SLE. For patients with skin-limited DLE, rates of progression to SLE based on American College of Rheumatology (ACR) and Systemic Lupus International Collaborating Clinics (SLICC) classification criteria were evaluated.

Results : Clinical records for 205 patients have been reviewed to date, with 50% of participating sites reporting. Baseline data are presented in Table 1. African-American females were most commonly affected. Median age at DLE diagnosis was 11.8 years and median time from DLE onset to DLE diagnosis was 0.5 years. Most patients (76%) had localized disease (i.e. head/neck only); 20% had a family history of SLE in a 1st degree relative. Most patients had skin-limited DLE at baseline, with only a minority exhibiting >/=4 ACR classification criteria (n = 56; 27%) or >/=4 SLICC classification criteria (n = 46; 22%). Initial treatments are presented in Table 2. Patients with skin-limited DLE and at least one follow-up visit (n=115) had median follow up of 3.1 years (range 0.1-12.5 years, 393 total patient-years). During this period, a minority met criteria for SLE diagnosis, utilizing >/=4 ACR classification criteria (n = 16; 14%) and >/=4 SLICC classification criteria (n = 24; 22%).

Conclusions : This study represents the largest investigation of pediatric DLE performed to date. Utilizing both ACR and SLICC classification criteria for SLE, most patients (>73%) presented with skin-limited DLE, with a low cumulative incidence of SLE using both ACR and SLICC classification criteria. Further analysis may help to determine risk factors for progression to SLE and inform the creation of consensus guidelines for treating children with DLE.The study was approved by University of Wisconsin Madison Institutional Review Board (IRB 2017-0285-CR001).

Acknowledgements: This work has been supported by grant funding from the Childhood Arthritis and Rheumatology Research Alliance (CARRA) – Arthritis Foundation and the Pediatric Dermatology Research Alliance (PeDRA) (Co-PIs: Dr. Arkin and Dr. Ardalan). The authors wish to acknowledge the ongoing Arthritis Foundation financial support of CARRA.

**Table 1 (abstract A80).** Baseline data for 205 patients with DLE (skin-limited DLE and SLE)

	*N (%)*
Gender	
Female	142 (69%)
Male	63 (31%)
Race/Ethnicity*	
Black or African American	82 (40%)
White	61 (30%)
Hispanic or Latino	34 (17%)
Asian	14 (7%).
Native Hawaiian or Other Pacific Islander	2 (1%)
American Indian or Alaskan Native	1 (0.5%)
Unknown or not reported	15 (7%)
1^st^ degree family member with diagnosis of SLE	42 (20%)
Distribution of DLE lesions	
Localized (head/neck only)	155 (76%)
Generalized (both above/below the neck)	46 (22%)
Isolated (below the neck ONLY)	4 (2%)
	*Median (IQR)*
Age at presentation of DLE, years	10.8 (7.2-13.6)
Age at DLE diagnosis, years	11.8 (8.0-14.4)

*Participants could designate more than 1 race/ethnicity category

**Table 2 (abstract A80).** Medications at baseline for patients with DLE (Skin-Limited and SLE). Topical steroids and hydroxychloroquine were most frequently prescribed

Medication	*N, (%)*
Topical steroids	132 (65%)
Hydroxychloroquine	118 (58%)
Other immunomodulatory medications	59 (29%)
• Prednisone/Methylprednisolone	46 (22%)
• Mycophenolate mofetil	16 (8%)
• Rituximab	5 (2%)
• Methotrexate	4 (2%)
• Dapsone	4 (2%)
• Azathioprine	4 (2%)
• Quinacrine	1 (0.5%)
• Thalidomide	1 (0.5%)
Topical calcineurin inhibitors	40 (20%)
None	13 (6%)

## A81 Subclinical Synovitis in Oligoarticular JIA

### Deirdre De Ranieri^169^, Edward Oberle^150^, Johannes Roth^283^

#### ^169^Northwestern University Feinberg School of Medicine, Chicago, IL, USA; ^150^ Nationwide Children's Hospital, Columbus, OH, USA; ^283^University of Ottawa, Ottawa, ON, Canada

##### **Correspondence:** Deirdre De Ranieri

Background : Many patients with Oligo-articular JIA are reclassified later in their disease course from persistent to extended Oligo JIA, based on the involvement of more joints. The prognosis of these children is similar to those with RF- Poly-articular JIA, with an increased risk of joint damage with subsequent disability and decreased quality of life. Early patterns of joint involvement are associated with more severe, erosive disease and the need for DMARDs. Elbows, wrists, ankles and knees are the most commonly implicated joints in JIA, with early involvement of the wrists and ankles signaling an increased risk of a poly-articular course. In the TREAT trial, it was found that in poly-articular disease, the earlier and more aggressively JIA is treated, the more likely patients are to go into sustained remission and not suffer long-term sequelae of disease. Patients with Oligo-articular JIA are at this point ineligible for biologic medications based on the number of joints involved. In RA, subclinical synovitis predicts relapse and disease flare but the data in children is unclear. If subclinical disease evolves clinically, earlier stratification of children into poly-articular subtype may render them eligible for more aggressive therapy earlier in their disease course. This is a pilot study of 40 children to sonographically assess for subclinical synovitis in patients with newly diagnosed Oligo-articular JIA.

Methods : Subjects: 40 patients ages 2-17 yr of age, with Oligoarticular JIA diagnosed <12 months ago. Data collection will occur at time points 0,3,6,9,12 mos and will age, sex, ANA status, +/-uveitis, medications, ESR and CRP. Elbows, wrists, knees, ankles will be examined and then ultrasounded. Outcome measures include JADAS, PQoL Arthritis, PGA (physician and parent), joint count (physician and parent), ESR & CRP.

Results : Data from one patient has been collected so far, given that the study just officially started. We will have more data at the time of the poster presentation. However, this patient has subclinical disease in both ankles and has received IACI into both knees and is currently asymptomatic off medication. Characteristics include ANA positive, no evidence of uveitis, er labs have been normal and there is nothing of significance noted in either bloodwork, questionnaires, Physician or Patient global assessment, or patient's perceived joint count.

Conclusions : Conclusions will be pending more enrollees. The project has officially started and we are now actively recruiting patients. By the time of the meeting, we will have more data to share. If there is preliminary evidence to suggest that ultrasound can positively predict disease progression, a larger-scale project would be warranted to confirm this finding, which could have significant down-stream effects, including more aggressive treatment with the hope of halting disease progression, decreasing disease duration and preventing joint damage in patients with JIA.

Approval Date: 07-09-2018 Expiration Date: 06-30-2019 Organization: Department of Pediatrics, Rheumatology Current Policy Pre-2018 Rule CONFIRMATION OF FUNDS IN NOVEMBER 2018.

This project was funded by a CARRA – Arthritis small grant. The authors wish to acknowledge the ongoing Arthritis Foundation financial support of CARRA.

## A82 Development of a Mobile App to Track Disease Activity in Juvenile Localized Scleroderma

### Suzanne Li^114^, Xiaohu Li^215^, Jiajun Chen^176^

#### ^114^Hackensack Meridian School of Medicine at Seton Hall University, Hackensack, NJ, USA; ^215^Stevens Institute of Technology, Hoboken, NJ, USA; ^176^One Node Lab, League City, TX, USA

##### **Correspondence:** Suzanne Li

Background : Aim: To generate a mobile app for aiding assessment of disease activity level in localized scleroderma (LS) patients. Juvenile LS (jLS) is associated with major morbidity for growing children including hemiatrophy, arthropathy, and seizures. Treatment is directed at controlling inflammation, with long-term monitoring needed because of disease chronicity and frequent relapses. Clinical examination is the primary method used to identify and determine the level of disease activity. The large variation in disease patterns and features makes this a challenging and often laborious task. A mobile app that aids the evaluation of jLS activity would facilitate and improve patient care.

Methods : This project is a collaboration between a clinician (SL), statistician (XL), and software engineer (JC). The app is being developed with EmguCV 3.4 professional version in Microsoft Visual Studio 2017 and Xamarin. The app will have two main features: to determine the extent of the lesion feature, and assess the level of erythema. Standards for scoring erythema levels will be based upon the jLS scoring atlas, which contains consensus photographs of each scoring level. Storage capability and scoring calculator will be built into the app, allowing the physician to input other lesion features, such as skin thickening.

Results : Two major obstacles have been encountered in our development of the mobile app. Image resolution was found to be substantially reduced when images were directly acquired from the camera app. Our work around is to have image acquisition occur in two steps; first the camera app photographs the lesion, then the LS app retrieves the images from the normal storage site of the camera. The other issue is related to the need for boundary detecting software that functions in a mobile app. The boundary detecting software we were using was developed as a desktop app and not found usable in the mobile environment. The code for this desktop app was converted with EmguCV, C#, and Xamarin to allow its use in a mobile device. To correctly measure lesion extent, in addition to identifying the lesion boundaries, we need to calibrate the effects of camera-object distance, and distortions caused by camera tilting and curved skin surfaces. We are using a photography color checker card to calibrate the effects of camera-object distance and camera tilting, and a geometric model to calibrate the distortion due to curved surfaces. The color checkercard is also being used to remove the color deviation due to differences in ambient lighting conditions as this would affect accurate assessment of erythema levels. Computer-generated geometries with standard color and known area are being used to evaluate the performance of these calibrations, to determine which to add to the app.

Conclusions : We have made progress towards developing a mobile app to assist the assessment, scoring, and tracking of disease activity in juvenile LS. Some issues have been identified, with more work needed to test and refine extent and then erythema assessment. A sensitive, easy to use app will improve our ability to care for and treat these patients, and facilitate conducting treatment trials.

Acknowledgements: The authors thank the ongoing Arthritis Foundation financial support of CARRA, and the support of Alpha Omicron Pi Foundation, Arthritis Foundation, and CARRA for funding for this project. We thank the patients and families for participating in this study.

## A83 Data Analysis Grant for the Juvenile Localized Scleroderma Consensus Treatment Plan Pilot Study

### Suzanne Li^114^, Gloria Higgins^151^, Kathryn Torok^287^, Ron Laxer^119^, Polly Ferguson^279^, C. Rabinovich^95^, Robert Fuhlbrigge^72^, Mara Becker^228^, Fatma Dedeoglu^32^, Elena Pope^119^, Maria Ibarra^228^, Thomas Mason^142^, Sandy Hong^279^, Katie Stewart^220^, Brian Feldman^229^, Vidya Sivaraman^151^, Marilynn Punaro^220^, Xiaohu Li^215^, Tracy Andrews^115^, for the CARRA Localized Scleroderma Subcommittee

#### ^114^Hackensack Meridian School of Medicine at Seton Hall University, Hackensack, NJ, USA; ^151^Nationwide Children’s Hospital, Columbus, OH, USA; ^287^University of Pittsburgh Medical Center, Pittsburgh, PA, USA; ^119^Hospital for Sick Kids, Toronto, ON, Canada; ^279^University of Iowa, Iowa City, IA, USA; ^95^Duke University, Durham, NC, USA; ^72^Colorado Children's Hospital, Aurora, CO, USA; ^228^The Children's Mercy Hospital, Kansas City, MO, USA; ^32^Boston Children’s Hospital, Boston, MA, USA; ^142^Mayo Clinic, Rochester, MN, USA; ^220^Texas Scottish Rite Hospital, Dallas, TX, USA; ^229^The Hospital for Sick Children, Toronto, ON, Canada; ^215^Stevens Institute of Technology, Hoboken, NJ, USA; ^115^Hackensack University Medical Center, Hackensack, NJ, USA

##### **Correspondence:** Suzanne Li

Background : Aim: To conduct data analysis on the juvenile localized scleroderma (jLS) Consensus Treatment Plan (CTP) pilot study data to generate three publications: 1. The process of how to work towards conducting comparative effectiveness studies in a rare disease. 2. The initial findings of the 1-year prospective observational jLS CTP pilot study 3. An evaluation of the performance of clinical assessment tools that had been developed to conduct treatment trials. Some of these tools had been developed empirically, based upon literature review and consensus methodology, and the jLS CTP Pilot study provided data to allow their evaluation. Background: JLS is a chronic rheumatic disease which often persists through childhood. Children are at risk for serious morbidity including arthropathy, hemiatropphy, and optical and neurological problems. The localized scleroderma (LS) workgroup of CARRA previously developed 3 methotrexate-based CTPs to work towards identifying optimal treatment.

Methods : Members of the LS workgroup from 10 CARRA centers planned and conducted the jLS CTP Pilot study, which was funded by an Arthritis Foundation Innovative Research Grant. The study was planned as a feasibility study, aimed at identifying issues with the developed CTPs and with conducting comparative effectiveness studies. A secondary aim was to evaluate recently developed jLS clinical assessment tools. Face-to-face meetings were held to help standardize clinical evaluation, finalize CRFs, and discuss study issues. Later, calls and meetings were held to discuss data analysis, plan manuscripts, and develop additional clinical tools. The CTP pilot study was a prospective, observational cohort study that followed 50 active juvenile LS patients initiating treatment with one of the 3 CTPs. Patients were evaluated at 6 study visits over 1 year using standardized measures. Study data was primarily collected into the web-based CARRA Legacy registry. Data from some assessment forms and patient questionnaires were separately collected into an excel file. The study began 3/2012, with final patient visit completed 5/2015.

Results : Much time was spent with data clean-up and review. Investigators were queried if items were missing or if there were inconsistencies in responses to some questions. Different analytical approaches were explored. Some of the study findings have been presented in poster and oral abstract presentations at annual ACR and CARRA meetings. The investigators are reviewing drafts of the first two manuscripts of the study. We expect the final versions of these manuscripts will be submitted in January. The majority of the analysis needed for the third manuscript has been completed.

Conclusions : The CARRA publication grant has enabled us to finish the desired analysis of the jLS CTP pilot study. We are nearing completion of the first two manuscripts, and expect the third manuscript will be completed in the near future.

Acknowledgments: Funding for additional data analysis provided by a CARRA Publication Grant.

Funding for the CTP Pilot study from an Innovative Research Grant from the Arthritis Foundation.

The CARRA Legacy Registry was supported by a grant from National Institute of Arthritis and Musculoskeletal and Skin Diseases of the National Institutes of Health under award Number RC2AR058934. The CARRA Legacy Registry was also supported by CARRA, Friends of CARRA, the Arthritis Foundation, and the Duke Clinical Research Institute.

